# Cofactor F_420_: an expanded view of its distribution, biosynthesis and roles in bacteria and archaea

**DOI:** 10.1093/femsre/fuab021

**Published:** 2021-04-14

**Authors:** Rhys Grinter, Chris Greening

**Affiliations:** Department of Microbiology, Monash Biomedicine Discovery Institute, Monash University, Clayton, VIC 3800, Australia; Department of Microbiology, Monash Biomedicine Discovery Institute, Monash University, Clayton, VIC 3800, Australia

**Keywords:** cofactor 420, redox chemistry, enzymology, cofactor biosynthesis, redox cofactor, cofactor distribution

## Abstract

Many bacteria and archaea produce the redox cofactor F_420_. F_420_ is structurally similar to the cofactors FAD and FMN but is catalytically more similar to NAD and NADP. These properties allow F_420_ to catalyze challenging redox reactions, including key steps in methanogenesis, antibiotic biosynthesis and xenobiotic biodegradation. In the last 5 years, there has been much progress in understanding its distribution, biosynthesis, role and applications. Whereas F_420_ was previously thought to be confined to Actinobacteria and Euryarchaeota, new evidence indicates it is synthesized across the bacterial and archaeal domains, as a result of extensive horizontal and vertical biosynthetic gene transfer. F_420_ was thought to be synthesized through one biosynthetic pathway; however, recent advances have revealed variants of this pathway and have resolved their key biosynthetic steps. In parallel, new F_420_-dependent biosynthetic and metabolic processes have been discovered. These advances have enabled the heterologous production of F_420_ and identified enantioselective F_420_H_2_-dependent reductases for biocatalysis. New research has also helped resolve how microorganisms use F_420_ to influence human and environmental health, providing opportunities for tuberculosis treatment and methane mitigation. A total of 50 years since its discovery, multiple paradigms associated with F_420_ have shifted, and new F_420_-dependent organisms and processes continue to be discovered.

## Teaser

This review provides a comprehensive description of the distribution and biosynthesis of the redox cofactor F_420_, as well as its enzymology, physiological roles and biotechnological applications.

## ABBREVIATIONS

2PL: 2-phospho-L-lactate
3PG: 3-phospho-D-glycerate
Adf: F_420_-dependent secondary alcohol dehydrogenase
ANME: anaerobic methanotrophic archaea
AOA: ammonium-oxidizing archaea
APDs: 4-alkyl-L-proline derivatives
BGC: biosynthetic gene cluster
CoM: coenzyme M
CoB: coenzyme B
CoB-S-S-CoM: coenzyme B, coenzyme M heterodisulfide
Ddn: deazaflavin-dependent nitroreductase
DFTR: F_420_H_2_-dependent flavin-containing thioredoxin reductase
DH-F_420_: dehydro-F_420_
EPPG: enolpyruvyl-diphospho-5’-guanosine
FAD: flavin adenine dinucleotide
FDOR(-A/B): flavin/deazaflavin oxidoreductase (subfamily A or B)
Ffd: F_420_-reducing formate dehydrogenase
Fgd: F_420_-reducing glucose-6-phosphate dehydrogenase
fHMAD: F_420_-dependent hydroxymycolic acid dehydrogenase
FMN: flavin mononucleotide
Fno: F_420_H_2_-dependent NADP reductase
FOP: F_O_-5′-phosphate
fPKR: F_420_H_2_-dependent phthiodiolone ketoreductase
Fpo: F_420_H_2_-dependent methanophenazine reductase
FprA: F_420_H_2_-dependent oxidase
Fqo: F_420_H_2_-dependent quinone reductase
FRET: Förster resonance energy transfer
Frh: F_420_-reducing hydrogenase
Fsr: F_420_H_2_-dependent sulfite reductase
G6P: glucose-6-phosphate
GPPG: glyceryl-diphospho-5‘-guanosine
H_4_MPT: tetrahydromethanopterin
CHO-H_4_MPT: 5-formyltetrahydromethanopterin
CH≡H_4_MPT: 5,10-methenyltetrahydromethanopterin
CH_2_=H_4_MPT: 5,10-methylenetetrahydromethanopterin
CH_3_-H_4_MPT: 5-methyltetrahydromethanopterin
LUCA: last universal common ancestor
LLHT: luciferase-like hydride transferase
LPPG: lactyl-diphospho-5‘-guanosine
MAGs: metagenome derived genomes
MDR: multidrug-resistant tuberculosis
Mer: methylene-H_4_MPT reductase
Mtd: methylene-H_4_MPT dehydrogenase
NADH: nicotinamide adenine dinucleotide
NADPH: nicotinamide adenine dinucleotide phosphate
NTR: nitroreductase
OYE: Old Yellow Enzymes
PBDs: pyrrolobenzodiazepines
PDIM: phthiocerol dimycocerosates
PEP: phosphoenolpyruvate
XDR: extensively drug-resistant tuberculosis

## INTRODUCTION

**Figure 1.**
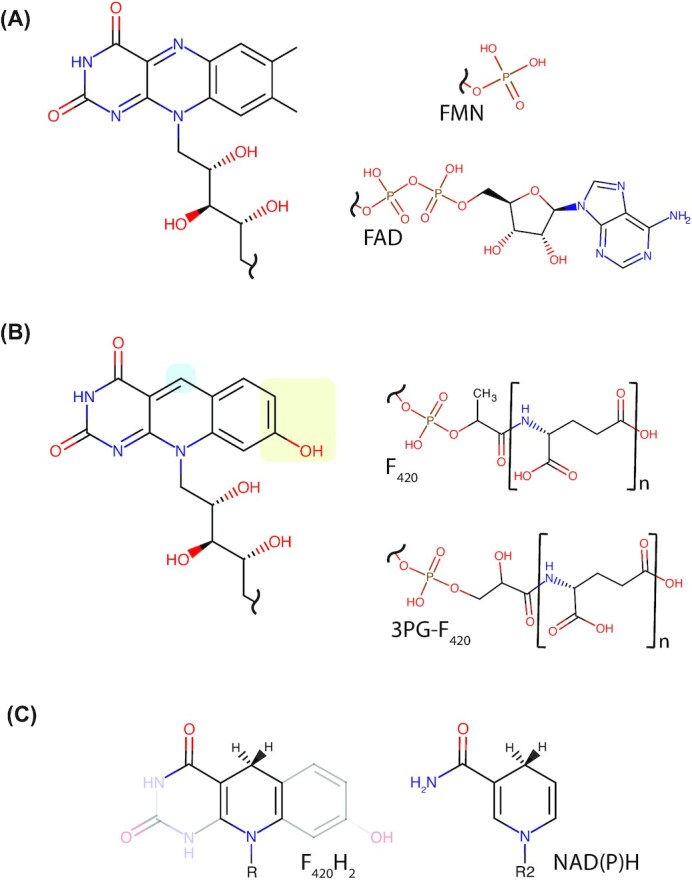
Structural comparison of F_420_ with flavins and nicotinamides. **(A)** Structures of the riboflavin head group and tail groups of the flavins FMN and FAD. **(B)** Structures of the 5-deazaflavin head group and tail groups of F_420_ and 3PG-F_420_. Locations of chemical substitutions between riboflavin and 5-deazaflavin are highlighted. *N* = 2–9 depending on the microbial species. **(C)** Structural similarity between the nicotinamides NAD(P)H and the central redox-active portion of F_420_H_2_. For F_420_H_2,_ R represents the phospholactyl and oligoglutamate tail shown in panel B. For NAD(P)H, R2 represents the ribose-5-phosphate of the nicotinamide nucleotide and the adenosine nucleobase as shown in (Bogan and Brenner 2013).

Cofactors play a fundamental role in biological chemistry. When bound to enzymes, they provide chemical reactivity and specificity that is otherwise unattainable via protein sidechain and backbone chemistry (Begley 2010). Cofactors that mediate redox reactions often contain heterocyclic ring structures, which can accept and donate electrons at physiologically relevant redox potentials (Eicher, Hauptmann and Speicher 2013). In addition to the important heterocyclic riboflavin cofactors FAD and FMN (Fig. 1A), bacteria and archaea produce the structurally related deazaflavin cofactor, F_420_ (Factor 420; Fig. 1B; Cheeseman, Toms-Wood and Wolfe 1972; Eirich, Vogels and Wolfe 1979; Walsh 1980; Joosten and van Berkel 2007; Ney *et al*. 2017a). While F_420_ structurally resembles FAD and FMN, it is chemically more similar to the nicotinamide cofactors NAD and NADP (Fig. 1C; Jacobson and Walsh 1984; Walsh 1986; de Poorter, Geerts and Keltjens 2005; Huang *et al*. 2012; Buckel and Thauer 2013). Like NAD(P), F_420_ functions as a cellular hydride carrier (Hendrickson and Leigh 2008). It is reduced by dedicated F_420_-reducing dehydrogenases, with low potential electrons provided by catabolic substrates or NADPH (Schauer and Ferry 1986; Purwantini and Daniels 1996; Berk and Thauer 1997; Warkentin *et al*. 2001; Bashiri *et al*. 2008; Allegretti *et al*. 2014). The resulting reduced cofactor, termed F_420_H_2_, is then utilized by diverse F_420_H_2_-dependent reductases to reduce substrates in both catabolic and anabolic pathways (Wang *et al*. 2013; Ahmed *et al*. 2015; Purwantini, Daniels and Mukhopadhyay 2016; Greening *et al*. 2017; Mascotti *et al*. 2018; Steiningerova *et al*. 2020).

F_420_ was first described in methanogenic archaea of the phylum Euryarchaeota (Cheeseman, Toms-Wood and Wolfe 1972; Tzeng, Bryant and Wolfe 1975) by the Wolfe group in 1971. Its production was subsequently shown to be universal among methanogenic Euryarchaeota and widespread among other members of this phylum (Eirich, Vogels and Wolfe 1979; van Beelen, Dijkstra and Vogels 1983; Lin and White 1986; De Wit and Eker 1987; Gorris and Voet 1991; Gorris 1994; Purwantini, Gillis and Daniels 1997). F_420_ biosynthesis genes are also encoded by diverse other archaea, including members of the TACK and Asgard archaeal superphyla (Evans *et al*. 2015; Kerou *et al*. 2016; Vanwonterghem *et al*. 2016; Ney *et al*. 2017a; Jay *et al*. 2018; Spang *et al*. 2019; Wang *et al*. 2019). Independent from its discovery in methanogens, F_420_ was isolated from antibiotic-producing streptomycetes belonging to the phylum Actinobacteria (Miller *et al*. 1960; McCormick and Morton 1982), and was then shown to be widely produced by members of this phylum, including all members of the genus *Mycobacterium* (Naraoka *et al*. 1984; Daniels, Bakhiet and Harmon 1985; Purwantini, Gillis and Daniels 1997). Outside of Actinobacteria, F_420_ biosynthesis genes have been detected in a diverse range of bacteria, and its production has been biochemically confirmed in both Proteobacteria and Chloroflexi (Ney *et al*. 2017a; Braga *et al*. 2019, 2020). Until recently, it was thought that the F_420_ biosynthesis pathway was identical in all producing organisms (Ney *et al*. 2017a). However, recent studies have uncovered variation in the substrates and enzymes utilized for F_420_ biosynthesis between bacteria and archaea, as well as a new variant of the mature cofactor in Proteobacteria (Bashiri *et al*. 2019; Braga *et al*. 2019; Grinter *et al*. 2020). This variability reflects the diversity of the organisms that produce F_420_, as well as the complex evolutionary history of the biosynthesis pathway, which is characterized by both vertical and horizontal gene transfer events (Weiss *et al*. 2016; Ney *et al*. 2017a).

In addition to its role in microbial physiology, F_420_ has garnered interest for its industrial, medical and environmental applications. The cofactor and its analogs have potential in industrial biocatalysis (Taylor, Scott and Grogan 2013; Greening *et al*. 2017; Bashiri *et al*. 2019; Drenth, Trajkovic and Fraaije 2019). The low redox potential and obligate hydride transfer chemistry of F_420_ enable reduction of otherwise recalcitrant organic molecules (Greening *et al*. 2017; Mathew *et al*. 2018; Martin *et al*. 2020). Numerous F_420_-dependent enzymes are present in microbial genomes, providing an inventory for industrial biocatalysis (Selengut and Haft 2010; Ahmed *et al*. 2015; Mascotti *et al*. 2018; Steiningerova *et al*. 2020). Some progress has been made towards use of F_420_-dependent enzymes in industrial catalysis, including the first heterologous production of the cofactor (Bashiri *et al*. 2019; Braga *et al*. 2019; Ney 2019), though further advances are required. With respect to medical applications, the F_420_-dependent enzyme deazaflavin-dependent nitroreductase (Ddn) from *Mycobacterium tuberculosis* activates the recently approved antitubercular drugs pretomanid and delamanid and F_420_ has been shown to play a role in antimicrobial resistance in mycobacterial pathogens (Hasan *et al*. 2010; Cellitti *et al*. 2012; Gurumurthy *et al*. 2013; Lee *et al*. 2020). Additionally, methanogenic archaea that reside in environments such as livestock rumen, rice paddies and waste landfill produce a significant portion of global methane emissions via a process that requires F_420_ (Kirschke *et al*. 2013; Greening *et al*. 2019). As such, inhibition of F_420_ biosynthesis or F_420_-dependent enzymes in livestock has been proposed as a strategy to reduce global greenhouse gas emissions (Attwood *et al*. 2011; Patra *et al*. 2017).

Significant progress has been made in understanding F_420_ in the five years since this topic was last reviewed comprehensively (Greening *et al*. 2016). We now have a much-improved understanding of the distribution, biosynthesis and roles of F_420_. These new findings have challenged several paradigms in the field, including the idea that F_420_ is restricted to a few microbial lineages and is synthesized through a universal pathway. This review provides a new synthesis of our understanding of F_420_, by integrating recent and historical literature while outlining remaining knowledge gaps. We also discuss how these fundamental advances facilitate applications, for example heterologous F_420_ production for biocatalysis.

## CHEMISTRY, DISTRIBUTION AND ROLES OF F_420_

### Chemical properties

Like the universal nicotinamide cofactors NAD(P) and flavin cofactors FMN/FAD, the primary role of F_420_ is to transfer electrons between compounds within the cell (Walsh 1986; Munro and McLean 2013). Chemically, F_420_ consists of three components: the redox-active isoalloxazine head group F_O_, a phospho-organic acid linker and a γ-linked polyglutamate tail of variable length (Fig. 1B; Eirich, Vogels and Wolfe 1978; Braga *et al*. 2019).

**Figure 2.**
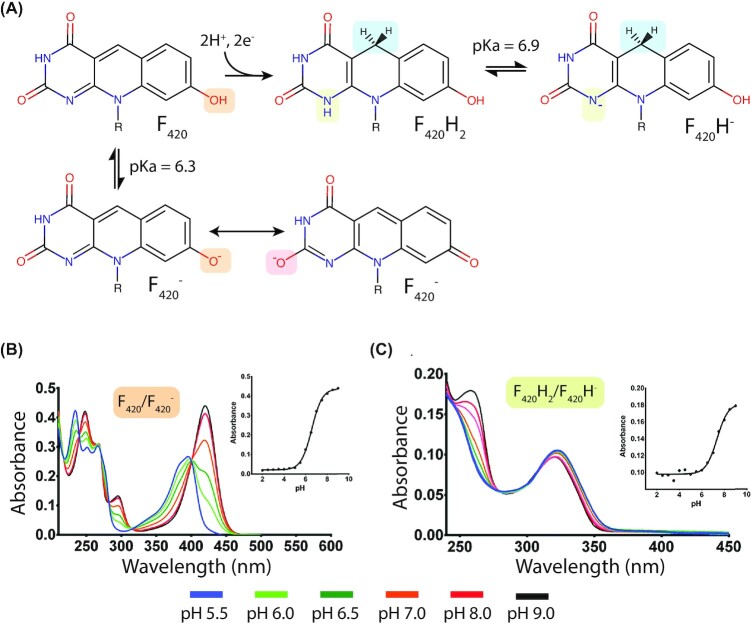
F_420_ protonaiton states, redox transitions and associated spectral shifts. **(A)** Changes in the protonation state of F_420_ and F_420_H_2_ as a result of the change in external pH. R = F_420_ tail group as depicted in Fig. 1B. **(B)** Spectral changes of F_420_ between pH 5.5 and 9.0 resulting from a change in protonation state depicted in panel A. Inset graph shows a change in absorbance at 420 nm. **(C)** Spectral change in F_420_H_2_ as in panel B. Inset graph shows changes in absorbance at 280 nm. Panels B and C are adapted from Mohamed *et al*. (2016a).

As a 5-deazaflavin moiety, the F_O_ head group contains three chemical substitutions compared to flavins (Fig. 1A and B) that give F_420_ unique spectral and electrochemical properties (Fig. 2A and B). The key change is the substitution of the redox-active N-5 atom of the isoalloxazine ring for a carbon. In contrast to flavins, this substitution precludes F_420_ from forming a stable semiquinone, given unpaired electrons cannot delocalize through a C-5 isoalloxazine ring in low-energy states (O'Brien, Weinstock and Cheng 1967, 1970; Edmondson, Barman and Tollin 1972; Xia, Shen and Zhu 2015). As a result, F_420_ is an obligate hydride carrier similar to nicotinamides and does not readily undergo single-electron reactions such as autooxidation in air (Fisher, Spencer and Walsh 1976; Spencer, Fisher and Walsh 1976; Jacobson and Walsh 1984; Walsh 1986). In addition, when compared to flavins, C-7 and C-8 of F_420_ are demethylated and C-7 is hydroxylated, further altering the redox properties of the cofactor (Eirich, Vogels and Wolfe 1978). As a result of these three substitutions, F_420_ has a much lower standard redox potential (−340 mV) than riboflavin (−210 mV), FAD (−220 mV) or FMN (−190 mV; Thauer, Jungermann and Decker 1977; Walsh 1986). This redox potential is modulated by physiological conditions, resulting in a redox potential of −380 mV in hydrogenotrophic methanogens that maintain a 10:1 ratio of reduced to oxidized F_420_ (Jacobson and Walsh 1984; de Poorter, Geerts and Keltjens 2005). This makes F_420_ well suited to mediate the low potential reactions of anaerobic metabolism, as well as reductions that require a low potential electron donor (Thauer, Jungermann and Decker 1977; Hartzell *et al*. 1985).

F_420_ can exist in a range of protonation states as summarized in Fig. 2. The resonance structure of the isoalloxazine ring of oxidized F_420_ lowers the p*K*_a_ of the C-7 hydroxyl group to 6.3, favoring its deprotonation under basic conditions. Deprotonation of the F_420_ C-7 hydroxyl leads to delocalization of the resulting unbonded electron and the formation of a conjugated paraquinoid anion, which is the species that exhibits the classic F_420_ spectral properties of strong absorbance at 420 nm (Fig. 2A; Walsh 1980, 1986). In this paraquinoid state, F_420_^−^ exhibits reduced electrophilicity, making it resistant to reduction via hydride acquisition at its C-5 carbon (de Poorter, Geerts and Keltjens 2005). Protonation of the F_420_ C-7 hydroxyl group results in a shift of its absorption maxima to ∼400 nm, as well as a decrease in the overall absorption coefficient (Fig. 2B; Mohamed *et al*. 2016a). During reduction in biological systems, F_420_ receives a hydride ion at its C-5 carbon with reductant derived from H_2_, glucose-6-phosphate (G6P), NADPH, or other low-potential electron donors, via the action of dedicated F_420_H_2_-dependent reductases (Fig. 2A; Aufhammer *et al*. 2004; Vitt *et al*. 2014; Le *et al*. 2015; Oyugi *et al*. 2018). N-1 of reduced F_420_ possesses an unbonded electron pair and a net negative charge, facilitating its protonation, hence the F_420_H_2_ nomenclature applied to the reduced compound (Jacobson and Walsh 1984; Walsh 1986). The p*K*_a_ for the proton association with N-1 of reduced F_420_H_2_ is 6.9, meaning that the deprotonated reduced form, F_420_H^−^, may be the physiologically relevant state of this cofactor in many F_420_H_2_-dependent reductases, which has mechanistic implications as discussed below (Mohamed *et al*. 2016a). The changes to the bond structure of the isoalloxazine ring of F_420_H_2_ lead to a corresponding change in its optical properties (Fig. 2A and C; Eirich, Vogels and Wolfe 1979; Walsh 1986; Mohamed *et al*. 2016a). F_420_H_2_ exhibits weak absorbance at 320 nm, with deprotonation to F_420_H^−^ causing minimal further changes to its absorption profile in the visible spectrum (Fig. 2C; Mohamed *et al*. 2016a). F_420_H_2_ formation interrupts conjugation across the isoalloxazine ring and isolates the benzenoid portion of the molecule, preventing deprotonation of the C-7 hydroxyl at physiological pH (p*K*_a_ 9.7; Walsh 1980, 1986; Jacobson and Walsh 1984).

F_420_ is a fluorescent molecule, named for the absorbance of its oxidized F_O_ head group at 420 nm, with corresponding fluorescence emission at 470 nm mediated by a π→π* transition upon photon absorption (Cheeseman, Toms-Wood and Wolfe 1972; Mohamed *et al*. 2016a). F_O_ spectral properties are blue-shifted relative to flavin and give F_420_ a characteristic golden-yellow color and blue-green fluorescence (Cheeseman, Toms-Wood and Wolfe 1972; Eirich, Vogels and Wolfe 1978). The blue-shifted fluorescence of F_O_ allows it to efficiently transfer photons to flavin via Förster resonance energy transfer (FRET). In addition to its incorporation into F_420,_ F_O_ is synthesized independently and its fluorescent properties are exploited by a class of DNA photolyases, which bind F_O_ and FMN as cofactors to mediate the reductive cleavage of DNA pyrimidine dimers (Malhotra *et al*. 1992; Tamada *et al*. 1997). F_O_-utilizing DNA photolyases are present in cyanobacteria, unicellular algae and possibly higher eukaryotes including *Drosophila* (Mayerl *et al*. 1990; Sancar 1990; Glas *et al*. 2009). Like F_O_, F_420_ exhibits analogous autofluorescence and these properties can be used to identify F_420_-producing organisms such as methanogens and mycobacteria by fluorescence microscopy (Doddema and Vogels 1978; Maglica, Özdemir and McKinney 2015; Lambrecht *et al*. 2017), or sort them by flow cytometry. However, F_420_ is not used by DNA photolyases and its physiological role appears to be restricted to acting as a redox cofactor (Sancar 1990; Kiontke *et al*. 2014; Greening *et al*. 2016).

While the F_O_ deazaflavin headgroup is solely responsible for F_420_ redox function, the phospho-organic acid linker and polyglutamate tail modulate cofactor functionality by imparting negative charge and mediating interactions with F_420_ dependent enzymes (Fig. 1B; Ney *et al*. 2017b). Bacterial F_420_-dependent enzymes from at least two superfamilies form electrostatic interactions with the phosphate group of the F_420_ linker via conserved motifs, enhancing their specificity for the cofactor (Ahmed *et al*. 2015; Purwantini, Daniels and Mukhopadhyay 2016). The polyglutamate tail of F_420_ varies in maximum length among producing organisms and exists as a population of different tail lengths from one to nine residues (Gorris and Voet 1991; Gorris 1994; Ney *et al*. 2017a, b). In archaea, the relative abundance of F_420_ with different tail lengths varies depending on culture conditions and growth phase, suggesting tail length may modulate F_420_ function (Peck 1989). Recently we investigated the effect of F_420_ polyglutamate tail length on the function of mycobacterial F_420_-dependent enzymes (Ney *et al*. 2017b). F_420_ containing both short (two) and long (five to eight) polyglutamate chains were compatible with these enzymes, though long-chain F_420_ bound these enzymes with six to 10-fold greater affinity. Chain length also significantly modulated the kinetics of the enzymes, with long-chain F_420_ increasing the substrate affinity (lower *K_m_*) but reducing the turnover rate (lower *k_cat_*). Molecular dynamics simulations indicated that F_420_-dependent enzymes make multiple dynamic electrostatic interactions with the F_420_-polyglutamate tail via conserved surface residues, likely explaining the observed differences in activity between short and long chain F_420_ (Ney *et al*. 2017b). These data suggest that variable F_420_ polyglutamate tail length may have evolved to modulate the activity of F_420_-dependent enzymes. Additionally, these findings have significant implications for the use of F_420_ in industrial applications, where a high catalytic turnover is likely to be desirable.

### F_420_-dependent enzymes

F_420_-dependent enzymes are broadly classified as F_420_-reducing dehydrogenases or F_420_H_2_-dependent reductases based on the direction of the redox reaction they perform under physiological conditions (Greening *et al*. 2016). However, due to the relatively similar redox potentials of many F_420_-substrate pairs, some F_420_-dependent enzymes are bidirectional depending on the organism and physiological conditions (Eker, Hessels and Meerwaldt 1989; Berk and Thauer 1997; Afting, Hochheimer and Thauer 1998; Hendrickson and Leigh 2008). F_420_-dependent enzymes can be further divided into two classes based on their mechanism of electron transfer. In the first of these classes, bound F_420_ accepts or donates hydride directly to or from the enzyme substrate. In the second class, bound flavin (FAD or FMN) acts as an intermediate, either accepting a hydride from or donating a hydride to F_420_ (Shima *et al*. 2000; Ceh *et al*. 2009; Allegretti *et al*. 2014; Ahmed *et al*. 2015; Joseph *et al*. 2016; Oyugi *et al*. 2018). F_420_-dependent oxidoreductases of this second class often contain additional subunits with multiple iron-sulfur (FeS) clusters, which transfer electrons between the enzyme-substrate (i.e. H_2_ or formate) and F_420_, via FMN/FAD. In this role, the bound flavin acts as a modulator between the single-electron chemistry of the FeS clusters and the hydride chemistry of F_420_ (Wood, Haydock and Leigh 2003; Seedorf *et al*. 2007; Vitt *et al*. 2014).

**Figure 3.**
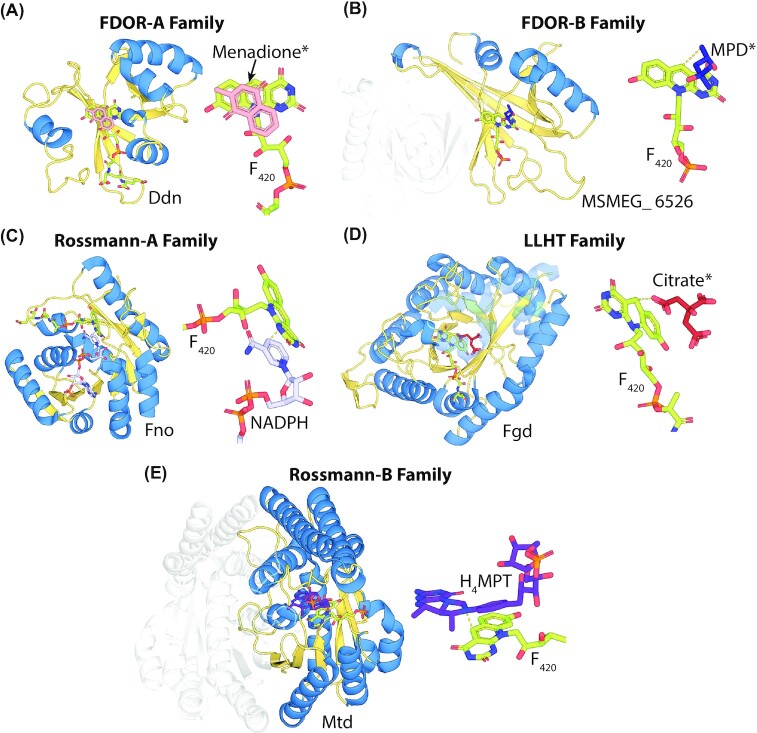
**F**_420_-dependent enzyme families that reduce or oxidize substrates via direct hydride transfer. Representative structures are shown of families of F_420_-dependent oxidoreductases in complex with F_420_ and substrate, inhibitor, or substrate analog. Inhibitors or substrate analogs are indicated with *. The secondary structural elements are highlighted (blue = α-helix, yellow = β-sheet or coil). **(A)** FDOR-A family F_420_H_2_-dependent menaquinone reductase (Ddn) from *M. tuberculosis* docked with menadione (PDB ID = 3R5R; Cellitti *et al*. 2012). **(B)** FDOR-B family enzyme of unknown function MSMEG_6526 from *M. smegmatis* in complex with 2-methyl-2,4-pentanediol (MPD; PDB ID = 4ZKY; Ahmed *et al*. 2015). **(C)** Rossmann-A fold enzyme NADPH:F_420_ oxidoreductase (Fno) from *A. fulgidus* in complex with NADPH (PDB ID = 1JAY; Warkentin *et al*. 2001). **(D)** LLHT family F_420_-reducing glucose-6-phosphate dehydrogenase (Fgd) from *M. tuberculosis* in complex with citrate (PDB ID = 3B4Y; Bashiri *et al*. 2008). The region of protein capping the active site is rendered transparent for clarity. **(E)** Rossmann-B fold enzyme F_420_-dependent CH_2_=H_4_MPT dehydrogenase (Mtd) from *Methanopyrus kandleri* in complex with CH_2_=H_4_MPT (PDB ID = 3IQE; Ceh *et al*. 2009).

**Figure 4.**
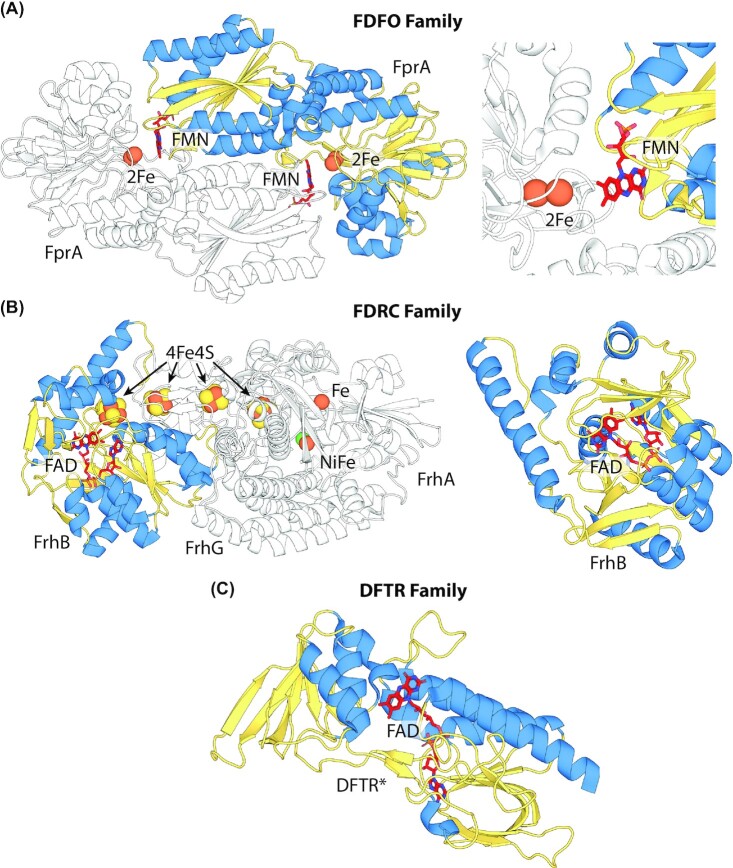
F_420_-dependent enzymes that mediate oxidation or reduction indirectly via flavin. Representative structures or models of families F_420_-dependent oxidoreductases that mediate hydride transfer via a bound flavin cofactor. Structures generated via homology modeling using Phyre2 (Kelley *et al*. 2015) are indicated with *. The secondary structural elements are highlighted (blue = α-helix, yellow = β-sheet or coil), FMN or FAD colored red and FeS clusters and metal ions are shown as spheres. **(A)** FDFO family F_420_H_2_-dependent flavodiiron oxidase (FprA) from *Methanothermobacter thermautotrophicus* responsible for the reduction of O_2_ to H_2_O (PDB ID = 2OHJ; Seedorf *et al*. 2007). **(B)** FDRC domain-containing F_420_-reducing NiFe hydrogenase (Frh) from *Methanothermobacter marburgensis* (PDB ID = 4CI0; Allegretti *et al*. 2014). **(C)** F_420_H_2_-dependent thioredoxin reductase (DFTR) *from M. jannaschii* (homology model; Susanti, Loganathan and Mukhopadhyay 2016).

F_420_-dependent enzymes are structurally diverse and can be classified into several families, which possess distinct folds and evolutionary histories (Greening *et al*. 2016). These families are often distributed in both F_420_-producing archaea and bacteria (Ney *et al*. 2017a) and have evolved to utilize F_420_ as a cofactor independently (Ahmed *et al*. 2015; Mascotti *et al*. 2018; Mascotti, Ayub and Fraaije 2020). F_420_-dependent enzyme families often include both F_420_-reducing and F_420_H_2_-oxidizing enzymes and are members of broader groups of oxidoreductases that utilize FMN, FAD, NAD(P)H, or heme as cofactors (Ahmed *et al*. 2015; Mascotti *et al*. 2018). Some of these groups contain multiple distinct lineages of enzymes that utilize F_420_, indicating that specificity for the cofactor arose on multiple occasions (Ahmed *et al*. 2015; Mascotti, Ayub and Fraaije 2020). The currently identified enzyme families that utilize F_420_ as a cofactor are summarized in Table 1, with functionally characterized F_420_-dependent dehydrogenases and F_420_H_2_-dependent reductases cataloged in Tables 2 and 3 respectively. The structures of representative examples of each family are shown in Figs 3 and 4. We have previously comprehensively reviewed the structure and function of these enzymes (Greening *et al*. 2016), and so we will not detail these aspects here.

**Table 1.** F_420_-dependent enzyme families.

F_420_-dependent protein family	Acronym	Protein fold	Mechanism of hydride transfer	Phylogenetic distribution	Characterized function(s)	Key references
Flavin/deazaflavin oxidoreductase	FDOR-A, FDOR-B	Split β-barrel	Direct F_420-_substrate	Actinobacteria, Chloroflexi	F_420_H_2-_dependent reduction of diverse substrates (e.g. menaquinone, tetracycline and biliverdin) with promiscuous activity often observed	Cellitti *et al*. (2012); Lapalikar *et al*. (2012); Ahmed *et al*. (2015); Greening *et al*. (2017)
Luciferase-like hydride transferase	LLHT	TIM-Barrel	Direct F_420_-substrate	Broadly found in F_420_ producing bacteria and archaea	F_420_-dependent oxidation or F_420_H_2_-dependent reduction of diverse substrates (e.g. G6P, mycolic acids and CH_2_=H_4_MPT)	Aufhammer *et al*.(2004, 2005); Bashiri *et al*. (2008); Nguyen *et al*. (2017); Mascotti *et al*. (2018)
F_420_-dependent NADPH oxidoreductase	Fno	Rossmann fold	Direct F_420_-substrate	Broadly found in F_420_ producing bacteria and archaea	Hydride transfer between F_420_/F_420_H_2_ and NADP/NADPH	Berk and Thauer (1997); Warkentin *et al*. (2001); Le *et al*. (2015); Joseph *et al*. (2016); Kumar *et al*. (2017)
F_420_-dependent H_4_MPT oxidoreductase	Mtd	Rossmann fold	Direct F_420_-substrate	Euryarchaeota: methanogens, ANME, Archaeoglobales	Hydride transfer between F_420_/F_420_H_2_ and CH≡H_4_MPT/CH_2_=H_4_MPT	Shima *et al*. (2000); Hagemeier *et al*. (2003a); Warkentin *et al*. (2005); Ceh *et al*. (2009)
F_420_H_2_-dependent flavodiiron oxidase	FprA	β-lactamase/flavodoxin	Indirect F_420_-flavin-2Fe-O_2_	Methanogenic archaea	Reduction of dioxygen (O_2_) to water (2 H_2_O) to detoxify O_2_	Seedorf *et al*. (2004,2007)
F_420_-dependent redox coupling oxidoreductase	FDRC	α/β/α-sandwich fold	Indirect F_420_-flavin-FeS-substrate	Euryarchaeota: methanogens, ANME, Archaeoglobales	Couples the reduction/oxidation of F_420_/F_420_H_2_ to that of formate, H_2_, methanophenazine, quinone or sulfite, via association with structurally diverse protein subunits.	Baron and Ferry (1989); Bäumer *et al*. (2000); Brüggemann, Falinski and Deppenmeier (2000); Johnson and Mukhopadhyay (2005,2008b); Welte and Deppenmeier (2011a); Allegretti *et al*. (2014); Vitt *et al*. (2014)
Deazaflavin-dependent thioredoxin reductase	DFTR	Thioredoxin reductase fold	Indirect F_420_-flavin-disulfide	Euryarchaeota: Methanococcales	Couples F_420_H_2_ oxidation to the reduction of thioredoxin	Susanti, Loganathan and Mukhopadhyay (2016)
F_420_-dependent bifurcating reductase	HdrA2	HdrA-like fold	Indirect F_420_-flavin-FeS-substrate	Euryarchaeota: Methanosarcinales	Couples F_420_H_2_ oxidation to the reduction of CoM-S-S-CoB and ferredoxin via bifurcation	Yan, Wang and Ferry (2017)

**Table 2.** Functionally characterized F_420_-reducing dehydrogenases. This table updates and expands upon the enzymes previously summarized and reviewed by Greening *et al*. (2016).

Oxidoreductase and domain	Physiological role	Taxonomic distribution	Family	EC no.	PDB ID	References
*Archaea*						
Frh: F_420_-reducing hydrogenase	Methanogenic growth on H_2_. Couples oxidation of H_2_ to the reduction of F_420_. May be physiologically reversible.	All orders of methanogens	FDRC	1.12.98.1	4OMF, 4CI0, 3ZFS, 6QGT	Tzeng, Wolfe and Bryant (1975); Jacobson *et al*. (1982); Muth, Morschel and Klein (1987); Kulkarni *et al*. (2009); Mills *et al*. (2013); Allegretti *et al*. (2014); Vitt *et al*. (2014); Ilina *et al*. (2019)
Ffd: F_420_-reducing formate dehydrogenase	Methanogenic growth on formate. Couples oxidation of formate to the reduction of F_420_. May be part of electron-bifurcating complex.	Many Euryarchaeota (e.g. Methanobacteriales, Methanococcales, Methanopyrales, Methanomicrobiales and Methanocellales)	FDRC	1.2.99.9		Jones and Stadtman (1981); Schauer and Ferry (1986); Costa *et al*. (2010); Tzeng, Wolfe and Bryant (1975); Wood, Haydock and Leigh (2003)
Adf: F_420_-reducing secondary alcohol dehydrogenase	Growth on secondary alcohols. Couples oxidation of secondary alcohols (e.g. isopropanol) to the reduction of F_420_.	Some Euryarchaeota *(*Methanomicrobiales and Methanocellales)	LLHT	1.1.98.5	1RHC	Widdel and Wolfe (1989); Bleicher and Winter (1991); Aufhammer *et al*. (2004); Martin *et al*. (2020)
*Bacteria*						
Fno: F_420_-reducing NADPH dehydrogenase	Exchanges electrons between NADP and F_420_. F_420_ reduction direction dominant in bacteria, as F_420_ is the secondary cofactor.	Many Actinomycetales (e.g. *Streptomyces*, *Thermobifida*, *Rhodococcus*, *Nocardia*and *Nocardioides*), Chloroflexi?, Alphaproteobacteria?, Betaproteobacteria?	Fno	1.5.1.40	5N2I	Eker, Hessels and Meerwaldt (1989); Heiss *et al*. (2002); Kumar *et al*. (2017)
Fgd: F_420_-reducing glucose-6-phosphate dehydrogenase	Heterotrophic growth. Couples oxidation of glucose-6-phosphate to the reduction of F_420_ via the pentose phosphate pathway.	Many Actinomycetales (e.g. *Mycobacterium, Actinoplanes, Microbacterium*and*Amycolatopsis*), Chloroflexi, Alphaproteobacteria?, Thaumarchaeota?	LLHT	1.1.98.2	3B4Y	Bashiri *et al*. (2008); Oyugi *et al*. (2018)
Fsd: F_420_-reducing sugar-6-phosphate dehydrogenase	Heterotrophic growth. Couples oxidation of glucose-, fructose- or mannose-6-phosphate to the reduction of F_420_. Similar to Fgd, with a catalytic preference for glucose-6-phosphate, but an expanded substrate specificity.	Some Actinomycetales (e.g. *Nocardioides* and *Cryptosporangium*)	LLHT	1.1.98.2		Mascotti *et al*. (2018)
fHMAD: F_420_-reducing hydroxymycolic acid dehydrogenase	Cell wall biosynthesis. Catalyzes F_420_-dependent oxidation of hydroxymycolic acids to ketomycolic acids.	Few *Mycobacterium* (primarily pathogenic species)	LLHT			Bashiri *et al*. (2012); Purwantini and Mukhopadhyay (2013)
Amm4: F_420_-dependent ammosamide dehydrogenase	Putative dehydrogenase involved in primary amide formation in the pyrroloquinoline alkaloid ammosamide. Details of reaction mediated and the product formed are unresolved.	Few Actinomycetales (e.g. *Streptomyces* and *Amycolatopsis*)	FDOR-B			Jordan and Moore (2016)

**Table 3.** Functionally characterized F_420_H_2_-dependent reductases. This table updates and expands upon the enzymes previously summarized and reviewed by Greening *et al*. (2016).

Oxidoreductase and domain	Physiological role	Taxonomic distribution	Family	EC no.	PDB ID	References
*Archaea*						
Mtd: F_420_-reducing methylene-H_4_MPT dehydrogenase	Reduces CH≡H_4_MPT to CH_2_=H_4_MPT with F_420_H_2_ during CO_2_-reducing methanogenesis. Performs the opposite reaction during methylotrophic methanogenesis and anaerobic methane/alkane oxidation.	Various Euryarchaeota including: all orders of methanogens, Archaeoglobales, ANME and Halobacteriales; various TACK and Asgard archaea	Mtd	1.5.98.1	1QV9, 1U6I, 3IQF, 3IQE	Hartzell *et al*. (1985); Te Brömmelstroet *et al*. (1991a,b); Hagemeier *et al*. (2003a,b); Ceh *et al*. (2009)
Mer: F_420_H_2_-dependent methylene-H_4_MPT reductase	Reduces CH_2_=H_4_MPT to CH_3_-H_4_MPT with F_420_H_2_ during CO_2_-reducing methanogenesis. Performs the opposite reaction during methylotrophic methanogenesis and anaerobic methane/alkane oxidation.	Various Euryarchaeota including: all orders of methanogens, Archaeoglobales, ANME and Halobacteriales; various TACK and Asgard archaea	LLHT	1.5.98.2	1F07, 1EZW, 1Z69	Te Brömmelstroet *et al*. (1991b); Shima *et al*. (2000); Aufhammer *et al*. (2005); Ceh *et al*. (2009)
Fpo: F_420_H_2_-dependent methanophenazine reductase	Proton-translocating primary dehydrogenase in respiratory chain transferring electrons from F_420_H_2_ to heterodisulfide.	Methanosarcinales	FDRC	1.1.98.4		Bäumer *et al*. (1998); Deppenmeier, Lienard and Gottschalk (1999); Ide, Bäumer and Deppenmeier (1999); Bäumer *et al*. (2000); Welte and Deppenmeier (2011a)
Fqo: F_420_H_2_-dependent quinone reductase	Proton-translocating primary dehydrogenase in respiratory chain transferring electrons from F_420_H_2_ to sulfate.	Archaeoglobales and ANME	FDRC	1.1.98.4		KUNOW *et al*. (1994); Brüggemann, Falinski and Deppenmeier (2000); Hallam *et al*. (2004); Hocking *et al*. (2014)
Fpr: F_420_H_2_-dependent oxidase	Detoxifies O_2_ by mediating the four-electron reduction of O_2_ to H_2_O with F_420_H_2._	Methanobacteriales, Methanococcales, Methanomicrobiales and Methanocellales	FprA	1.5.3.22	2OHH, 2OHI, 2OHJ	Seedorf *et al*. (2004,2007)
Fsr: F_420_H_2_-dependent sulfite reductase	Detoxifies sulfite by mediating the six electron reduction of sulfite to sulfide with F_420_H_2_. Also enables the use of sulfite as an S source.	Methanobacteriales and Methanococcales	FDRC	1.8.98.3		Johnson and Mukhopadhyay (2005, 2008a)
Fno: F_420_H_2_-dependent NADP^+^ reductase	Exchanges electrons between NADP and F_420_. NADP^+^ reduction direction dominant in archaea, as NADP is the secondary cofactor.	Various Euryarchaeota including: all orders of methanogens, Archaeoglobales and ANME; various TACK and Asgard archaea	Fno	1.5.1.40	1JAY, 1JAX	Tzeng, Wolfe and Bryant (1975); Kunow *et al*. (1993); Berk and Thauer (1997); Warkentin *et al*. (2001)
HdrA2B2C2: F_420_H_2_-dependent, electron-bifurcating, heterodisulfide reductase	The HdrA2 subunit of this complex oxidizes F_420_H_2,_ with subunits HdrB2 and HdrC2 bifurcating the resulting electrons to ferredoxin and CoM-S-S-CoB (heterodisulfide). Thought to mediate energy conservation during acetoclastic methanogenesis.	Methanosarcinales	HdrA2			Yan, Wang and Ferry (2017)
DFTR: F_420_H_2_-dependent thioredoxin reductase	Recycling of the thioredoxin disulfide through reduction by electrons transferred from F_420_H_2,_ via a low potential FMN and disulfide redox center.	Methanococcales	DFTR	1.8.1.9		Susanti, Loganathan and Mukhopadhyay (2016)
*Bacteria*						
Ddn: F_420_H_2_-dependent menaquinone reductase	Reduction of the respiratory cofactor menaquinone for energy conservation and possibly to mitigate redox stress. Also catalyzes the promiscuous activation nitroimidazole prodrugs. FDOR-A1 family.	Most Actinomycetales (e.g., *Mycobacterium, Streptomyces, Rhodococcu*s), Chloroflexi?, Methanosarcinales?	FDOR-A	1.1.98.-	3H96, 4Y9I, 3R5R, 3R57	Taylor *et al*. (2010); Cellitti *et al*. (2012); Gurumurthy *et al*. (2013); Ahmed *et al*. (2015); Lee *et al*. (2020)
Fbr: F_420_H_2_-dependent biliverdin reductase	Reduction of the heme degradation product biliverdin to bilirubin. May also reduce mycobillins. FDOR-B3 and FDOR-B4 family.	Most Actinomycetales (e.g., *Mycobacterium, Streptomyces, Rhodococcus*), Chloroflexi?	FDOR-B		2ASF, 4QVB, 1W9A	Canaan *et al*. (2005); Biswal *et al*. (2006); Ahmed *et al*. (2015, 2016); Mashalidis *et al*. (2015)
Fts: F_420_H_2_-dependent tetracycline synthase	Reduction of dehydrotetracyclines to tetracyclines during streptomycete antibiotic synthesis. Role in mycobacteria unknown. FDOR-B1 family.	Most Actinomycetales (e.g., *Mycobacterium, Streptomyces*and*Rhodococcus*), Chloroflexi?, Thaumarchaeota?	FDOR-B			Taylor *et al*. (2010); Wang *et al*. (2013); Ahmed *et al*. (2015)
TpnL: F_420_H_2_–dependent dehydropiperidine reductase	Reduction of the dehydropiperidine moiety to piperidine during the synthesis of thiopeptins antibiotics. FDOR-B family.	Some Actinomycetales (*Streptomyces*, *Amycolatopsis*, *Micromonospora* and *Actinoalloteichus*)	FDOR-B			Ichikawa, Bashiri and Kelly (2018)
GupA: F_420_H_2_–dependent dihydropyrazine reductase	Reduction of the dihydropyrazine ring to piperzine during the synthesis of guanipiperazines. FDOR-B family.	Some Actinomycetales (*Streptomyces*)	FDOR-B			Shi *et al*. (2021)
Other F_420_H_2_-dependent flavin/deazaflavin oxidoreductases (FDORs)	Physiological substrates of A2-A4, B1, B2, B5, B6, AA1- AA5 families unknown. Promiscuous reductase activity observed towards multiple chemical classes that may facilitate detoxification. AA1s may be fatty acid saturases.	Most Actinomycetales (e.g., *Mycobacterium, Streptomyces* and *Rhodococcus*), Chloroflexi?, Halobacteriales?	FDOR-A/B		3F7E, 1RFE, 4ZKY	Lapalikar *et al*. (2012); Ahmed *et al*. (2015); Jirapanjawat *et al*. (2016); Greening *et al*. (2017)
Fht: F_420_H_2_-dependent picrate reductase	Reduces 2,4,6-trinitrophenol (picrate) for use as a C and N source through hydride transfer to the nitroaromatic ring.	Few Actinomycetales (*Rhodococcus*, *Nocardia*, *Nocardioides*)	LLHT			Ebert, Rieger and Knackmuss (1999); Heiss *et al*. (2002)
Fps/Adp6: F_420_H_2_-dependent 4-alkyl-L-proline derivative reductases	Reduction of 4-alkyl-L-proline derivatives (APDs) in the final step in the biosynthesis of this compound. Different enzymes of this class impart structural diversity by reducing either the endocyclic imine or the exocyclic double bond of APDs.	Some Actinomycetales (*Streptomyces*, *Micrococcus* and *Streptosporangium*)	LLHT			Li *et al*. (2009a,b); Steiningerova *et al*. (2020)
fPKR: F_420_H_2_-dependent phthiodiolone ketoreductase	Reduction of phthiodiolone keto intermediates during the synthesis of phthiocerol dimycocerosates (PDIM), a class of mycobacterial cell surface apolar lipids.	Few *Mycobacterium* (primarily pathogenic species)	LLHT			Purwantini, Daniels and Mukhopadhyay (2016)
LxmJ: F_420_H_2_-dependent 2,3-didehydroalanine reductase	Stereospecific reduction of the 2,3-didehydroalanine reductase to D-alanine during class V lanthipeptide biosynthesis	Few *Streptomyces*	LLHT			Tao *et al*. (2020)
Other H_2_-dependent luciferase-like hydride transferases (LLHTs)	Unknown. Likely to have diverse roles in endogenous and exogenous redox metabolism of organic compounds.	Most Actinomycetales (e.g., *Mycobacterium, Streptomyces*and*Rhodococcus*)	LLHT			

### Taxonomic distribution

**Figure 5.**
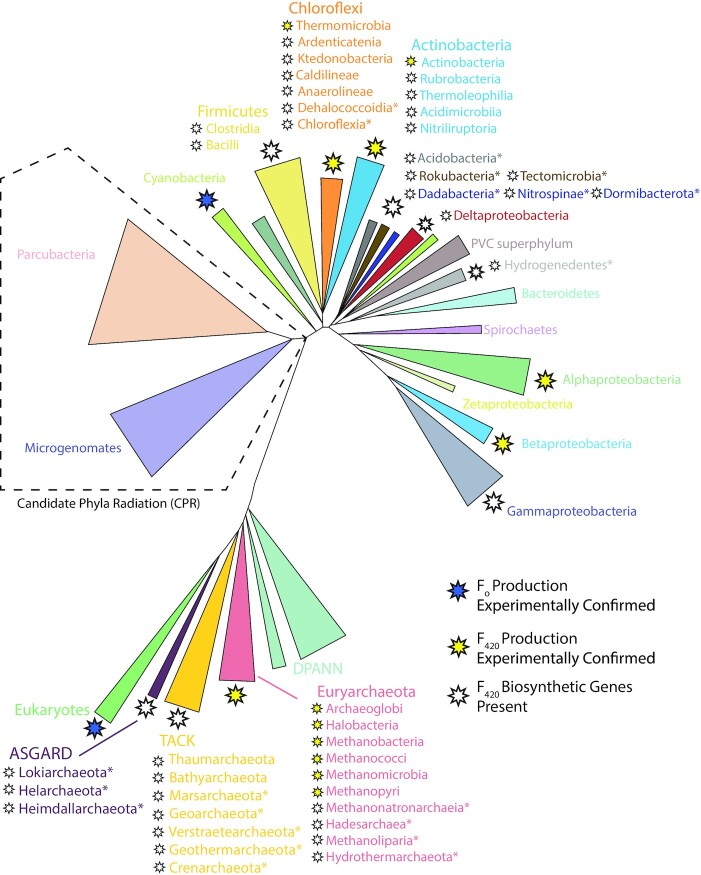
Phylogenetic distribution of F_O_ and F_420_ producing organisms. A simplified two-domain tree of life depicted the organisms shown or predicted to produce the 5-deazaflavins F_O_ or F_420_. This is based on currently available data from published work (Greening *et al*. 2016; Ney *et al*. 2017a), and genomic and metagenomic data in the NCBI database (as of October 2020). Tree topography is based on Hug et. al. (Hug *et al*. 2016) and Castelle and Banfield (2018), with additional reference to Zhou *et al*. (2020), Wang *et al*. (2019) and Momper, Aronson and Amend (2018). * = F_420_ biosynthesis genes detected only in multiple metagenome-assembled genomes (MAGs) or single-amplified genomes (SAGs) from these archaea and bacteria, rather than genomes derived from pure culture.

Until recently F_420_ was thought to be a rare cofactor, taxonomically restricted to the members of archaeal phylum Euryarchaeota and the bacterial phylum Actinobacteria (Ney *et al*. 2017a). However, recent studies applying genomic, spectroscopic and biochemical analysis have demonstrated that F_420_ is much more widely distributed among bacteria and archaea than previously thought (Kerou *et al*. 2016; Lackner *et al*. 2017; Ney *et al*. 2017a; Braga *et al*. 2019, 2020). Prior to these studies, it was assumed that F_O_ production was more widespread than F_420_. Yet genomic analysis shows that, in the majority of organisms, the genes required for F_O_ biosynthesis co-occur with those required for its conversion to F_420_, indicating that F_O_ is generally produced as a precursor for F_420_ biosynthesis, with a possible secondary role as a chromophore in some F_420_ producers (Kiontke *et al*. 2014; Ney *et al*. 2017a). A phylogenetic tree and accompanying table outlining microbial lineages biochemically demonstrated to produce F_O_ and F_420_, as well as those predicted to produce these cofactors based on genomic data, is presented in Fig. 5 and Table 4. There is currently no evidence that F_420_ is synthesized as a redox cofactor by eukaryotes. The distribution of F_420_ biosynthesis genes among bacteria and archaea appears to be widespread in some lineages (i.e. Euryarchaeota and Actinobacteria; Cheeseman, Toms-Wood and Wolfe 1972; Eirich, Vogels and Wolfe 1979; Lin and White 1986; Bair, Isabelle and Daniels 2001), but variable among others (i.e. TACK lineages of Archaea and Proteobacteria; Kerou *et al*. 2016; Ney *et al*. 2017a). F_420_ biosynthesis genes are highly abundant in metagenomes from diverse soil, marine and some host-associated ecosystems, further indicating that F_420_ biosynthesis is a widespread trait (Ney *et al*. 2017a).

**Table 4.** Confirmed and predicted F_420_-producing organisms. Experimentally confirmed F_420_ producers are highlighted in yellow, while predicted F_420_ producers with a full complement of F_420_ biosynthesis genes based on analysis of assembled pure culture genomes or multiple MAGs/SAGs, are highlighted in green.

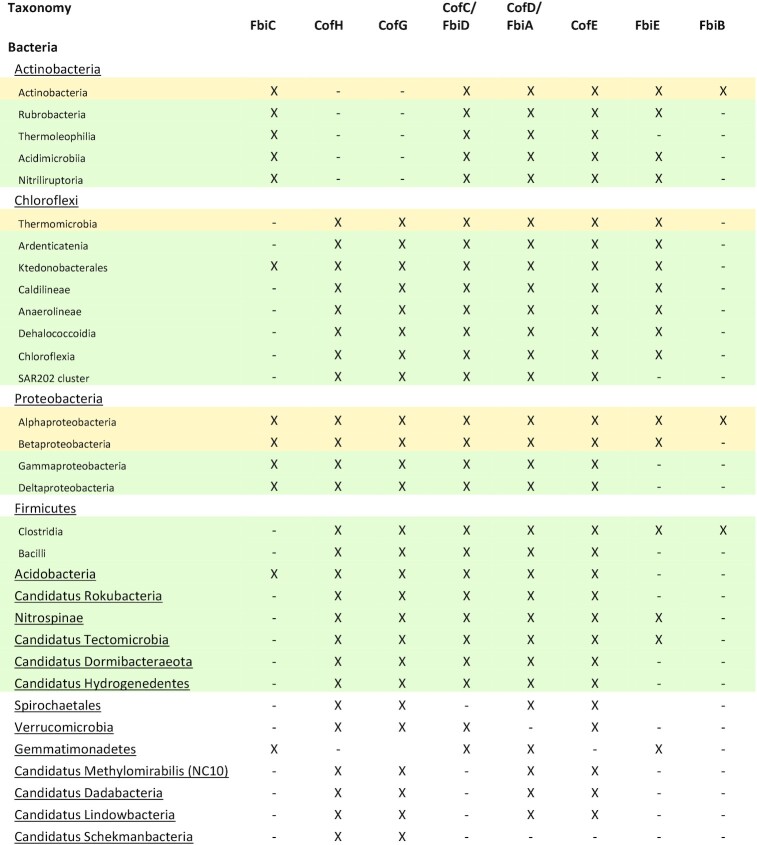
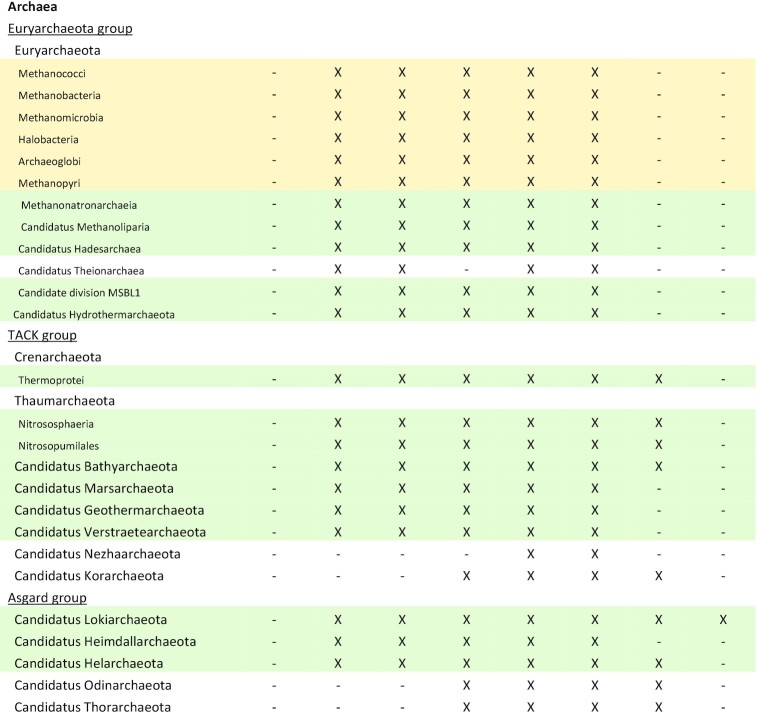

**Table 5.** Purified F_420_H_2_-dependent reductases that have been explored for substrate specificity and range.

									*In vitro* activity					
Enzyme nme	Originating organism	Sequence ID	Physiological substrate	Enzyme class	PDB ID	Quinones	Coumarins	Enones	Enals	Pyrones	Pyrans	Triarylmethanes	Secondary alcohols	Refs
MSMEG_5998	*M. smegmatis*	ABK71916	Menaquinone	FDOR-A1		++++	+++	+	ND	-	-	++	ND	Greening *et al*. (2017)
MSMEG_2027	*M. smegmatis*	ABK75334	Menaquinone	FDOR-A1	4Y9I	+++	+	+	ND	++	-	++	ND	Greening *et al*. (2017)
MSMEG_2850	*M. smegmatis*	AWT53773		FDOR-A1		++++	+++	+	ND	-	-	++	ND	Greening *et al*. (2017)
MSMEG_3356	*M. smegmatis*	ABK75759		FDOR-A1	3H96	ND	++	ND	ND	ND	ND	ND	ND	Taylor *et al*. (2010)
MSMEG_3004	*M. smegmatis*	ABK74167		FDOR-A1		ND	++	ND	ND	ND	ND	ND	ND	Taylor *et al*. (2010); Lapalikar *et al*. (2012)
MSMEG_5030	*M. smegmatis*	ABK74375		FDOR-A2		+++	+	-	ND	+	+	++	ND	Greening *et al*. (2017)
MSMEG_3380	*M. smegmatis*	ABK72884		FDOR-B1	3F7E	+++	++	+	ND	-	-	++	ND	Greening *et al*. (2017)
MSMEG_0048	*M. smegmatis*	ABK73917		FDOR-B1		++	+	+	ND	+	-	+	ND	Greening *et al*. (2017)
MSMEG_6325	*M. smegmatis*	ABK73368		FDOR-A3		+	+	++	ND	-	-	++	ND	Greening *et al*. (2017)
MSMEG_5170	*M. smegmatis*	ABK72943		FDOR-B3		+++	+	-	ND	-	-	+	ND	Greening *et al*. (2017)
MSMEG_6848	*M. smegmatis*	ABK75254		LPOR-like/FDOR-B1		+++	+	-	ND	+	-	+	ND	(Greening *et al*. 2017)
MSMEG_6526	*M. smegmatis*	ABK76173		FDOR-B2	5JV4, 4ZKY	+	-	-	ND	-	-	+	ND	Greening *et al*. (2017)
MSMEG_3880	*M. smegmatis*	ABK75472	Biliverdin	FDOR-B4		+	-	-	ND	-	-	+	ND	Greening *et al*. (2017)
MSMEG_5717	*M. smegmatis*	ABK72164		FDOR-B		ND	-	ND	ND	ND	ND	ND	ND	Greening *et al*. (2017)
FDR-Rh1	*Rhodococcus jostii*	ABG96463		FDOR-A		+++	ND	+	+++	ND	ND	ND	ND	Mathew *et al*. (2018)
FDR-Rh2	*Rhodococcus jostii*	ABG97172		FDOR-A		+++	ND	+	++	ND	ND	ND	ND	Mathew *et al*. (2018)
FDR-Mha	*Mycobacterium hassicum*	WP_005623184		FDOR-A		+++	ND	+	++	ND	ND	ND	ND	Mathew *et al*. (2018)
Adf	*M. thermophilicus*	CAA77275		LLHT	1RHC	ND	ND	ND	ND	ND	ND	ND	+++	Martin *et al*. (2020)

### F_420_ production and roles within archaea

Within archaea, F_420_ production has only been biochemically confirmed in Euryarchaeota and much of our understanding of the physiological roles of the cofactor is derived from these organisms (Jacobson and Walsh 1984; Schmitz *et al*. 1991; Vaupel and Thauer 1995; Berk and Thauer 1997; Thauer 1998; Brüggemann, Falinski and Deppenmeier 2000). Currently, available genomic and metagenomic datasets show that a complete complement of genes necessary for F_420_ biosynthesis is also present in members of two other major archaeal groups, the TACK and Asgard archaea (Kerou *et al*. 2016; Ney *et al*. 2017a; Jay *et al*. 2018; Spang *et al*. 2019). The majority of these putative F_420_-producing archaea remain uncultured, with most detected through metagenome-assembled genomes (MAGs) and single-amplified genomes (SAGs; Spang, Caceres and Ettema 2017; Williams *et al*. 2017; Ney *et al*. 2017a; Jay *et al*. 2018). Genomes assembled by these methods often exhibit low coverage and completeness and suffer from sampling bias due to their often low relative abundance in the community (Albertsen *et al*. 2013). As such, the current list of F_420_ producing archaea compiled for this review, and shown in Table 4, is an underestimation of the actual distribution of the cofactor. Growing evidence indicates that F_420_-dependent redox metabolism of one-carbon units is widespread in archaea, enabling the processes of methanogenesis, acetogenesis and alkane oxidation (Laso-Pérez *et al*. 2016; Adam, Borrel and Gribaldo 2019; Evans *et al*. 2019; Orsi *et al*. 2020). The central role of F_420_ in this pathway likely goes some way to explain its widespread production by the archaeal domain. However, the role of F_420_ goes well beyond one-carbon metabolism and the diversity of F_420_-producing archaea indicates that many additional functions likely remain to be discovered (Kozubal *et al*. 2013; Kerou *et al*. 2016; Susanti, Loganathan and Mukhopadhyay 2016; Ney *et al*. 2017a; Jay *et al*. 2018).

#### Roles in Euryarchaeota

##### Methanogenic Euryarchaeota

Methanogens are a diverse group of obligately anaerobic archaea that produce methane as the end product of their energy generation pathways (Liu and Whitman 2008). Methanogens encompass at least seven orders within the Euryarchaeota (Brochier, Forterre and Gribaldo 2004; Bapteste, Brochier and Boucher 2005; Brochier-Armanet, Forterre and Gribaldo 2011; Borrel *et al*. 2013; Evans *et al*. 2019), though genome-resolved metagenomics indicates there are potentially methanogenic archaea outside this phylum (Vanwonterghem *et al*. 2016; Sorokin *et al*. 2017; Spang and Ettema 2017; Berghuis *et al*. 2019). All cultured methanogens synthesize F_420_, which serves as a central redox cofactor for two of the three major routes of methanogenesis: the CO_2_-reducing and methylotrophic pathways (Cheeseman, Toms-Wood and Wolfe 1972; Edwards and McBride 1975; Doddema and Vogels 1978; Eirich, Vogels and Wolfe 1979; van Beelen, Dijkstra and Vogels 1983; Dolfing and Mulder 1985). As such, it is often present at high concentrations (up to 2.0 µmol per g dry weight) in these methanogens (Eirich, Vogels and Wolfe 1979; Isabelle, Simpson and Daniels 2002).

**Figure 6.**
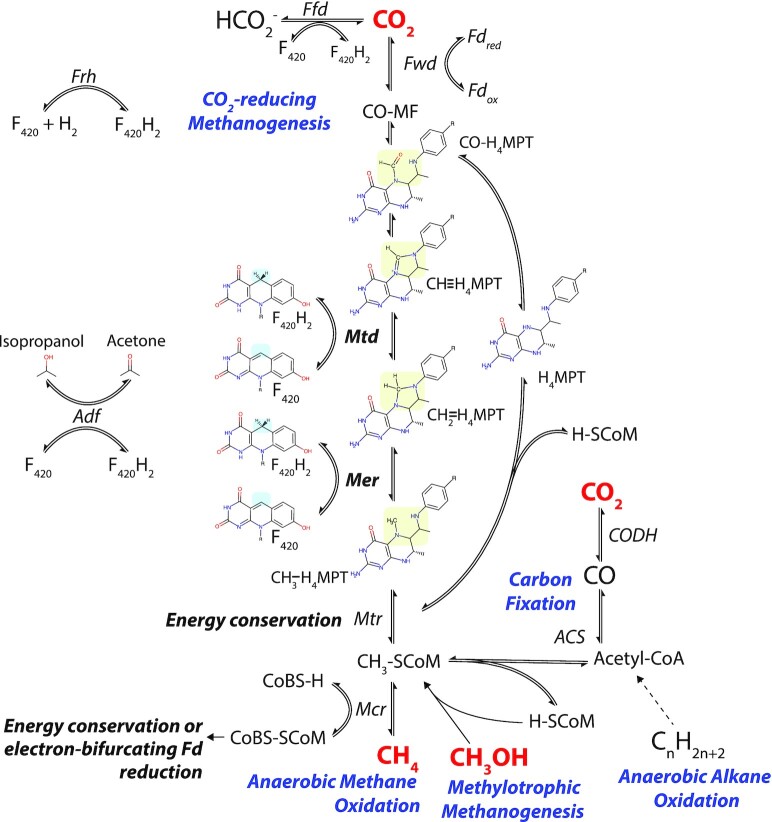
F_420_-dependent reactions of one-carbon metabolism in archaea. F_420_ is a cofactor involved in key steps in hydrogenotrophic methanogenesis, methylotrophic methanogenesis, anaerobic methanotrophy and anaerobic alkane oxidation in archaea. Hydride transfer reactions involving F_420_-dependent enzymes are indicated as is the enzyme responsible. F_420_H_2_ reduced through the oxidation of formate (Ffd), H_2_ (Frh), or secondary alcohols (Adf) can be utilized for reactions mediated by Mtd, Mer, or for other cellular processes. Only reactions mediated by F_420_-dependent enzymes are shown in detail. For a full outline of methanogenesis pathways, refer to the following reviews on the subject (Deppenmeier 2002; Thauer *et al*. 2008; Timmers *et al*. 2017; Evans *et al*. 2019).

In the CO_2_-reducing pathway, CO_2_ is progressively reduced to methane using electrons derived from exogenous substrates such as H_2_, formate and less commonly secondary alcohols (Tzeng, Bryant and Wolfe 1975; Widdel and Wolfe 1989; Fig. 6). F_420_H_2_ donates a hydride for two of these reaction steps, after first being reduced by F_420_-reducing dehydrogenases utilizing H_2_ (Frh; Jacobson *et al*. 1982; Muth, Morschel and Klein 1987; Fiebig and Friedrich 1989; de Poorter, Geerts and Keltjens 2005; Allegretti *et al*. 2014), formate (Ffd; Schauer and Ferry 1986; Shuber *et al*. 1986; Baron and Ferry 1989), or secondary alcohols (Adf; Widdel and Wolfe 1989; Bleicher and Winter 1991). In this pathway, CO_2_ is first condensed with the cofactor methanofuran, before being transferred to tetrahydromethanopterin (H_4_MPT) to form 5-formyltetrahydromethanopterin (CHO-H_4_MPT). CHO-H_4_MPT then undergoes enzymatically mediated intramolecular condensation to form 5,10-methenyltetrahydromethanopterin (CH≡H_4_MPT; Thauer 2012), which is progressively reduced by F_420_H_2_ via methylene-H_4_MPT dehydrogenase (Mtd) to form 5,10-methylenetetrahydromethanopterin (CH_2_=H_4_MPT) and methylene-H_4_MPT reductase (Mer) to form N5-methyltetrahydromethanopterin (CH_3_-H_4_MPT; Hendrickson and Leigh 2008). The CO_2_-derived methyl group resulting from these reactions is then transferred to coenzyme M (CoM), before it is substituted by coenzyme B (CoB), forming the heterodisulfide CoB-S-S-CoM and releasing methane (Fig. 6; Thauer 1998). The methyl transfer from CH_3_-H_4_MPT to CoM is mediated by the MtrA-H membrane protein complex, which conserves energy through the pumping of sodium ions out of the cell (Welander and Metcalf 2005; Thauer *et al*. 2008). In addition to the MtrA-H complex, energy is also conserved through respiratory reduction of CoB-S-S-CoM in methanogens with cytochromes (i.e. *Methanosarcinales*) or by electron bifurcation in methanogens without cytochromes (Thauer *et al*. 2008; Kaster *et al*. 2011; Welte and Deppenmeier 2014). In the case of methanogens with cytochromes, F_420_H_2_ can serve as a direct electron donor to the respiratory chain; this depends on the activity of F_420_H_2_-dependent methanophenazine reductase (Fpo), a 14-subunit complex similar to bacterial complex I that directly pumps protons using the energy released from electron transfer from F_420_H_2_ to the membrane-diffusible cofactor methanophenazine (Deppenmeier *et al*. 1990; Abken and Deppenmeier 1997; Bäumer *et al*. 1998, 2000; Welte and Deppenmeier 2011b).

F_420_ has distinct roles in the methylotrophic and acetoclastic methanogenesis pathways. In the methylotrophic methanogenesis pathway, methyl groups (from methanol, methylated amines and methylated sulfides) are alternatively converted into CH_4_ (reductive route) and CO_2_ (oxidative route; Fig. 6; Krzycki *et al*. 1987; Ferry and Kastead 2007). The oxidative route likely occurs through the reverse direction of the CO_2_-reducing pathway, with the methyl group first transferred to CoM, then H_4_MPT, before being oxidized sequentially by Mer and Mtd, yielding F_420_H_2_. The reductive route proceeds from CH_3_-S-CoM in the same fashion as the CO_2_-reducing pathway (Fig. 6; Deppenmeier 2002; Thauer *et al*. 2008). Methanogens with cytochromes use F_420_H_2_ generated through the oxidative arm of the methylotrophic pathway as an input to the respiratory chain via Fpo (Welte and Deppenmeier 2011b). Acetocastic methanogenesis is F_420_-independent, producing CO_2_ and CH_4_ from acetate utilizing a largely distinct set of enzymes to the hydrogenotrophic or methylotrophic pathways (Smith and Mah 1978; Barber *et al*. 2011). However, despite not being required for this process, F_420_ is present in facultatively acetoclastic *Methanosarcina* when grown solely on acetate and in the obligately acetoclastic genus *Methanothrix*, indicating that the cofactor has roles in methanogen physiology beyond those of methanogenesis (Baresi and Wolfe 1981; Barber *et al*. 2011; Zhu *et al*. 2012). In support of this, a potential electron-bifurcating heterodisulfide reductase that uses ferredoxin and F_420_H_2_ as electron donors has been identified in *Methanosarcina acetivorans* (Yan, Wang and Ferry 2017).

Dedicated F_420_-dependent enzymes have been shown to mediate other diverse reactions in methanogens, as detailed in Tables 2 and 3. These include reduction of the redox cofactors NADP^+^ (F_420_H_2_-dependent NADP reductase; Fno) and thioredoxin (F_420_H_2_-dependent flavin-containing thioredoxin reductase; DFTR; Spaans *et al*. 2015; Susanti, Loganathan and Mukhopadhyay 2016), mobilization of sulfite as a sulfur source (F_420_H_2_-dependent sulfite reductase; Fsr; Johnson and Mukhopadhyay 2008a) and detoxification of atmospheric O_2_ (F_420_H_2_-dependent oxidase; FprA; Seedorf *et al*. 2007).

##### Methane-, ethane- and butane-oxidizing Euryarchaeota

Anaerobic methanotrophy is a biogeochemically significant process in which methane of biological or abiotic origin is oxidized to CO_2_, with nitrate, sulfate, or transition metal ions as terminal electron acceptors (Bhattarai, Cassarini and Lens 2019). Up to 90% of the methane produced by marine sediment is estimated to be internally recycled by this process, thereby moderating methane release into the atmosphere (Reeburgh 2007; Conrad 2009; Knittel and Boetius 2009). There is strong evidence that this process is mediated by uncultured methanotrophic Euryarchaeota (ANME; Haroon *et al*. 2013; Cai *et al*. 2018). These archaea form at least three phylogenetically distinct groups, which are closely related to Methanomicrobiales (ANME-1) and Methanosarcinales (ANME-2a/b/c and ANME-3; Wang *et al*. 2014). ANME have not been propagated in pure culture. However, genetic, transcriptomic and biochemical evidence indicates they oxidize methane using an F_420_-dependent pathway analogously to methylotrophic methanogenesis (Wang *et al*. 2014; Timmers *et al*. 2017). Metagenomic and metatranscriptomic analysis of the nitrate-reducing methanotroph *Methanoperedens nitroreducens* (part of the ANME-2 lineage) in an enriched bioreactor showed that it expresses a complete reverse methanogenesis pathway, including Mtd and Mer, as well as F_420_ biosynthesis genes and a putative respiratory F_420_H_2_-dependent quinone reductase (Fqo) complex (Arshad *et al*. 2015). Many ANME appear to perform methanotrophy syntrophically, forming associations with sulfate, nitrite, or nitrate-reducing bacteria, which likely explains the inability to isolate them in pure culture (Boetius *et al*. 2000; Beal, House and Orphan 2009; Haroon *et al*. 2013). Similar to anaerobic methane oxidizers, enrichment cultures of novel Euryarchaeota lineages have recently been shown to be capable of anaerobically oxidizing short-chain alkanes. Members of candidate genera Argoarchaeum and Syntrophoarchaeum are capable of anaerobically oxidizing ethane and butane respectively (Laso-Pérez *et al*. 2016; Chen *et al*. 2019). They are predicted to produce F_420_, and likely utilize the reverse methanogenesis pathway, combined with β-oxidation, to oxidize these compounds (Laso-Pérez *et al*. 2016).

##### Sulfate-reducing and halophilic Euryarchaeota

Archaeoglobi are a class of sulfate-reducing archaea that appear to have evolved from a methanogenic ancestor but have developed a non-methanogenic lifestyle (Stetter *et al*. 1987; Klenk *et al*. 1997). Archaeoglobi are primarily heterotrophic sulfate-reducing hyperthermophiles that inhabit deep-sea vents (Stetter *et al*. 1987; Nercessian *et al*. 2005). The well-characterized isolate *Archaeoglobus fulgidus* uses F_420_ as its central redox cofactor (Möller-Zinkhan, Börner and Thauer 1989; Gorris and Voet 1991). F_420_ is reduced through distinct routes depending on whether the growth substrate is H_2_/CO_2_ or lactate (Möller-Zinkhan and Thauer 1990). Lactate is converted to three molecules of CO_2_, through a process analogous to the oxidative methylotrophic pathway of methanogens, generating F_420_H_2_ via the action of Mer and Mtd (Schmitz *et al*. 1991; Schwörer *et al*. 1993). *A. fulgidus* does not possess the F_420_-reducing hydrogenase Frh, and it remains unresolved how it generates F_420_H_2_ during hydrogenotrophic growth; possible routes include electron transfer from reduced ferredoxin, quinols (via reverse electron transfer), or NADPH (via Fno; Möller-Zinkhan, Börner and Thauer 1989; Klenk *et al*. 1997; Hocking *et al*. 2014). F_420_H_2_ produced by substrate oxidation then donates electrons to a sulfate-reducing respiratory chain via the proton-translocating F_420_H_2_-dependent quinone reductase (Fqo; Kunow *et al*. 1994; Brüggemann, Falinski and Deppenmeier 2000). Outside of central metabolism, little is known about the role of F_420_ in Archaeoglobi. However, *A. fulgidus* possesses Fno, which is thought to be the sole route for NADP reduction (Kunow *et al*. 1993; Warkentin *et al*. 2001). F_420_ production has also been experimentally determined in the halophiles *Halobacterium* and *Halococcus*, though its physiological role remains undetermined (Lin and White 1986; De Wit and Eker 1987).

#### Roles in other Archaea

##### TACK lineages of Archaea

The TACK lineage represents a major grouping of archaea originally containing the phyla Thaumarchaeota, Aigarchaeota, Crenarchaeota and Korarchaeota, but now expanded to contain several other recently identified phyla (Guy and Ettema 2011; Spang, Caceres and Ettema 2017; Wang *et al*. 2019). Diverse members of the TACK group contain a full complement of genes for F_420_ (Spang *et al*. 2012; Zhalnina *et al*. 2014; Evans *et al*. 2015; Kerou *et al*. 2016; Vanwonterghem *et al*. 2016; Ney *et al*. 2017a; Jay *et al*. 2018; Berghuis *et al*. 2019; Table 4), though no definitive experimental evidence confirming the production and roles of F_420_ has been presented. Putative F_420_ producing species adopt diverse aerobic and anaerobic lifestyles (Jay *et al*. 2018; Yu *et al*. 2018; Berghuis *et al*. 2019). F_420_ production appears to be a common trait in Thaumarchaeota (Tourna *et al*. 2011; Kozlowski *et al*. 2016; Ren *et al*. 2019; Reji and Francis 2020), including ammonium-oxidizing archaea (AOA) that mediate nitrification in soil and marine ecosystems (Kuypers, Marchant and Kartal 2018). Genomic analysis and fluorescence microscopy indicate both *Nitrososphaera gargensis* and *Nitrososphaera viennensis* synthesize F_420_ in significant quantities (Spang *et al*. 2012; Kerou *et al*. 2016), though the presence and role of F_420_ in this phylum has not been biochemically confirmed. Given *Nitrososphaera* are aerobes that cannot perform methanogenesis, the cofactor is unlikely to play a role in one-carbon transformations (Kerou *et al*. 2016; Ren *et al*. 2019). Proteomic analysis indicates that Fno and putative F_420_-dependent oxidoreductases of the luciferase-like hydride transferase (LLHT) and flavin/deazaflavin oxidoreductase (FDOR) families are produced at high levels, suggesting a role for the cofactor in biosynthetic or biodegradative processes (Kerou *et al*. 2016).

Several other TACK phyla also encode F_420_ biosynthesis genes. Marsarchaeota and Geoarchaeota, two closely related aerobic chemoheterotrophic phyla recently discovered in thermophilic iron-rich microbial mats, also encode F_420_ biosynthesis genes and F_420_-dependent oxidoreductases. Metatranscriptomic analysis indicates that F_420_-dependent oxidoreductases are highly expressed by Marsarchaeota living in microbial mats. These enzymes were hypothesized to play a role in the metabolism of extracellular sulfonates, although there is limited phylogenetic or biochemical evidence to support this (Jay *et al*. 2018). The candidate phyla Bathyarchaeota and Verstraetearchaeota are also predicted to produce F_420_ (Table 4; Evans *et al*. 2015; Vanwonterghem *et al*. 2016; Zhou *et al*. 2018). Based on the analysis of metagenome derived genomes (MAGs) from these species, Verstraetearchaeota are predicted to be capable of F_420_-dependent hydrogenotrophic methanogenesis (Fig. 6), the first example of an archaeon capable of this process to be discovered outside of the Euryarchaeota (Berghuis *et al*. 2019; Evans *et al*. 2019). Based on the presence of genes homologous to the methyl-CoM reducing complex Mcr, it was originally suggested that Bathyarchaeota are also capable of methylotrophic methanogenesis (Evans *et al*. 2015). However, the phylogenetic grouping of the Mcr genes present in Bathyarchaeota indicates that they utilize this complex for F_420_-dependent anaerobic alkane oxidation (Fig. 6), rather than methanogenesis, similarly to the recently identified candidate genus Syntrophoarchaeum and potentially the candidate phylum Helarchaeota (Laso-Pérez *et al*. 2016; Chen *et al*. 2019; Evans *et al*. 2019; Seitz *et al*. 2019).

##### Asgard archaea

The Asgard archaea are a recently discovered archaeal superphylum that includes the Lokiarchaeota, Thorarchaeota, Odinarchaeota, Heimdallarchaeota, Helarchaeota and Hermodarchaeota (Bulzu *et al*. 2019; Seitz *et al*. 2019; Spang *et al*. 2019). Phylogenetically, the Asgard archaea are the closest archaeal relatives of eukaryotes (López-García and Moreira 2019; Spang *et al*. 2019), and it has been proposed that eukaryotes evolved from a metabolic symbiosis between an Asgard archaeon and an Alphaproteobacterium that gave rise to the mitochondrion (López-García and Moreira 2019; Imachi *et al*. 2020). The reconstruction of metabolic networks of Asgard archaea from metagenome-assembled genomes indicates that they exhibit high metabolic diversity both within and between different phyla with respect to energy source, electron donor, carbon source and electron acceptor preferences (Bulzu *et al*. 2019; Seitz *et al*. 2019; Spang *et al*. 2019; Orsi *et al*. 2020). Members of Loki-, Heimdall- and Hel-archaeota possess all genes required for F_420_ biosynthesis, while available MAGs for Odin- and Thor-archaeota contain several of these genes (Table 4). Like members of the Euryarcheota and TACK lineages of Archaea, members of the Asgard archaea likely utilize F_420_-dependent pathways for carbon fixation and short-chain alkane oxidation, as well as potentially additional unknown processes (Sousa *et al*. 2016; MacLeod *et al*. 2019; Seitz *et al*. 2019; Spang *et al*. 2019; Orsi *et al*. 2020).

### F_420_ production and roles within bacteria

In bacteria, F_420_ has been primarily studied in Actinobacteria. It has been biochemically identified in members of the genera *Mycobacterium*, *Streptomyces*, *Rhodococcus, Nocardia* and *Nocardioides* (Daniels, Bakhiet and Harmon 1985; Eker, Hessels and Meerwaldt 1989; Purwantini, Gillis and Daniels 1997; Ebert, Rieger and Knackmuss 1999; Selengut and Haft 2010), the majority of which are soil saprophytes. F_420_ is not essential for central metabolism in Actinobacteria, though the cofactor is used for a wide range of purposes that provide a growth or survival advantage (Ebert, Rieger and Knackmuss 1999; Hasan *et al*. 2010; Taylor *et al*. 2010; Wang *et al*. 2012; Gurumurthy *et al*. 2013; Greening *et al*. 2017; Lee *et al*. 2020). In addition to Actinobacteria, recent biochemical evidence indicates that F_420_ is produced by members of the phylum Chloroflexi and the classes Alphaproteobacteria and Betaproteobacteria (Ney *et al*. 2017a; Braga *et al*. 2019). Spectroscopic analysis suggests members of the candidate phylum Tectomicrobia also produce the cofactor (Lackner *et al*. 2017). The genes required for F_420_ biosynthesis are also encoded in multiple genomes from the cultivated phyla Acidobacteria, Firmicutes and Nitrospinae and the candidate phyla Rokubacteria, Tectomicrobia and Dadabacteria (Wilson *et al*. 2014; Hug *et al*. 2016; Becraft *et al*. 2017; Lackner *et al*. 2017; Ney *et al*. 2017a). Presently, no experimental studies have been performed investigating its biochemical and physiological role in bacterial species outside of Actinobacteria.

#### Roles in Actinobacteria

##### Mycobacteria

The genetic complement for F_420_ biosynthesis is present in all cultured environmental and pathogenic mycobacteria. F_420_ production has been experimentally confirmed in many *Mycobacterium* species including *M. tuberculosis*, *M. smegmatis*, *M. phlei*, *M. bovis* and *M. avium* (Bair, Isabelle and Daniels 2001). Two fast-growing saprophytic members of the genus, *M. smegmatis* and *M. phlei*, produce F_420_ in large quantities (0.3–0.6 µmol per g dry weight; Isabelle, Simpson and Daniels 2002), indicating it plays a significant role in mycobacterial physiology. In addition, F_420_ is produced by the obligate pathogens *M. tuberculosis* and *M. leprae* (Purwantini, Gillis and Daniels 1997; Bair, Isabelle and Daniels 2001), which suggests a conserved physiological function for the cofactor among mycobacteria, as well as a role in survival in the host. A further indication of its significance is that all mycobacterial species contain numerous enzymes known or predicted to utilize F_420_ as a cofactor (Selengut and Haft 2010; Ahmed *et al*. 2015). *M. smegmatis* is predicted to encode 75 F_420_ dependent enzymes (30 of FDOR family, 45 of LLHT family), while *M. tuberculosis* is predicted to encode 33 F_420_-dependent enzymes (15 of FDOR family, 17 of LLHT family; Selengut and Haft 2010; Ahmed *et al*. 2015). In addition to these known classes of F_420_-dependent enzymes, further F_420_-dependent enzymes may be present in mycobacteria, which belong to novel enzyme families and thus cannot be readily identified based on amino acid sequence homology (Kumar 2018). While the function of the majority of F_420_-dependent enzymes in mycobacteria remains poorly understood, recent phenotypic and biochemical studies have shed light on some of their physiological roles (Hasan *et al*. 2010; Bashiri *et al*. 2012; Gurumurthy *et al*. 2013; Ahmed *et al*. 2015; Jirapanjawat *et al*. 2016; Purwantini, Daniels and Mukhopadhyay 2016; Lee *et al*. 2020).

F_420_ is not essential for mycobacterial growth, with mutants deficient in its synthesis or reduction successfully generated in *M. smegmatis* (Purwantini and Mukhopadhyay 2009; Taylor *et al*. 2010; Grinter *et al*. 2020), *M. tuberculosis* (Darwin *et al*. 2003; Manjunatha *et al*. 2006; Gurumurthy *et al*. 2013) and *M. bovis* (Choi, Kendrick and Daniels 2002). However, several studies indicate that F_420_ contributes to the ability of mycobacteria to persist in response to oxygen deprivation, oxidative stress, nitrosative stress, or treatment with antimicrobial compounds (Purwantini and Mukhopadhyay 2009; Gurumurthy *et al*. 2013; Jirapanjawat *et al*. 2016; Lee *et al*. 2020; Rifat *et al*. 2020). F_420_ reduction in the cytoplasm of *Mycobacterium* appears to be solely mediated by the F_420_-reducing glucose 6-phosphate dehydrogenase (Fgd), rather than by Fno, which is employed by most other Actinobacteria (Purwantini, Gillis and Daniels 1997; Bashiri *et al*. 2008; Jirapanjawat *et al*. 2016). In mycobacteria, Fgd is one of two entry points into the reductive pentose phosphate pathway, in addition to the canonical NADP^+^-reducing enzyme. The metabolic coupling of F_420_ reduction of G6P oxidation represents a significant portion of the flux through the pentose phosphate pathway in mycobacteria, with Fgd activity in cell lysates roughly equivalent to NADP-dependent G6P dehydrogenase (Purwantini, Gillis and Daniels 1997, 1998). G6P levels are 100-fold higher in *M. smegmatis* than *E. coli* grown under comparable conditions and may serve as a store of reductant that is mobilized through F_420_ to combat oxidative stress (Hasan *et al*. 2010). Consistent with this hypothesis, mycobacteria use G6P when challenged with redox cycling agents (e.g. menadione), rapidly reduce such compounds using F_420_H_2_-dependent reductases and are hypersusceptible to challenge in strains unable to make or reduce F_420_ (Hasan *et al*. 2010; Gurumurthy *et al*. 2013; Jirapanjawat *et al*. 2016). Mycobacteria unable to produce or reduce F_420_ are also hypersusceptible to nitrosative stress, including from NaNO_2_ and NO (Darwin *et al*. 2003; Purwantini and Mukhopadhyay 2009). In a chemical assay, isolated F_420_H_2_ readily reduces NO_2_, leading to the suggestion that the cofactor may directly quench reactive nitrogen species (Purwantini and Mukhopadhyay 2009). However, the biochemical mechanism of F_420_ dependent oxidative and nitrosative stress resistance in *Mycobacterium* remains to be fully elucidated.

**Figure 7.**
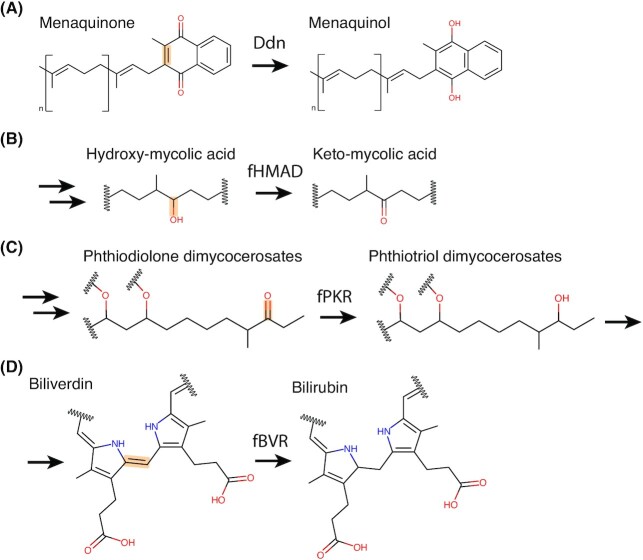
Physiological reactions proposed to be mediated by F_420_-dependent enzymes in mycobacteria. The bond oxidized or reduced is highlighted in orange for each substrate, with the enzyme responsible for the reaction indicated. For the reactions shown in **A**, **B** and **D**, F_420_H_2_ is generated by Fgd through oxidation of G6P. For the reaction shown in **C**, F_420_ oxidizes hydroxymycolic acid to ketomycolic acid at the extracellular face of the cytoplasmic membrane, yielding F_420_H_2_.

Emerging evidence suggests that F_420_H_2_ may also serve as a respiratory electron donor for mycobacteria. The FDOR-A family enzyme deazaflavin nitroreductase (Ddn) from *M. tuberculosis*, as well as its homologs from *M. smegmatis*, can reduce menaquinone at physiologically relevant rates (Fig. 7A; Lee *et al*. 2020). Furthermore, heterologous expression of Ddn stimulated the O_2_ consumption of isolated *M. smegmatis* membranes in an F_420_H_2_-dependent fashion, indicating it supplies F_420_ derived reductant to the respiratory chain. An *M. tuberculosis* mutant lacking this enzyme is impaired in its ability to recover from hypoxia-induced dormancy (Lee *et al*. 2020). However, more systematic studies are required to unravel the contribution of F_420_H_2_ compared to other electron donors in maintaining energy and redox homeostasis in mycobacterial cells.

F_420_ also plays a role in the biosynthesis of the complex lipids that compose the outer envelope of *Mycobacterium*, thereby contributing to the virulence and intrinsic antibiotic resistance of the genus (Cox *et al*. 1999; Dubnau *et al*. 2000; Jain *et al*. 2007; Purwantini and Mukhopadhyay 2013; Purwantini, Daniels and Mukhopadhyay 2016). The outer envelope of pathogenic mycobacteria contains ketomycolic acids, which are important virulence factors (Yuan *et al*. 1998; Dubnau *et al*. 2000; Sambandan *et al*. 2013). Ketomycolic acids are produced by the oxidation of hydroxymycolic acids, after their transport to the extracellular side of the cellular membrane by fHMAD, an F_420_-reducing dehydrogenase of the LLHT family (Fig. 7B; Purwantini and Mukhopadhyay 2013). fHMAD is secreted from the cell via the TAT secretion system in complex with F_420_. As a dehydrogenase, fHMAD does not require a pool of reduced F_420_ to mediate ketomycolic acid formation, allowing it to function extracytoplasmically (Bashiri *et al*. 2012). Phthiocerol dimycocerosates (PDIM) are another family of lipids prevalent in the cell envelope of pathogenic mycobacteria. While likely absent from saprophytic species like *M. smegmatis* (Bansal-Mutalik and Nikaido 2014), in *M. tuberculosis* PDIM constitutes 46% of the total lipids (Wang *et al*. 2020), contributing to cell envelope impermeability and phagosomal escape from host cells (Quigley *et al*. 2017). In *M. bovis*, conversion of phthiodiolone dimycocerosates to PDIM is dependent on reduced F_420_H_2_ provided either enzymatically by Fgd or added exogenously to cell lysates. Based on sequence analysis, it is predicted that F_420_H_2_-dependent LLHT (phthiodiolone ketoreductase, fPKR) is responsible for the reduction of phthiodiolone dimycocerosates to phthiotriol dimycocerosates, the penultimate step in PDIM synthesis (Fig. 7C; Siméone *et al*. 2007; Purwantini, Daniels and Mukhopadhyay 2016). Suggestive of further roles for F_420_-dependent enzymes in lipid biosynthesis, proteomic analysis of the FDOR-AA family in mycobacteria indicates these enzymes are membrane-associated and their genetic context suggests they play a role in lipid synthesis (Ahmed *et al*. 2015). Synthesis of the complex lipid-rich mycobacterial outer envelope requires a high level of biosynthetic complexity (Kolattukudy *et al*. 1997; Bansal-Mutalik and Nikaido 2014; Marrakchi, Lanéelle and Daffé 2014), which may be provided by F_420_-dependent enzymes, thereby helping to explain their abundance and diversity in mycobacterial species.

F_420_H_2_-dependent reductases also provide a reductive detoxification system in mycobacteria, providing the ability to inactivate a range of exogenous compounds with antimicrobial activity (Jirapanjawat *et al*. 2016). *M. smegmatis ΔfbiC* and *Δfgd* strains are hypersensitive to a range of antimicrobial compounds, including quinone analogs (e.g. menadione), coumarin derivatives (e.g. methoxsalen), arylmethane dyes (e.g. malachite green) and quinolones (e.g. oxolinic acid; Guerra-Lopez, Daniels and Rawat 2007; Hasan *et al*. 2010; Jirapanjawat *et al*. 2016). The intrinsic resistance of wild-type *M. smegmatis* to these compounds is attributed to the large number of FDORs it uses. Numerous purified FDORs from *M. smegmatis* have been shown to promiscuously reduce members of the above chemical classes to varying degrees (Jirapanjawat *et al*. 2016; Greening *et al*. 2017). In support of the role of FDORs in reductive detoxification, wildtype *M. smegmatis* can reduce methoxsalen, malachite green and methyl violet added to cultures, but *ΔfbiC* and *Δfgd* strains cannot (Guerra-Lopez, Daniels and Rawat 2007; Jirapanjawat *et al*. 2016). Importantly, *M. smegmatis ΔfbiC* and *Δfgd* strains only display a modest increase in sensitivity to the clinically utilized antimycobacterials, including rifampicin, isoniazid and clofazimine, suggesting it lacks F_420_-dependent enzymes capable of reducing them (Jirapanjawat *et al*. 2016). While some FDORs from *M. tuberculosis* also promiscuously reduce exogenous compounds (Taylor *et al*. 2010; Cellitti *et al*. 2012), it remains to be determined whether F_420_H_2_-dependent reductases provide an analogous detoxification system in obligately pathogenic mycobacteria.

Despite our growing understanding of the general role of F_420_ dependent processes in *Mycobacterium*, few F_420_-dependent enzymes have a defined physiological function (Selengut and Haft 2010; Ahmed *et al*. 2015). In addition to those discussed above, an FDOR-B enzyme purified from *M. tuberculosis* is proposed to be an F_420_H_2_-dependent biliverdin reductase; the enzyme reductively converts biliverdin to bilirubin, a potent antioxidant that may play a role in resisting host-induced oxidative stress, though it remains to be established if this activity occurs physiologically (Fig. 7D; Biswal *et al*. 2006; Ahmed *et al*. 2015). To fully understand the role of F_420_ in *Mycobacterium*, further work is required to systematically characterize the phenotypes associated with this cofactor, including its role in resistance to antimicrobials, redox stress and hypoxia. Additionally, while F_420_ plays a role in mycobacterial physiology, the extent to which the cofactor is required for the long-term persistence of *M. tuberculosis* in the host is unclear. To reconcile the physiology with biochemical mechanisms, the role of specific F_420_-dependent enzymes in mediating the reactions behind these phenotypes needs to be determined through genetic and biochemical analysis.

##### Streptomycetes

**Figure 8.**
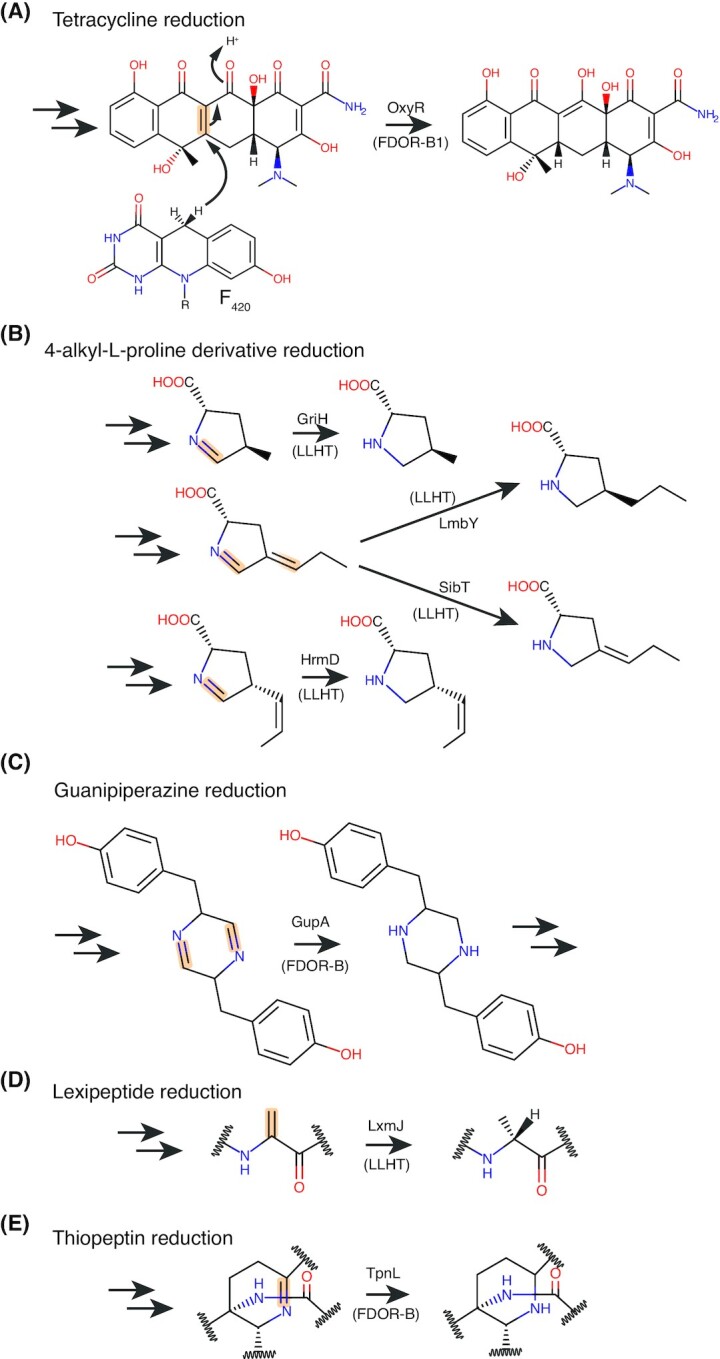
Reactions proposed to be mediated by F_420_-dependent reductases in streptomycetes. The bond reduced is highlighted in orange for each substrate, with the enzyme responsible for the reaction indicated. F_420_H_2_ for the reactions shown is generated by the enzyme Fno via the oxidation of NADPH.

In *Streptomyces* species, F_420_ plays an important role as a cofactor for enzymes involved in the synthesis of structurally diverse antibiotics and secondary metabolites (Wang *et al*. 2013; Ichikawa, Bashiri and Kelly 2018; Steiningerova *et al*. 2020; Tao *et al*. 2020). While it was not formally identified at the time, one of the earliest instances of F_420_ isolation was from *Streptomyces aureofaciens*, where it was shown to mediate the final hydrogenation step in chlorotetracycline biosynthesis (McCormick *et al*. 1958; Miller *et al*. 1960). More recently, it was shown that an F_420_H_2_-dependent FDOR-B family enzyme catalyzes the final reduction of the C5a-C11a double bond of the dehydrooxytetracycline precursor of several tetracycline variants (Fig. 8A; Wang *et al*. 2013). These enzymes are designated OxyR, CtcR and DacO4 in the oxytetracycline/tetracycline, chlorotetracycline and dactylocycline biosynthesis pathways respectively (Wang *et al*. 2012, 2013).

A group of F_420_H_2_-dependent reductases from the LLHT superfamily contribute to the biosynthesis of 4-alkyl-L-proline derivatives (APDs) in various streptomycetes (Steiningerova *et al*. 2020). APDs are biosynthetic precursors for lincosamide and griselimycin antibiotics (Peschke *et al*. 1995; Lukat *et al*. 2017), several pyrrolobenzodiazepines (PBDs) with antitumorigenic and antibiotic properties (e.g. tomaymycin, sibiromycin, anthranmycin; Li *et al*. 2009a, b; Steiningerova *et al*. 2020), and the quorum-sensing peptide hormaomycin (Höfer *et al*. 2011). These F_420_H_2_-dependent LLHT reductases, named Apd6s, are present in the biosynthetic gene clusters (BGCs) for these secondary metabolites and perform the final reduction step in APD biosynthesis (Fig. 8B; Steiningerova *et al*. 2020). These Adp6 enzymes strikingly differ in the reduction reactions they perform. Apd6s from PBD and hormaomycin biosynthesis only reduce the endocyclic imine double bond of the ADP precursor, whereas the Adp6 enzyme associated with lincomycin biosynthesis also reduces the more inert exocyclic double bond of its 4-substituted Δ1-pyrroline-2-carboxylic acid substrate (Fig. 8B; Steiningerova *et al*. 2020). These differences in Adp6 specificity lead to variably saturated APD moieties that help mediate the biological function of the final compound that contains them (Steiningerova *et al*. 2020). Bioinformatic analysis indicates that Adp6 homologs are widely distributed within several bacterial phyla and often associated with BGCs of unknown function, suggesting they mediate the formation of novel APD-containing molecules (Steiningerova *et al*. 2020).

An F_420_H_2_-dependent reductase of the FDOR-B superfamily from *Streptomyces chrestomyceticus*, designated GupA, forms part of the BGC for guanipiperazines A and B. These compounds are formed through the condensation of two L-tyrosine molecules forming a dihydropyrazine ring that is reduced by GupA to form the piperazine ring found in the final product (Fig. 8C). While the function of these compounds is unknown, homologues of GupA and other components of the guanipiperazine BGC are widespread in *Streptomyces* species (Shi *et al*. 2021).

Other F_420_-dependent enzymes form part of the biosynthetic pathways of diverse posttranslationally modified peptide antibiotics. The BGC of the recently discovered lexapeptide, a class V lanthipeptide produced by *Streptomyces rochei*, contains the F_420_H_2_-dependent LLHT LxmJ that catalyzes the reduction of a lexapeptide dehydroalanine moiety to D-Ala (Fig. 8D). This reduction increases the potency of lexapeptide towards a panel of Gram-positive bacteria (Tao *et al*. 2020). Additionally, an F_420_H_2_-dependent FDOR-B family reductase designated TpnL is present in the BGC of the thiopeptide thiopeptin produced by *Streptomyces tateyamensis*. TpnL mediates the reduction of an imine within the dehydropiperidine moiety of thiopeptin, yielding a modified piperidine-containing product (Fig. 8E; Ichikawa, Bashiri and Kelly 2018). TpnL homologs are present in many known or predicted thiopeptide BGCs and form a distinct clade from other FDOR-B sequences (Ahmed *et al*. 2015; Ichikawa, Bashiri and Kelly 2018). Also of note is the BGC of the pyrroloquinoline alkaloid ammosamide from *Streptomyces* sp. CNR-698, which contains the putative F_420_-dependent FDOR-B protein Amm4. The authors predict Amm4 is an oxidase involved in primary amide biosynthesis, based on the accumulation of an ammosamaic acid shunt product in an *Δamm4* producing strain (Jordan and Moore 2016).

The chemically diverse nature of the secondary metabolites with biosynthetic pathways containing F_420_-dependent enzymes, as well as the involvement of the structurally unrelated FDOR and LLHT enzyme families, demonstrates that F_420_-dependent enzymes are versatile biosynthetic tools for streptomycetes. An abundance of BCGs containing predicted F_420_-dependent enzymes indicates that F_420_ is likely to be utilized in the biosynthesis of many more secondary metabolites than those currently identified experimentally. For example, putative F_420_-dependent LLHTs are also encoded in the BGCs for a coronafacoyl phytotoxin produced by the plant pathogen *Streptomyces scabiei* (Bown *et al*. 2016), the aminoglycoside kasugamycin produced by *Streptomyces kasugaensis* (Ikeno *et al*. 2006), and mitomycin C produced by *Streptomyces lawendulae* (Mao, Varoglu and Sherman 1999). However, further experimentation is required to support the F_420_-dependence of these enzymes and their specific role in the biosynthesis of these compounds. At odds with our increasing knowledge of the role of F_420_ in secondary metabolism in streptomycetes, virtually nothing is known about its role in primary metabolism, or whether it shares some physiological roles to those described for its fellow actinobacteria genus *Mycobacterium*.

##### Other Actinobacteria

F_420_ is widely produced by other Actinobacteria including *Rhodococcus*, *Nocardia* and *Nocardioides* (Daniels, Bakhiet and Harmon 1985; Purwantini, Gillis and Daniels 1997; Ebert, Rieger and Knackmuss 1999). Bacteria from these genera utilize F_420_H_2_-dependent reductases from the LLHT superfamily to mobilize the explosive picrate (2,4,6-trinitrophenol) and related compounds (e.g., 2,4-dinitrophenol, 2,4-dinitroanisole) for degradation (Ebert, Fischer and Knackmuss 2001; Fida *et al*. 2014). Due to this capability, several actinobacterial strains such as *Rhodococcus opacus* and *Nocardioides simplex* can grow on picrate as their sole carbon and nitrogen source (Lenke *et al*. 1992; Ebert, Fischer and Knackmuss 2001). Other than its role in the remediation of nitroaromatic xenobiotics (Ebert, Rieger and Knackmuss 1999; Ebert, Fischer and Knackmuss 2001; Fida *et al*. 2014), little is known about the physiological roles of F_420_ in these Actinobacteria. Consistent with its role in picrate degradation, F_420_ likely contributes to the well-documented ability of soil actinomycetes to biodegrade a wide variety of complex organic compounds, including polycyclic aromatic hydrocarbons (McCarthy and Williams 1992; Schrijver and Mot 1999).

#### Roles in other bacteria

The production of F_420_ by bacteria outside of the phylum actinobacteria was only recently experimentally verified (Ney *et al*. 2017a; Braga *et al*. 2019,2020). As such, no F_420_H_2_-dependent reductases or F_420_-reducing dehydrogenases have been investigated experimentally in these bacteria. However, based on the levels of F_420_ production and analysis of the genomes of F_420_-producing bacteria, some inferences can be made regarding the role of F_420_ in these species. Of the species shown experimentally to produce F_420_, *Thermomicrobium roseum* (Chloroflexi) and *Paraburkholderia rhizoxinica* (Betaproteobacteria) are abundant producers, suggesting F_420_ plays a significant role in the physiology of these organisms (Braga *et al*. 2019). In contrast, F_420_ was only detected in *Oligotropha carboxidovorans* and *Paracoccus denitrificans* in trace quantities, indicating a minor or niche-specific role for the cofactor in these Alphaproteobacteria (Ney *et al*. 2017a). F_420_ producers within Betaproteobacteria and Tectomicrobia only encode Fno among known F_420_-reducing dehydrogenases, suggesting that NADPH is the major or only compound utilized for F_420_ reduction in these bacteria (Ney *et al*. 2017a). Predicted alphaproteobacterial F_420_-producers encode Fgd in addition to Fno, suggesting that G6P is also utilized for F_420_ reduction. In Chloroflexi, Fgd is the most prevalent F_420_-reducing dehydrogenase, but Adf and Fno homologs are also present, suggesting diverse substrates enable F_420_ reduction (Ney *et al*. 2017a).

The ecological niche and physiology of recently identified F_420_ producing bacteria suggest the cofactor is employed in diverse roles, which are at least partially analogous to those identified in Actinobacteria. The phylum Tectomicrobia (*Candidatus* Entotheonella spp.) includes uncultured bacteria that produce diverse bioactive secondary metabolites associated with marine sponges (Lackner *et al*. 2017). These bacteria possess large genomes (>9 Mbps) and are predicted to be F_420_ producers, given they are autofluorescent, possess a full set of F_420_ biosynthetic genes (Table 4) and encode multiple putative F_420_H_2_-dependent reductases (Wilson *et al*. 2014; Lackner *et al*. 2017; Ney *et al*. 2017a; Mori *et al*. 2018). F_420_ likely plays a role in secondary metabolite biosynthesis in Tectomicrobia similar to that of streptomycetes discussed above. Chloroflexi are one of the dominant bacterial phyla found in soils and are reputed for their biodegradative capacities (Björnsson *et al*. 2002; Speirs *et al*. 2019). They generally encode numerous F_420_H_2_-dependent reductases (Ney *et al*. 2017a), suggesting members of the phylum may use F_420_ as a cofactor for biodegradative reductases in a similar manner to *M. smegmatis* or *Nocardia* spp. The betaproteobacterial fungal endosymbiont *P. rhizoxinica* produces a chemically distinct F_420_ variant, 3PG-F_420_ (see section 3.1.2). In *P. rhizoxinica*, 3PG-F_420_ production is greatly enhanced when growing inside its fungal symbiont, suggesting the cofactor facilitates symbiosis. Based on the presence of genes encoding a putative ABC transporter adjacent to the 3PG-F_420_ BCG, *P. rhizoxinica* possibly exports the cofactor to be used by its fungal partner (Braga *et al*. 2019). Interestingly, the genome of *P. rhizoxinica* lacks genes with homology to known F_420_-dependent enzymes, suggesting that distinct mechanisms of cofactor utilization and cycling occur in this bacterium (Braga *et al*. 2019).

Experimental work is required to establish the role of F_420_ in these bacteria. Metagenomic analysis indicates that F_420_ producers are prevalent among aerobic soil bacteria (Ney *et al*. 2017a), suggesting it is widely used for its versatile biosynthetic and biodegradative properties. As a result, F_420_-dependent enzymes may directly affect the microbial and chemical composition of soils, by providing bacteria with the ability to both synthesize and degrade antimicrobial compounds, in an ongoing arms race.

## THE MECHANISM AND EVOLUTION OF F_420_ BIOSYNTHESIS

### Diversity within the F_420_ biosynthetic pathway

**Figure 9.**
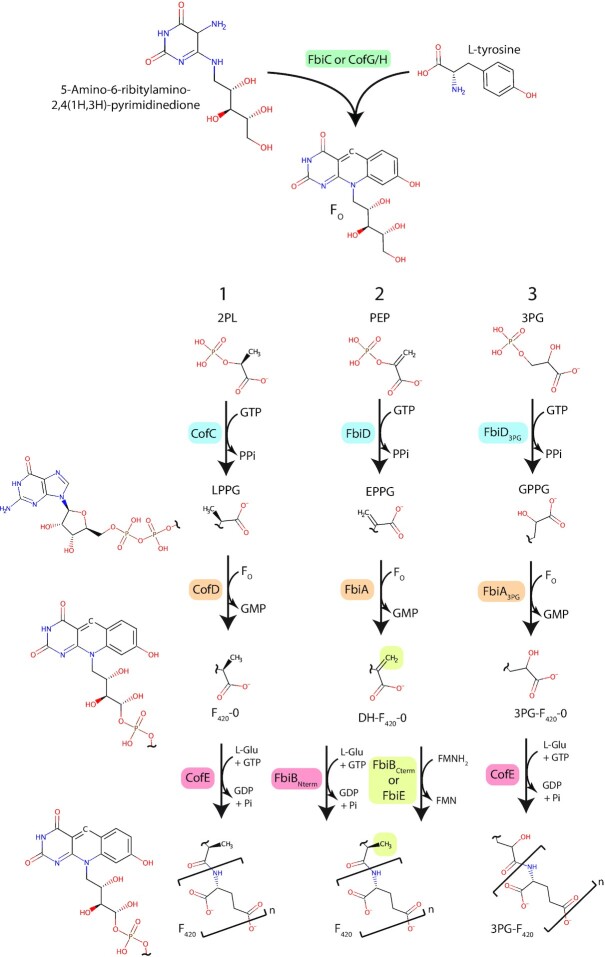
Diverse routes to F_420_ biosynthesis employed by bacteria and archaea. 1 = classical archaeal pathway (Euryarchaeota), 2 = bacterial pathway a (Actinobacteria, Chloroflexi), 3 = bacterial pathway b (Betaproteobacteria). The substrates and mechanisms for F_O_ biosynthesis are shared between all identified pathways. Abbreviated compounds are as follows: PEP, phosphoenolpyruvate; 2PL, 2-phospho-L-lactate; 3PG, 3-phosphoglycerate; EPPG, enolpyruvyl-diphospho-5’-guanosine; LPPG, lactyl-diphospho-5’-guanosine; GPPG, 3-guanosine-5’-disphospho-D-glycerate. The enzymes involved in each biosynthesis step are indicated.

Figure 9 presents the steps in the F_420_ biosynthesis pathway and the enzymes that mediate them. Consistent with the initial discovery of F_420_ in methanogenic Euryarchaeota (Cheeseman, Toms-Wood and Wolfe 1972), initial investigation of the structure and biosynthesis of the cofactor was performed in these organisms (Eirich, Vogels and Wolfe 1978; Jacobson and Walsh 1984; Li *et al*. 2003a). The F_420_ biosynthesis pathway in methanogenic Euryarchaeota was established and was assumed to be universal to all F_420_ producing organisms (Greening *et al*. 2016; Bashiri *et al*. 2019). However, recent investigation of F_420_ biosynthesis in bacteria has revealed that divergent pathways for F_420_ biosynthesis are employed in different organisms. These differences originate from variation in the substrate compound utilized to link the F_O_ head group of F_420_ to its poly-glutamate tail (Bashiri *et al*. 2019; Braga *et al*. 2019; Grinter *et al*. 2020). Based on current experimental evidence, the F_420_ biosynthesis pathway occurs via three variant pathways, found in Euryarchaeota, Actinobacteria and Proteobacteria, respectively (Fig. 9). However, future investigation may reveal further diversity in the F_420_ biosynthesis pathway.

#### A note on nomenclature

Different nomenclature has been applied to F_420_ biosynthetic enzymes from archaea and bacteria. This reflects the incremental nature of the advance in our understanding of F_420_ biosynthesis, as well as the distant relationships between these domains. However, the continued use of different nomenclature is now justified with the recent discovery that F_420_ is synthesized through distinct routes in these domains. Nevertheless, the nomenclature has become increasingly convoluted in light of these discoveries, together with recent evidence demonstrating multiple horizontal gene transfer of F_420_ biosynthetic genes and gene fusion events. For simplicity, in this review, we will generally refer to F_420_ biosynthesis proteins mediating the archaeal pathway with the prefix ‘Cof’ and those mediating the bacterial pathway with the prefix ‘Fbi’.

In methanogens, the enzymes CofG and CofH mediate F_O_ biosynthesis, CofC and CofD mediate F_420_-0 biosynthesis, and CofE is responsible for the formation of mature F_420_ via the addition of a γ-linked polyglutamate tail. In mycobacteria, the prefix ‘Fbi’ is utilized, with a different lettering system, where the following enzymes are analogous: FbiC is derived from a fusion of CofG and CofH, FbiD and FbiA are similar to CofC and CofD respectively, and FbiB is derived from a fusion of CofE and the nitroreductase (NTR) superfamily protein FbiE. It should be noted that some bacteria possess individual enzymes homologous to CofG, CofH, or CofE of archaeal F_420_ producers; we refer to these by the ‘Cof’ designation, as they are distinct from the corresponding ‘Fbi’ fusion enzymes. Likewise, at least some archaea possess some homologs of ‘Fbi’ fusion enzymes. Finally, the FbiA and FbiD variants from some Betaproteobacteria produce the chemically distinct variant 3PG-F_420_ (Braga *et al*. 2019). The subscript ‘_3PG_’ is applied to these enzymes.

#### Comparison of pathways

In the F_420_ biosynthetic pathway of Euryarchaeota, 2-phospho-L-lactate (2PL) links the F_O_ head group of F_420_ to its polyglutamate tail (Fig. 9; Grochowski, Xu and White 2008). It has been proposed that 2PL is synthesized by the unidentified lactate kinase CofB, using lactate produced from L-lactaldehyde (Graupner and White 2001; Graupner, Xu and White 2002; Grochowski, Xu and White 2006). 2PL is conjugated to F_O_ via the action of the enzymes CofC and CofD to create F_420_-0 (i.e. F_420_ with no glutamate tail). CofC activates 2PL through condensation with GTP to form the intermediate compound lactyl-diphospho-5‘-guanosine (LPPG; Grochowski, Xu and White 2008). CofD subsequently transfers 2PL from LPPG to F_O_ to form F_420_-0 (Graupner, Xu and White 2002). The activity of CofC is contingent on the presence of CofD, suggesting that these enzymes form a catalytic complex to regulate the production to the LPPG intermediate, which is unstable (Bashiri *et al*. 2019; Braga *et al*. 2019). F_420_-0 is then converted to mature F_420_ via the addition of a variable-length γ-linked glutamate tail by the enzyme CofE (Li *et al*. 2003a; Nocek *et al*. 2007). In archaea, the length of the glutamate tail varies from 4 to 5 in methanogens with cytochromes or 2–3 in those without (Gorris 1994). In some Euryarchaeota, an additional terminal α-linked glutamate is added by the α-L-glutamate ligase CofF (Li *et al*. 2003b).

**Figure 10.**
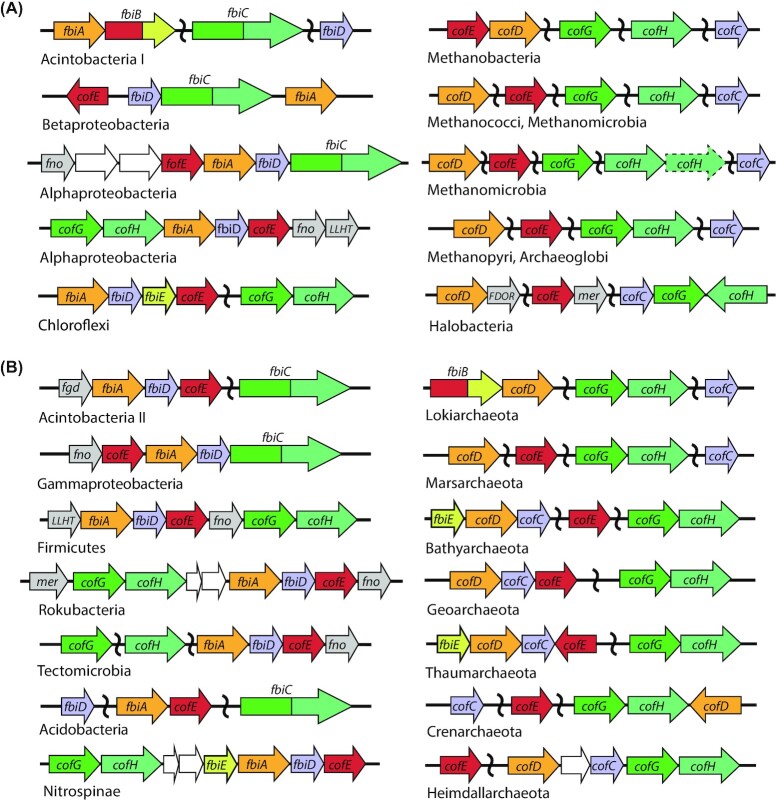
Genetic organization of F_420_ biosynthetic genes in bacteria and archaea. A schematic of the generalized the genetic context of F_420_ biosynthetic genes in experimentally confirmed (panel **A**) and predicted (panel **B**) F_420_-producing bacteria (left) and archaea (right). F_420_ biosynthetic genes are labeled and color-coded. Additional F_420_ related genes are colored grey and labeled as follows: Fgd, F_420_-reducing glucose 6-phosphate dehydrogenase; Fno, F_420_-reducing NADPH dehydrogenase; FDOR, predicted F_420_H_2_-dependent reductase; LLHT, predicted F_420_H_2_-dependent luciferase-like hydride transferase; Mer, F_420_H_2_-dependent CH_2_=H_4_MPT reductase. Hypothetical genes or those with no known F_420_-related function are shown and colored white. Black tilde symbols designate undefined intergenomic space. Gene context is adapted from Ney *et al*. (2017a) or determined directly from available genome sequences.

In mycobacteria, the central metabolic intermediate phosphoenolpyruvate (PEP), rather than 2PL, is utilized as the precursor for F_420_ biosynthesis in this genus (Bashiri *et al*. 2019; Grinter *et al*. 2020). The incorporation of PEP into F_420_ follows an analogous route to that of 2PL in archaea, with the enzymes FbiD and FbiA (homologs of CofC and CofD respectively) first converting PEP into the intermediate enolpyruvyl-diphospho-5’-guanosine (EPPG) and then condensing it with F_O_ to form dehydro-F_420_-0 (DH-F_420_-0), in which the enol group of PEP remains oxidized (Fig. 9; Grinter *et al*. 2020). DH-F_420_-0 is then modified to form mature F_420_ by the dual-functional enzyme FbiB. FbiB possesses an N-terminal domain homologous to CofE, which adds a variable-length γ-linked polyglutamate tail of 2–8 residues (Bashiri *et al*. 2019). The C-terminal domain of FbiB (FbiB_C-term_) reduces the enol group of DH-F_420_ converting it into mature F_420_ (Bashiri *et al*. 2016, 2019). The reduction of DH-F_420_ improves the stability of the molecule by removing the high-energy phosphoenol bond (Braga *et al*. 2020). The Chloroflexi strain *T. roseum* utilizes an independent homolog of FbiB_C-term_ to reduce DH-F_420_, herein referred to as FbiE (Braga *et al*. 2020). Genomic analysis indicates that independent FbiE homologs are present in the genomes of several predicted bacterial and archaeal F_420_ producers, and putative F_420_-producing members of the archaeal phylum Lokiarchaeota possess a dual functional FbiB homolog (Braga *et al*. 2020), suggesting that diverse bacteria and archaea also employ a PEP dependent pathway for F_420_ biosynthesis (Fig. 10B).

A third route for F_420_ biosynthesis is employed by the betaproteobacterium *P. rhizoxinica*, in which 3-phospho-D-glycerate (3PG) is utilized in place of 2PL or PEP for F_420_ biosynthesis. This leads to the formation of the chemically distinct species 3PG-F_420_ and depends on the action of FbiD_3PG_ and FbiA_3PG_, homologs of CofC/FbiD and CofD/FbiA respectively (Fig. 9; Braga *et al*. 2019). The specificity for 3PG over PEP or 2PL appears to originate from FbiD_3PG_, which preferentially mediates the incorporation of 3PG into 3-guanosine-5′-disphospho-D-glycerate (GPPG; Braga *et al*. 2019). A homolog of CofE then adds a variable-length γ-linked polyglutamate tail of 1–6 residues to form mature 3PG-F_420_ (Braga *et al*. 2019).

The selective pressures underlying the rerouting of the F_420_ biosynthesis pathways remain unclear. Considering that CofC/FbiD/FbiD_3PG_ evolved from a common F_420_-producing ancestral enzyme (Ney *et al*. 2017a; Bashiri *et al*. 2019; Braga *et al*. 2019), a substrate switch must have occurred in at least two lineages to yield the three observed biosynthesis pathways. This switch likely occurred to reconcile the precursor utilized for F_420_ production with its presence or level of availability in the metabolite pool of the F_420_-producing organism.

### Structural and biochemical basis

Recently, considerable progress has been made in understanding F_420_ biosynthesis in both bacteria and archaea. Except for CofG/CofH and FbiC, crystal structures have been determined for all enzymes in the F_420_ biosynthetic pathway, with biochemical analysis revealing considerable detail on the catalytic mechanisms employed during F_420_ biosynthesis.

#### Synthesis of F_O_ by CofG/CofH and FbiC

**Figure 11.**
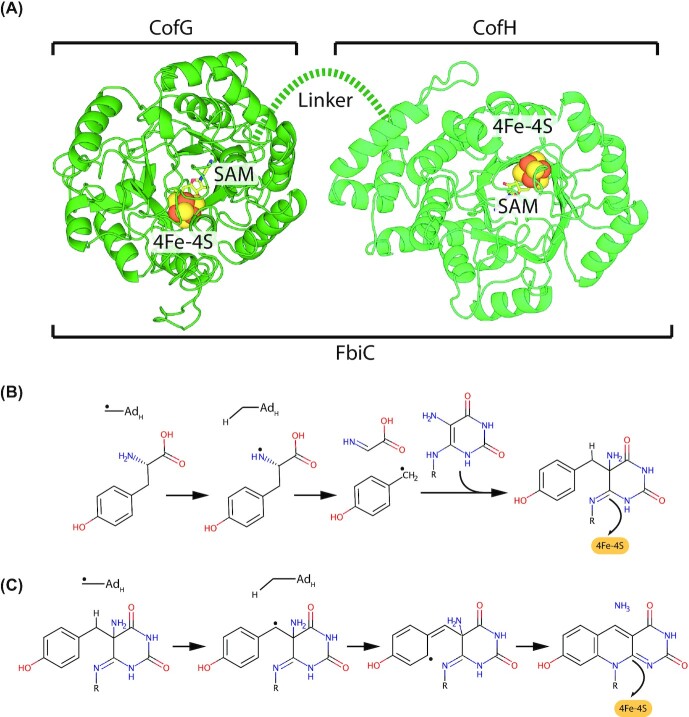
Production of the deazaflavin F_O_ is catalyzed by dual SAM-radical domains. **(A)** Structural model of the SAM radical domains that mediate Fo synthesis, consisting of the two separate proteins CofG and CofH (in archaea and some bacteria) or a single fusion protein FbiC (in bacteria and eukaryotes). Structural models constructed based on homology modeling using Phyre2 based on the structure of MqnE from *Pedobacter heparinus* (PDB ID = 6XI9; Kelley *et al*. 2015). **(B)** A summary of the proposed reaction performed by CofH. **(C)** A summary of the proposed reaction performed by CofG. For the full reaction scheme summarized in panels B and C refer to Philmus *et al*. (2015). R = The F_O_ ribose tail as shown in Fig. 9.

In all studied F_420_ producing organisms, F_O_ is synthesized through a universal mechanism involving two SAM-radical domain enzymes (Decamps *et al*. 2012). In archaea and some bacteria, these domains exist as two separate proteins CofG and CofH, while in Actinobacteria and Proteobacteria they are present in the single fusion protein FbiC (Ney *et al*. 2017a; Fig. 11A). No structures for these enzymes have been determined to date, likely due to the difficulty of working with these oxygen-sensitive proteins (Imlay 2006; Philmus *et al*. 2015). However, mass spectrometric analysis of CofG and CofH reaction products combined with substrate deuteration has provided considerable insight into the catalytic mechanism behind F_O_ synthesis (Decamps *et al*. 2012; Philmus *et al*. 2015). F_O_ is formed by the condensation of L-tyrosine with pyrimidine ribityldiaminouracil (5-amino-6-ribitylamino-2,4 [1H,3H]-pyrimidinedione), which is also a substrate for riboflavin biosynthesis (Bacher *et al*. 2000; Decamps *et al*. 2012). In the first step of this two-step reaction, the 5′-deoxyadenosyl radical generated by CofH abstracts a hydrogen atom from the tyrosine amine, which causes the tyrosine to fragment to form a *p*-hydroxybenzyl radical. This radical then undergoes addition to the double bond of pyrimidine ribityldiaminouracil, with this compound subsequently oxidized by the [4Fe4S] of CofH to yield an intermediate product (Fig. 11B). This product is then accepted by CofG, where the 5′-deoxyadenosyl radical formed by this enzyme extracts a further hydrogen, creating a radical intermediate that undergoes cyclization followed by oxidation by the [4Fe4S] cluster of CofG to yield F_O_ (Fig. 11C; Philmus *et al*. 2015). Despite existing as a fusion protein, the two domains of FbiC appear to function independently, with diffusion rather than direct substrate transfer responsible for the transfer of the product of the FbiC C-terminal domain to the N-terminal domain to complete F_O_ biosynthesis (Philmus *et al*. 2015).

#### Synthesis of LPPG, EPPG and GPPG by CofC, FbiD and FbiD_3PG_

**Figure 12.**
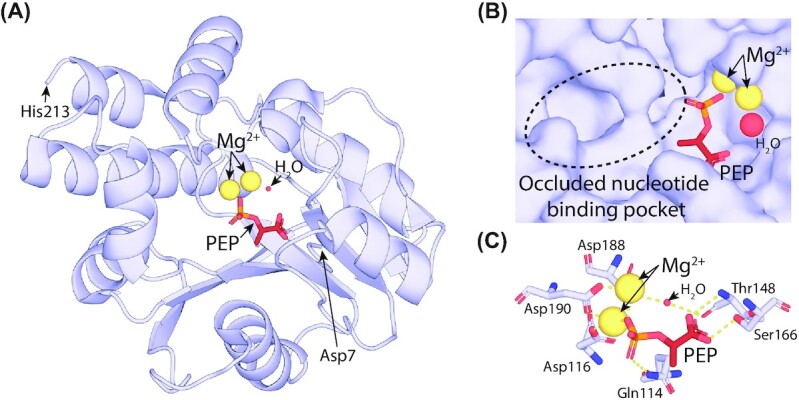
Crystal structure of FbiD from *M. tuberculosis* in complex with PEP substrate. **(A)** Cartoon view of the crystal structure of FbiD in complex with PEP (PDB ID = 6BWH). **(B)** A surface model of the FbiB active site showing the region predicted to bind GTP adopts an occluded conformation. **(C)** Key residues of FbiD involved in the coordination of the PEP substrate and catalytic Mg^2+^ ions. Atomic distances less than 3.2 Å are shown as dashed yellow lines.

The F_O_ is linked to the polyglutamate tail of mature F_420_ via either a 2PL for F_420_ or 3PG for 3PG-F_420_ (Graupner, Xu and White 2002; Braga *et al*. 2019; Grinter *et al*. 2020). In order to activate them for condensation with F_O_, 2PL, PEP or 3PG are condensed with GTP to form LPPG, EPPG and GPPG, respectively (Fig. 9). Despite the differences in their preferred substrate CofC, FbiD and FbiD_3PG_ are homologs thought to share a common catalytic mechanism (Bashiri *et al*. 2019; Braga *et al*. 2019). The crystal structure of Apo-CofC from *Methanosarcina mazei* (PDB ID: 2I5E) was first determined by a structural genomics consortium (2006), demonstrating that the enzyme possesses a nucleotide-binding Rossmann fold, though the lack of substrate in this crystal structure limited the insight of the catalytic mechanism provided by this structure. Recently, the structure of FbiD from *M. tuberculosis* was determined in complex with PEP and two catalytic Mg^2+^ ions (Fig. 12A; Bashiri *et al*. 2019). Based on this structure, key substrate-binding residues were identified (Fig. 12C; Bashiri *et al*. 2019). Further, structural comparison between FbiD and the distantly related bifunctional acetyltransferase/uridyltransferase GlmU reveals a putative GTP-binding pocket (Fig. 12B). This pocket is occluded in the crystal structure of FbiD, which is consistent with the observation that neither purified CofC nor FbiD are active in the absence of their partner enzyme CofD and FbiA (Bashiri *et al*. 2019; Braga *et al*. 2019). This suggests that conformational activation of CofC/FbiD occurs in the presence of CofD/FbiA, likely to prevent the futile production of an unstable product (Bashiri *et al*. 2019).

#### Synthesis of F_420_-0, DH-F_420_-0 and 3PG-F_420_-0 by CofD, FbiA and FbiA_3PG_

**Figure 13.**
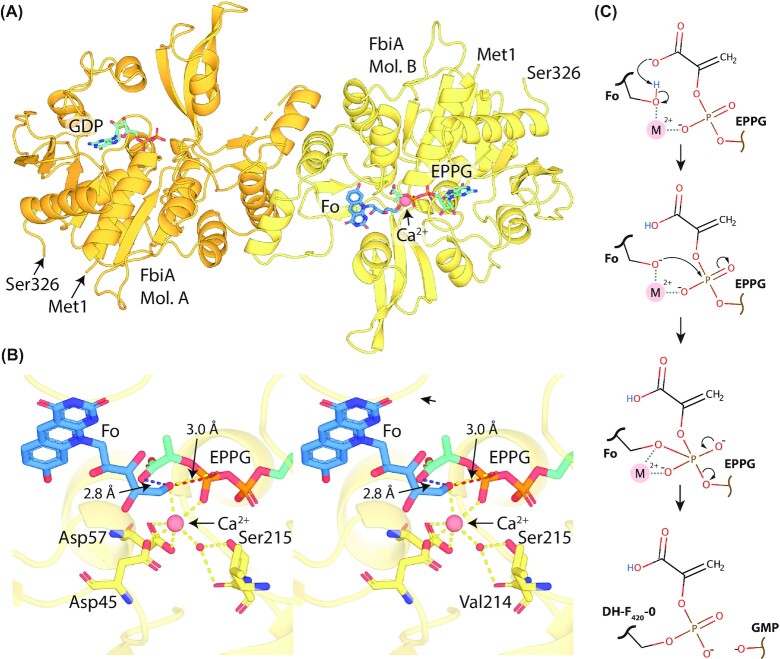
Crystal structure of the FbiA-substrate complex from *M. smegmatis*. **(A)** Crystal structure of the FbiA dimer [PDB ID = 6UW5] in complex with GDP in Mol. A and Fo and EPPG (modeled in place of co-crystallized GDP) in Mol. B. FbiA is shown as a cartoon model, substrate molecules are shown as sticks and a Ca^2+^ ion (likely Mg^2+^ in the active enzyme) is shown as a sphere. **(B)** A cross-eye stereo view of the active site of FbiA Mol. B from panel A, showing key residues for coordinating the FbiA substrate complex as sticks and coordination distances as dotted lines. **(C)** A proposed reaction mechanism for synthesis DH-F_420_-0 by FbiA, in which the carboxyl group of EPPG donates an electron to F_O_, activating it to perform nucleophilic attack on the EPPG β-phosphate.

CofD, FbiA and FbiA_3PG_ are homologous enzymes that mediate the transfer of 2PL, PEP or 3PG from the diphospho-5‘-guanosine intermediate produced by CofC/FbiD to F_O_. This results in the formation of the intermediates F_420_-0, DH-F_420_-0 and 3PG-F_420_-0, with no glutamate moieties (Graupner, Xu and White 2002). The crystal structures CofD from *Methanosarcina mazei* and FbiA from *M. smegmatis* have been determined in the presence of their substrates (Forouhar *et al*. 2008; Grinter *et al*. 2020). These enzymes require a divalent cation for activity, which was absent from the crystal structure of CofD, meaning the catalytically important portions of the F_O_ and GDP substrates were disordered in this structure (Forouhar *et al*. 2008). However, we recently determined the structure of FbiA in complex with F_O_, GDP and Ca^2+^, providing a clear picture of the catalytic complex of this enzyme (Fig. 13A; Grinter *et al*. 2020). The catalytic metal ion represented by Ca^2+^ in this structure is coordinated by aspartates 45 and 57. Aspartate 46 in CofD, which is equivalent to aspartate 57 in FbiA, is important for catalytic activity, suggesting it is also involved in catalytic metal ion coordination in this enzyme (Forouhar *et al*. 2008). In addition to the two aspartate residues, the catalytic Ca^2+^ ion is further coordinated by a H_2_O molecule, the terminal hydroxyl of F_O_ and the β-phosphate of EPPG (Fig. 13B). This coordination positions the EPPG β-phosphate for nucleophilic attack by the F_O_ terminal hydroxyl, leading to the transfer of PEP and the creation of the DH-F_420_-0 product (Grinter *et al*. 2020). To mediate this nucleophilic attack, F_O_ requires activation, with likely candidate bases being the carboxyl or β-phosphate group of EPPG. A proposed reaction mechanism based on proton subtraction by the former is presented in Fig. 13C.

#### Addition of the poly-glutamate tail to F_420_ by CofE and FbiB_N-term_

**Figure 14.**
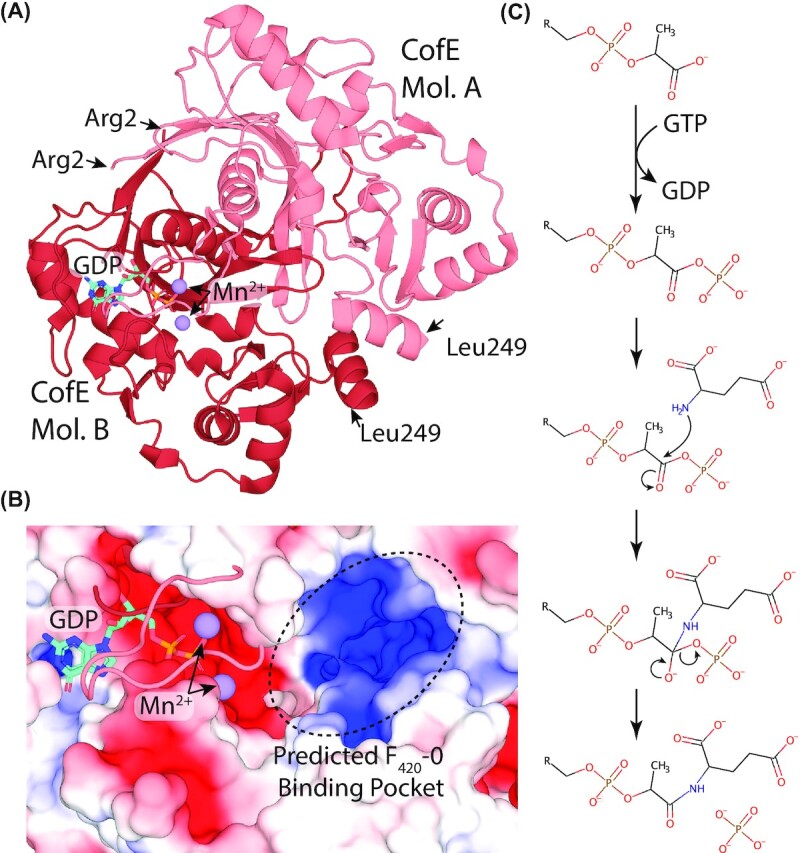
Crystal structure of CofE from *A. fulgidus* in complex with GDP and Mn^2+^. **(A)** Cartoon view of the crystal structure of the functional dimer of CofE [PDB ID = 2PHN]. CofE subunits are shown in pink (Mol. A) and red (Mol. B). Bound GDP and catalytic Mn^2+^ ions are shown for Mol. B only, as stick and sphere representation respectively. **(B)** Electrostatic surface view of the CofE active site showing bound GDP, catalytic Mn^2+^ ions and predicted F_420_-0 binding pocket. **(C)** Proposed catalytic mechanism for the first γ-linked glutamate addition mediated by CofE, R = F_O_ minus terminal hydroxyl.

CofE and the N-terminal domain of FbiB (FbiB_N-term_) are non-ribosomal peptide synthases that perform the final step in F_420_ biosynthesis, adding a variable number of γ-linked glutamate residues to form the F_420_ tail. The crystal structure of CofE from *A. fulgidus* revealed that the protein possesses a novel fold, consisting of an intertwined butterfly-shaped dimer (Fig. 14A; Nocek *et al*. 2007). The GDP and catalytic Mn^2+^ ion bound version of this structure revealed the location of a Y-shaped active site with grooves hypothesized to be responsible for binding F_420_-0 and L-glutamate in addition to GTP (Fig. 14B; Nocek *et al*. 2007). A proposed catalytic mechanism for CofE and FbiB_N-term_, based on that of the nucleotide-dependent tetrahydrofolate:L-glutamate γ-ligase (FPGS; Sheng *et al*. 2000) and UDP-N-acetylmuramoyl-L-alanine:glutamate ligase (MurD; Bertrand *et al*. 1997), involves the activation of the terminal carboxyl of F_420_-0 by the addition of a phosphate group from GTP. Subsequently, the carbonyl carbon of the resulting acyl phosphate undergoes a nucleophilic attack by the glutamate amine, leading to the formation of a tetrahedral intermediate, which breaks down to the final F_420_ product and inorganic phosphate (Fig. 14C; Forouhar *et al*. 2008). Biochemical and genetic evidence indicates that CofE and FbiB_N-term_ are responsible for the addition of both the initial glutamate to F_O_ and extension of the poly-glutamate chain (Bashiri *et al*. 2016,2019). Further research is required to resolve how the active site of these enzymes can perform both the initial and subsequent glutamate additions.

#### Reduction of DH-F_420_ by FbiB_C-term_ and FbiE

**Figure 15.**
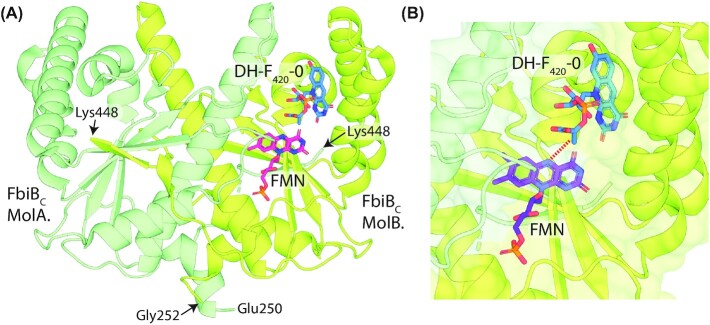
Crystal structure of FbiB_C-term_ from *M. tuberculosis*. **(A)** Cartoon view of the crystal structure of the functional dimer of the FbiB C-terminal domain responsible for the FMNH_2_ mediated reduction of DH-F_420_-0 [PDB IDs = 4XOO, 4XOQ]. FbiB_C-term_ is shown in green and FMN and DH-_F420_-0 (modeled based on the co-crystal structure of F_O_) are shown as sticks. **(B)** A zoomed view of the FbiB_C-term_ active site in complex with FMN and DH-F_420_-0 as in panel A, with a cartoon and transparent atomic surface of the FbiB_C-term_ shown.

The reduction of DH-F_420_ is performed by FbiB_C-term_ in mycobacteria or FbiE in Chloroflexi, homologs that belong to the FMN-dependent NTR superfamily. The crystal structure of the isolated FbiB_C-term_ from mycobacteria has been determined, revealing an intertwined dimer, in complex with either FMN or F_420_ in distinct binding sites (Fig 15A; Bashiri *et al*. 2016). The relative positions of FMN and DH-F_420_-0 were modeled based on these structures, providing a plausible active site and catalytic mechanism for this enzyme (Bashiri *et al*. 2019), where N-5 of FMNH_2_ is ideally positioned for hydride transfer to the DH-F_420_-0 enol group (Fig. 15B).

### Evolution of the F_420_ biosynthesis pathway

**Figure 16.**
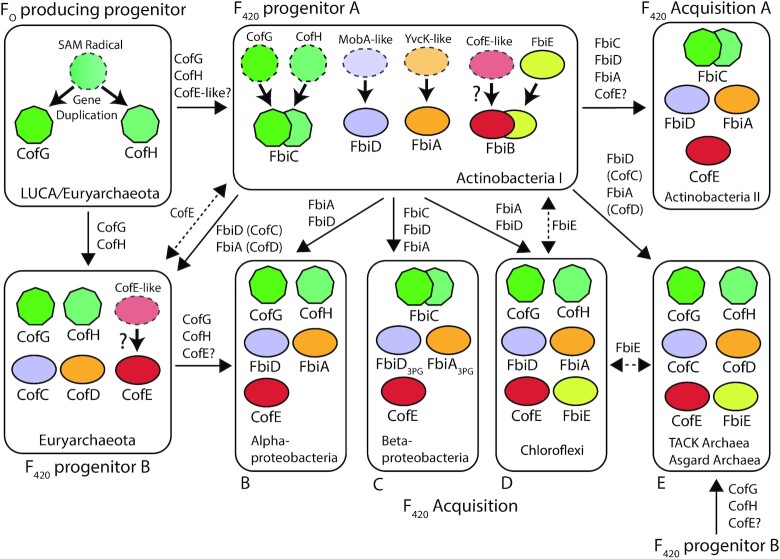
Schematic showing possible events in the evolution of F_420_ and its acquisition by different bacterial and archaeal lineages. Arrows with solid lines indicate potential horizontal transfer of ancestral F_420_ biosynthetic genes. Dashed lines with bidirectional arrows indicate a likely gene transfer event of unknown directionality. Phyla labels are simplified for clarity. Note the figure is a speculative model drawn based on data from sources discussed in the main text and other models are also consistent with these data.

While it is now clear that F_420_ is widely distributed in bacteria and archaea, it is not universally distributed like redox cofactors FMN/FAD and NAD/NADP (Daniels, Bakhiet and Harmon 1985; Ney *et al*. 2017a; Braga *et al*. 2019, 2020). This distribution poses the question of how F_420_ biosynthesis originated and how the genes responsible were disseminated across bacteria and archaea. It has been proposed that the capacity to synthesize F_420_ was present in the last universal common ancestor (LUCA) and was selectively retained by a subset of bacterial and archaeal lineages (Weiss *et al*. 2016). However, current evidence suggests that the F_420_ biosynthesis pathway evolved in a stepwise fashion in archaea and bacteria, with horizontal gene transfer mediating assembly of the complete biosynthesis pathway (Ney *et al*. 2017a). Such inferences are supported by the variable distribution (Fig. 5), genetic organization (Fig. 10) and phylogenetic analysis of the F_420_ biosynthesis genes (Ney *et al*. 2017a). In Fig. 16, we present a schematic of the gene transfer events that potentially occurred during the evolution of F_420_ biosynthesis. By necessity, this model focuses on well-studied F_420_ producers, and the direction of several gene transfer events remains unresolved.

The synthesis of F_O_, as the catalytically active headgroup of F_420_, almost certainly evolved first. F_O_ has near-identical redox properties to F_420_ and can function as a cofactor for F_420_-dependent enzymes in vitro (Jacobson and Walsh 1984; Drenth, Trajkovic and Fraaije 2019). However, the uncharged aromatic structure of F_O_ allows it to readily diffuse across lipid membranes (Bashiri *et al*. 2010), limiting its usefulness as a redox cofactor due to its metabolically costly loss from the cell (Shah *et al*. 2019). The problem of diffusive loss is less acute when F_O_ functions as a chromophore, through its role in DNA repairing photolyase (Malhotra *et al*. 1992; Sancar 1994), given in this case it is tightly associated with its enzyme (Kim and Sancar 1993). Phylogenetic analysis of the enzymes responsible for F_O_ biosynthesis (CofG and CofH) suggests they may have arisen in a deep-branching archaeon (Ney *et al*. 2017a), and were then horizontally acquired by bacteria and certain other archaea. In Actinobacteria, a fusion of the *cofG* and *cofH* genes created *fbiC* (Choi, Kendrick and Daniels 2002; Philmus *et al*. 2015), which was subsequently acquired by several other F_420_-producing bacteria (Ney *et al*. 2017a).

The next stage in the evolution of F_420_ biosynthesis was the addition of a phospho-carboxylic acid group to F_O_ to form F_420_-0, a catalytic intermediate of the current biosynthesis pathway (Bashiri *et al*. 2019; Grinter *et al*. 2020). This modification imparts a negative charge, preventing its diffusion across cellular membranes (Bashiri *et al*. 2010), while not affecting the redox properties of the molecule. Phylogenetic evidence suggests that the ancestors of CofC/FbiD/FbiD_3PG_ and CofD/FbiA/FbiA_3PG_ potentially originated in an actinobacterial ancestor before being laterally acquired by other bacteria and archaea (Nelson-Sathi *et al*. 2015; Ney *et al*. 2017a). This suggests that F_420_ was first employed as a redox cofactor in Actinobacteria, before being horizontally acquired by other bacteria and archaea, including Euryarchaeota. The final stage in the evolution of F_420_ biosynthesis was the addition of the variable-length γ-linked polyglutamate tail to F_420_-0 by the enzyme CofE (Li *et al*. 2003a; Bashiri *et al*. 2019). The polyglutamate tail greatly enhances the affinity and specificity of interactions between F_420_ and F_420_-dependent oxidoreductases, possibly explaining why it arose (Ney *et al*. 2017b; Drenth, Trajkovic and Fraaije 2019). The evolutionary origin of CoE is unclear (Nocek *et al*. 2007; Ney *et al*. 2017a), though the polyglutamate tail synthesized by CofE is present in F_420_ from all currently investigated producing species (Gorris 1994; Li *et al*. 2003a; Bashiri *et al*. 2016; Greening *et al*. 2016; Ney *et al*. 2017a; Braga *et al*. 2019), indicating it is universally important for F_420_ function and has thus been co-inherited with other F_420_ biosynthesis genes. In Actinobacteria, *cofE* underwent a fusion with the DH-F_420_-0 reductase gene *fbiE* to produce the bifunctional *fbiB*.

## APPLICATIONS OF F_420_ BIOSYNTHESIS

### Progress and challenges for the use of F_420_ in industrial catalysis

The hydrogenation reactions performed by F_420_H_2_-dependent reductases are of interest for biocatalysis due to their regio- and enantioselectivity, which can generate up to two chiral centers (Greening *et al*. 2017; Mathew *et al*. 2018). Further, the low redox potential of F_420_ allows it to mediate the reduction of otherwise recalcitrant bonds, including alkenes, enamines, enones, enoates and cyclic imines (Taylor *et al*. 2010; Jirapanjawat *et al*. 2016; Greening *et al*. 2017; Ichikawa, Bashiri and Kelly 2018; Mathew *et al*. 2018; Steiningerova *et al*. 2020). F_420_H_2_-dependent reductases provide an alternative to the nicotinamide-dependent Old Yellow Enzymes (OYEs) for performing these reactions (Stuermer *et al*. 2007; Winkler, Faber and Hall 2018), with some evidence suggesting that F_420_H_2_-dependent reductases can generate reaction products with the opposite stereochemistry than OYEs (Mathew *et al*. 2018). However, while recent work has addressed a number of the challenges associated with utilizing F_420_-dependent enzymes for industrial applications, several further challenges must be addressed before their potential for chemical synthesis can be realized.

Two plausible scenarios exist for the utilization of F_420_H_2_-dependent reductases in the production of industrially relevant compounds. Purified F_420_H_2_-dependent reductases can be utilized, in conjunction with an F_420_-reducing regeneration system, to perform the biocatalytic reduction of the desired substrate. Alternatively, a synthetic biology approach could be employed, utilizing microbes engineered to produce F_420_ and express F_420_-dependent biosynthetic pathways to produce compounds via microbial cell culture. In this section, we will discuss challenges and recent developments relating to the heterologous production of F_420_ and the development of suitable F_420_-dependent enzymes for compound synthesis. These advances apply both to systems utilizing purified F_420_-dependent enzymes and the development of synthetic biological systems utilizing F_420_.

#### Development of high yield F_420_ production

One of the major challenges in the development and utilization of F_420_-dependent enzymes for biotechnological applications is the low yield of F_420_ obtained when purified from native sources (Isabelle, Simpson and Daniels 2002; Mathew *et al*. 2018; Bashiri *et al*. 2019). However, recent advances in our understanding of the F_420_ biosynthesis pathway, as well as successful heterologous production, have improved the prospects of obtaining the cofactor in industrially relevant quantities (Bashiri *et al*. 2019; Grinter *et al*. 2020).

F_420_ was initially purified from methanogenic archaea, such as *Methanobacterium thermoautotrophicum* (Eirich, Vogels and Wolfe 1978, 1979; Isabelle, Simpson and Daniels 2002). However, the relative technical difficulty in culturing these obligate anaerobes led to the identification and optimization of the aerobic actinomycete *M. smegmatis* as an alternative source of F_420_ (Isabelle, Simpson and Daniels 2002). Despite producing F_420_ at levels 5-fold lower than methanogens, the ease of culture and high cell densities achieved by this bacterium led to *M. smegmatis* being largely adopted as the preferred organism for F_420_ production, except in cases where short-chain F_420_-2 is required (Isabelle, Simpson and Daniels 2002; Ney *et al*. 2017b). Heterologous plasmid-based expression of FbiA, FbiB and FbiC in *M. smegmatis* by Bashiri *et al*. increased production of F_420_ from this bacterium 10-fold to levels greater than those produced by methanogens (Bashiri *et al*. 2010). This augmented F_420_ production in *M. smegmatis* is the currently preferred method of F_420_ production (Lapalikar *et al*. 2012; Ahmed *et al*. 2015; Mashalidis *et al*. 2015; Ney *et al*. 2017a; Oyugi *et al*. 2018; Drenth, Trajkovic and Fraaije 2019; Steiningerova *et al*. 2020). However, yields from this method are still unlikely to be compatible with economically viable production on an industrial scale. The estimated maximum yield of F_420_ from this process is ∼3 g/kg dry weight (∼0.9 g/kg wet cell weight; Isabelle, Simpson and Daniels 2002; Bashiri *et al*. 2010). Therefore, considerable further optimization of this system or alternative processes for F_420_ production are required.

One option for large-scale production of F_420_ is the use of a heterologous expression system, which is amenable to optimization through metabolic engineering. Until recently, a perceived bottleneck for heterologous production was the use of 2PL as a substrate in the F_420_ biosynthetic pathway, as it is not produced in significant quantities by bacteria (Graupner and White 2001; Graupner, Xu and White 2002; Bashiri *et al*. 2019). However, the discovery that mycobacterial F_420_ biosynthesis utilizes the abundant metabolite PEP paved the way for its heterologous production (Bashiri *et al*. 2019; Grinter *et al*. 2020). Concurrently, Ney and Greening first successfully produced F_420_ in *E. coli* through the heterologous expression of FbiC, FbiA, CofD and FbiB (Ney 2019). However, the yields for F_420_ produced in *E. coli* were lower than that achieved for purification from *M. smegmatis* or methanogens (Isabelle, Simpson and Daniels 2002; Bashiri *et al*. 2019; Shah *et al*. 2019), meaning considerable further engineering is required to make the system compatible with industrial production. Through the course of their discovery of 3PG-F_420_ production by *P. rhizoxinica*, Braga et. al. independently heterologously produced this chemical F_420_ variant in *E. coli* (Braga *et al*. 2019). While 3PG-F_420_ was only produced in low quantities, it is compatible with F_420_ dependent enzymes from organisms producing the classical version of the cofactor (Braga *et al*. 2019). As such, this work provides an alternative set of enzymes and precursor substrates for heterologous F_420_ production, which may assist in increasing production levels.

Synthetic or semisynthetic synthesis of F_420_-like cofactors is a promising alternative strategy for obtaining large quantities of cofactor compatible with industrial applications. F_O_ can be produced synthetically in large quantities and is catalytically compatible with some F_420_-dependent enzymes (Hossain *et al*. 2015). However, it generally exhibits much lower catalytic efficiency, making it a less than ideal cofactor for industrial applications (Drenth, Trajkovic and Fraaije 2019). Recently, Drenth et. al. utilized a biosynthetic approach to enzymatically phosphorylate the terminal hydroxyl of F_O_, yielding the F_420_ analog F_O_-5′-phosphate (FOP). FOP was functional as a cofactor for both F_420_-reducing dehydrogenases and F_420_H_2_-dependent reductases with a higher catalytic efficiency than F_O_. However, a reduction in catalytic efficiency compared to F_420_ was observed depending on the enzyme employed (2- to 22-fold reduction; Drenth, Trajkovic and Fraaije 2019; Martin *et al*. 2020). This reduction in efficiency reinforces the importance of the F_420_ polyglutamate tail in protein-cofactor interactions (Ney *et al*. 2017b) and suggests that FOP use will be limited to compatible enzymes or those which have been engineered to suit this cofactor. Currently, no definitive solution exists to cheaply and efficiently produce F_420_ or a suitable analog that can be utilized by enzymes with a high level of catalytic efficiency. However, the recent progress discussed above can likely be built upon to provide a solution to this bottleneck in the near future.

#### Development of an efficient F_420_ reduction system

To harness their potential for asymmetric hydrogenation on an industrial scale, F_420_H_2_-dependent reductases require a source of reduced F_420_H_2_. Ideally, F_420_H_2_ would be regenerated from enzymatically oxidized F_420_ via a mechanism integral to the reaction system, providing a high F_420_H_2_/F_420_ ratio and a sustained source of the reduced cofactor to maximize reaction yields. While it may be possible to directly reduce F_420_ by electrochemical or photochemical means (Wichmann and Vasic-Racki 2005), this has not been comprehensively investigated. As such, enzymatic regeneration of F_420_ using existing F_420_-reducing dehydrogenases is currently the most practical means of cofactor regeneration.

F_420_-reducing dehydrogenases that utilize a number of diverse substrates have been identified (Table 2). In their physiological context, these dehydrogenases produce a free pool of reduced cytoplasmic F_420_H_2_, which is bound by F_420_H_2_-dependent reductases and utilized to directly transfer hydride to their substrate (Ahmed *et al*. 2015, 2016; Greening *et al*. 2017; Mathew *et al*. 2018). This system differs mechanistically from OYEs, which tightly bind a FMN molecule that is first reduced by NAD(P)H before substrate reduction (Stuermer *et al*. 2007; Toogood, Gardiner and Scrutton 2010). The independent nature of F_420_-reducing dehydrogenases and F_420_H_2_-dependent reductases provides flexibility compared to OYEs, allowing for the use of different substrates for cofactor regeneration by F_420_-reducing dehydrogenases. Several F_420_-reducing dehydrogenases have been produced recombinantly and structurally characterized (Table 2; Warkentin *et al*. 2001; Aufhammer *et al*. 2004,2005; Bashiri *et al*. 2008; Allegretti *et al*. 2014), facilitating enzyme production and optimization via structure-guided protein engineering. However, to provide an economically viable solution to F_420_ reduction for industrial catalysis, the enzyme employed must be stable and readily producible in large quantities. Additionally, it must reduce F_420_ with reasonable catalytic efficiency and its substrate must be cheap and readily obtainable. Considering these criteria, some F_420_-reducing dehydrogenases are more attractive targets than others for industrial cofactor regeneration.

Enzymes originating from methanogenic archaea, which utilize H_2_ (Frh) or formate (Ffd) for F_420_ reduction, are superficially attractive targets due to the low cost of their substrates and the lack of contaminating solutes resulting from their oxidation (Shah *et al*. 2019). However, both of these enzymes are multi-subunit proteins that utilize complex transition metal cofactors for substrate oxidation and transfer the resulting electrons to F_420_ via multiple iron-sulfur clusters (Schauer and Ferry 1986; Vitt *et al*. 2014). The complexity of these enzymes, combined with the oxygen sensitivity of their metal-containing functional groups, means they are unlikely to be a practical solution for F_420_-reduction (Baron and Ferry 1989; Vitt *et al*. 2014). F_420_ reduction utilizing NADPH as a hydride donor, via the enzyme Fno, represents a more attractive means of cofactor regeneration (Berk and Thauer 1997; Kumar *et al*. 2017). Fno is a small single subunit protein, which is produced by a wide range of bacteria and archaea, providing numerous homologs from which to select an industrially compatible enzyme (Eirich and Dugger 1984; Kunow *et al*. 1993; Le *et al*. 2015; Kumar *et al*. 2017). Representative crystal structures of Fno from thermophilic species have been determined, providing the basis for optimization via protein engineering (Kunow *et al*. 1993; Kumar *et al*. 2017). Drawbacks for the use of Fno for cofactor regeneration include the relative expense of NADPH, which is required in stoichiometric quantities to the target for reduction (unless an additional enzyme and substrate is added for NADP^+^ regeneration), as well as the presence of contaminating NADP^+^ in the final reaction mix. The use of G6P for F_420_ regeneration via the enzyme Fgd is another option that has similar advantages and disadvantages to NADPH. Fgd is a single-chain protein that can be recombinantly produced and for which a crystal structure has been determined (Purwantini and Daniels 1996; Bashiri *et al*. 2008). However, G6P is relatively expensive and its use in F_420_ reduction leads to the generation of the by-product 6-phosphoglucono-D-lactone. Recently homologs of Fgd with significant activity towards other sugar phosphates were identified (Mascotti *et al*. 2018). These enzymes, named F_420_-reducing sugar-6-phosphate dehydrogenases (Fsd), also exhibit low levels of F_420_ reductase activity with non-phosphorylated sugars (Mascotti *et al*. 2018). While the rates of F_420_ reduction with these sugars were too low to be catalytically useful, they suggest that through protein engineering Fsd could be adapted to utilize more economical non-phosphorylated sugars as substrates (Mascotti *et al*. 2018).

The F_420_-reducing secondary alcohol dehydrogenase Adf is the most promising target for F_420_H_2_ regeneration on an industrial scale. Adf is a relatively small (37 kDa) single chain enzyme produced by thermostable organisms, which can utilize inexpensive secondary alcohols like isopropanol to reduce F_420_ with a reasonable catalytic efficiency (Bleicher and Winter 1991). The product of this reaction is a volatile ketone (e.g. acetone), which can be readily separated from the reaction product. The standard potential for the reduction of acetone to isopropanol is −290 mV, higher than that of F_420_/F_420_H_2_ (−340 mV; Thauer, Jungermann and Decker 1977; Jacobson and Walsh 1984), meaning that a relatively high concentration of substrate (>100 mM) would be needed to ensure efficient F_420_ reduction. However, Adf has been shown experimentally to tolerate isopropanol up to 100 mM with no significant reduction in activity (Martin *et al*. 2020). Relatively high concentrations of secondary alcohols are often well tolerated by enzymes and this tolerance could be improved for F_420_H_2_-dependent reductases by protein engineering (Doukyu and Ogino 2010). Moreover, the addition of secondary alcohols could increase the solubility of enzyme substrates with poor solubility. In summary, Adf is a simple protein with high stability, that can be produced recombinantly, has good catalytic efficiency, an inexpensive substrate and produces a volatile product. As such, of the currently characterized F_420_ reducing enzymes, it is the only candidate that fulfills all the above criteria for industrial applications.

#### Discovery and engineering of F_420_H_2_-dependent reductases

The range of biological reduction reactions performed by F_420_-dependent enzymes discussed above indicates their potential for performing reduction reactions. The diversity of molecules identified as physiological substrates for F_420_H_2_-dependent reductases, encompassing large and small soluble molecules, as well as hydrophobic molecules and lipids, also reflects their versatility as biocatalysts (Figs 7 and 8; Wang *et al*. 2013; Purwantini, Daniels and Mukhopadhyay 2016; Greening *et al*. 2017; Lee *et al*. 2020; Steiningerova *et al*. 2020; Tao *et al*. 2020). Furthermore, the abundance of predicted F_420_-dependent enzymes encoded in microbial genomes indicates that currently-characterized F_420_-mediated reactions represent a small subset of those that exist in nature (Ahmed *et al*. 2015; Ney *et al*. 2017a; Mascotti *et al*. 2018). This indicates that there is a wealth of F_420_-dependent enzymes with potential for use in industrial catalysis.

The use of F_420_H_2_-dependent reductases to perform industrially important reduction reactions currently represents the most promising application of F_420_ to industrial processes (Greening *et al*. 2017; Mathew *et al*. 2018; Drenth, Trajkovic and Fraaije 2019). F_420_H_2_-dependent reductases can reduce a range of activated alkenes (quinones, coumarins, enones, enals, pyrones and pyrans), unsaturated nitrogen-containing compounds (imines, enamines and nitrobenzenes) and secondary alcohols (Figs 7, 8 and 18; Taylor *et al*. 2010; Jirapanjawat *et al*. 2016; Greening *et al*. 2017; Mathew *et al*. 2018; Martin *et al*. 2020). Additionally, reactions performed by F_420_H_2_-dependent reductases tend to be stereoselective, particularly for larger, more complex substrates (Mathew *et al*. 2018; Martin *et al*. 2020), leading to the creation of chiral products that are often essential for use as pharmaceutical drugs or agricultural pesticides (Patel 2001; Nguyen, He and Pham-Huy 2006; Sekhon 2009). In addition to the use of F_420_ for substrate reduction, F_420_-reducing dehydrogenases may also prove useful for performing oxidation during chemical synthesis. The use of fHMAD by pathogenic mycobacteria to oxidatively generate ketomycolic acids provides proof of concept for this (Fig. 7C; Purwantini and Mukhopadhyay 2013). F_420_-reducing dehydrogenases could potentially be employed for substrate oxidation, coupled with F_420_H_2_-dependent reductases, to yield useful oxidation and reduction products from a single process.

**Figure 17.**
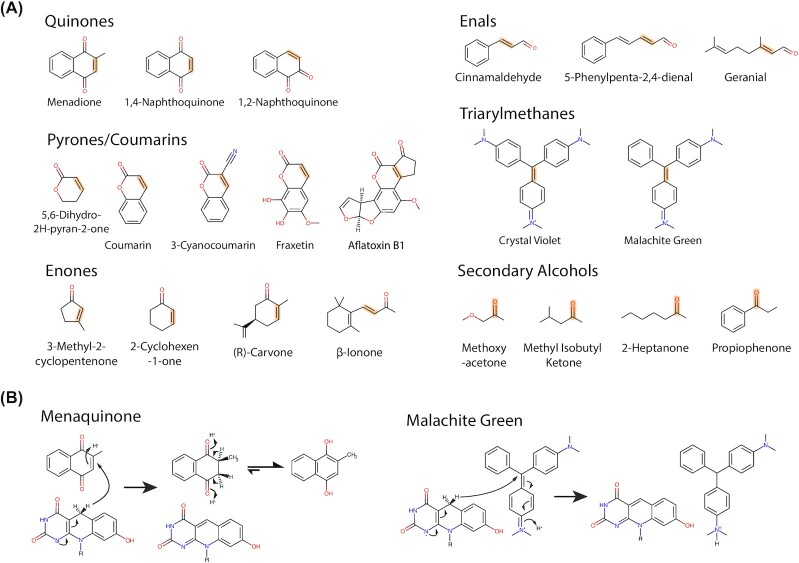
Compounds reduced by purified F_420_-dependent enzymes. **(A)** Classes of compounds shown to be reduced by purified F_420_-dependent enzymes. The reduced bond is highlighted in orange for each compound. The enzymes tested, as well as their substrate range and relative activity levels, are provided in Table 5. **(B)** Proposed reaction schemes for the reduction of menaquinone and malachite green by F_420_H_2_-dependent reductases of the FDOR-A superfamily. In both cases, initial hydride transfer to one carbon atom of an activated alkene is followed by tautomerization yielding the final reaction product.

Some work has been performed characterizing the activity, substrate specificity and product stereochemistry of actinobacterial F_420_H_2_-dependent reductases from the diverse FDOR-A and FDOR-B families (Ahmed *et al*. 2015; Greening *et al*. 2017; Mathew *et al*. 2018). FDORs are small single-chain proteins that are relatively easy to produce recombinantly, making them good targets for industrial catalysis (Ahmed *et al*. 2015, 2016; Greening *et al*. 2017; Mathew *et al*. 2018). Initial work characterizing these enzymes showed that they exhibit significant levels of promiscuous activity towards aflatoxins and some other coumarin derivates, leading to the reductive hydrolysis of these compounds (Taylor *et al*. 2010; Lapalikar *et al*. 2012). Subsequent work showed that purified FDORs are capable of reducing a range of quinones, coumarins, enones, enals, pyrones, pyrans and triarylmethane dyes, with activity and substrate specificity varying widely between enzymes (Fig. 17 and Table 2; Greening *et al*. 2017; Mathew *et al*. 2018). FDOR-A family enzymes investigated displayed relatively high levels of activity towards quinones, in line with their proposed physiological role as menaquinone reductases (Ahmed *et al*. 2015; Lee *et al*. 2020). Mathew *et al*. showed that three diverse actinobacterial FDOR-A enzymes exhibited high levels of enantioselectivity towards a panel of α/β-unsaturated ketones and aldehydes, as well as regioselectivity towards a benzyl-denial compound (Mathew *et al*. 2018). The observed enantioselectivity of these enzymes towards several substrates was the opposite of that observed when the substrates were reduced by OYEs, indicating that FDORs could provide stereochemical flexibility for enzymatic catalysis (Mathew *et al*. 2018). However, the catalytic rates of purified FDORs towards these substrates, where reported, are low, meaning that significant engineering is required to render them suitable for industrial catalysis (Jirapanjawat *et al*. 2016; Greening *et al*. 2017; Mathew *et al*. 2018). Interestingly, a panel of diverse FDORs showed no activity against compounds containing functional groups (nitroimidazoles and imines) known to be reduced by F_420_H_2_-dependent FDORs in a physiological context (Greening *et al*. 2017). This observation, combined with the vastly different activities observed for FDORs towards different substrates with the same functional group, indicates that protein-substrate interactions are important for determining enzyme activity and significant engineering will be required to adapt the substrate binding sites of these enzymes to industrially relevant substrates.

Further investigation into the mechanisms of substrate binding and reduction by FDORs will assist in engineering enzymes with higher catalytic efficiency and predicting product stereochemistry. It has been proposed that proton donation to the F_420_-substrate after initial hydride transfer from F_420_ occurs from N1 of F_420_H_2_ (Fig. 1B; Shah *et al*. 2019). If true, both hydrogenation events occur on the same face of the activated alkene substrate, leading to *cis*-hydrogenation when a disubstituted alkene is reduced by the enzyme. This is in contrast to OYEs where *trans*-hydrogenation products are formed (Hollmann, Opperman and Paul 2020). However, *cis*-hydrogenation by FDORs has not been demonstrated experimentally, and spectroscopic and computational analysis suggests that F_420_H^−^ (in which N1 is deprotonated) is the form of the cofactor utilized by the FDOR-A Ddn (Mohamed *et al*. 2016a). If F_420_H^−^ is the general physiological form of the cofactor used by FDORs, then substrate protonation must instead proceed from solvent or an enzyme sidechain (Mohamed *et al*. 2016a,b; Greening *et al*. 2017). A better understanding of the structural and biochemical basis for the binding of physiological and industrially relevant substrates to FDORs is also required. No crystal structures of FDOR-substrate complexes are available, though some docking analysis has been performed, indicating residues important for substrate binding (Ahmed *et al*. 2015; Greening *et al*. 2017; Lee *et al*. 2020). Systematic structural analysis of diverse FDORs in complex with catalytically relevant substrates will facilitate the identification of key residues involved in substrate binding and catalysis.

LLHTs, another major family of F_420_-dependent enzymes, are also a promising source of enzymes for industrial applications. Fgd and Adf, F_420_-reducing dehydrogenases from this family, have been utilized for F_420_H_2_ generation at a laboratory scale (Jirapanjawat *et al*. 2016; Mascotti *et al*. 2018; Drenth, Trajkovic and Fraaije 2019). Recently, Martin *et al*. utilized Adf for the stereoselective reduction of diverse secondary ketones, with F_420_H_2_ generated by Fgd using G6P as a substrate, or using Adf to drive F_420_ both reduction and oxidation by including high concentrations of isopropanol in the reaction. While this study provides an important proof of concept for the generation of chiral alcohols using F_420_-dependent enzymes, Adf exhibited low apparent activity towards these substrates (Martin *et al*. 2020). Other F_420_-dependent LLHT reductases have yet to be investigated for their catalytic potential. However, LLHTs are utilized by bacteria in the biosynthesis of diverse molecules, including mycolic acids, 4-alkyl-L-proline intermediates and lexapeptides, and in the reductive degradation of picrate (Purwantini and Mukhopadhyay 2013; Purwantini, Daniels and Mukhopadhyay 2016; Steiningerova *et al*. 2020; Tao *et al*. 2020). The ability of LLHTs to perform these diverse reactions, both on large polar and hydrophobic molecules, suggests their biosynthetic potential. Notably, the TIM-barrel fold of LLHTs is unrelated to that of FDORs, possessing a structurally divergent substrate-binding pocket (Fig. 4; Mascotti *et al*. 2018). As such, LLHTs are likely to provide complementary substrate specificity and stereoselectivity to FDORs. Like FDORs, sequenced microbial genomes contain a wealth of putative F_420_-dependent LLHTs with unknown function, many of which are contained within hypothetical secondary metabolite BCGs (Selengut and Haft 2010; Mascotti *et al*. 2018; Steiningerova *et al*. 2020)

In summary, while our current understanding of F_420_-dependent enzymes sets the stage for the use of F_420_ in industrial catalysis, considerable further work is required to develop enzymes suitable for such processes. To realize this goal these enzymes will require a high level of activity towards economically relevant substrates, with favorable kinetic parameters. Also, they will likely need to display high levels of stereoselectivity, yielding a commercially relevant enantiomer. Further, for use in synthetic chemistry, the enzymes will need to be robust, able to withstand relatively harsh extremes of temperature, pH, ionic strength and concentrations of non-polar solvents. Currently, no enzymes with these properties have been described or developed. A possibly promising strategy for the development of such enzymes would be to identify commercially relevant substrates, with chemistry amenable to reduction or oxidation by F_420_-dependent enzymes, which are recalcitrant to currently available synthetic chemical processes. The wealth of F_420_-dependent enzymes present in microbial genomes could be screened for enzymes capable of reducing these substrates to some degree. These enzymes could be then subjected to rigorous structural and biochemical characterization, combined with concerted protein engineering efforts, to produce enzymes suitable for the reduction of these substrates on an industrial scale.

### Ramifications and applications of microbial utilization of F_420_

In addition to the importance of F_420_ in microbial physiology and its potential applications for chemical synthesis, our understanding of the diverse role of the cofactor has wider significance for improving human health and sustainability. As discussed below, hydrogenotrophic methanogens that rely on F_420_ are an important source of global greenhouse gases, while in *M. tuberculosis* F_420_ is important for the activation of nitroimidazole prodrugs and plays an insufficiently characterized role in survival in the host. Our recently acquired knowledge on the biosynthesis and roles of F_420_ can be utilized to develop methanogenesis inhibitors and antitubercular drugs, as well as to predict and alleviate nitroimidazole resistance.

#### Methane mitigation through inhibition of F_420_ dependent enzymes

F_420_-dependent hydrogenotrophic methanogens, notably *Methanobrevibacter gottschalkii* and *Methanobrevibacter ruminantium*, are core members of the foregut microbiota of ruminants (Leahy *et al*. 2010; Henderson *et al*. 2015). Methane emitted by ruminants is a significant driver of global warming, with ruminant methane emissions accounting for approximately 40% of global methane emissions and 5% of global greenhouse gas emissions (Greening *et al*. 2019). Bacteria and archaea that utilize alternative acetogenic or respiratory pathways for H_2_ oxidation, independently of F_420_, are abundant in the rumen (Greening *et al*. 2019). Thus, strategies to inhibit or outcompete hydrogenotrophic methanogens should foster a microbial community that produces less methane, reducing the greenhouse gas emissions from livestock farming (Morgavi *et al*. 2010; Greening *et al*. 2019). Potent methyl-CoM reductase inhibitors, such as 3-nitrooxypropanol, reduce methane production without compromising animal health and productivity (Hristov *et al*. 2015; Duin *et al*. 2016). Given hydrogenotrophic methanogenesis requires F_420_, strategies that inhibit F_420_ production or its use by F_420_-dependent enzymes will also inhibit the growth of these archaea. While specific strategies for the inhibition of F_420_ biosynthesis or dependent enzymes in the rumen have not been reported, several studies indicate this may be possible. The pterin lumazine inhibits methanogen growth and methane formation in pure culture, possibly through its structural similarity to F_420_ (Nagar-Anthal *et al*. 1996), although this effect was less significant in mixed culture (Ungerfeld *et al*. 2004). Recently, in silico screening identified inhibitors of Fno from the methanogen *Methanobrevibacter smithii*, some of which bind the enzyme with affinity in the nM range. Some of these inhibitors are non-toxic dietary supplements and could be readily investigated for their ability to reduce methane emissions by inhibiting F_420_-dependent enzymes (Cuccioloni *et al*. 2020). If effective, inhibitors of F_420_ dependent pathways could be provided as dietary supplements, possibly in conjunction with other strategies to inhibit the growth of hydrogenotrophic methanogens or promote the growth of other hydrogenotrophs in the rumen.

#### Nitroimidazole prodrug activation in *M. tuberculosis*

**Figure 18.**
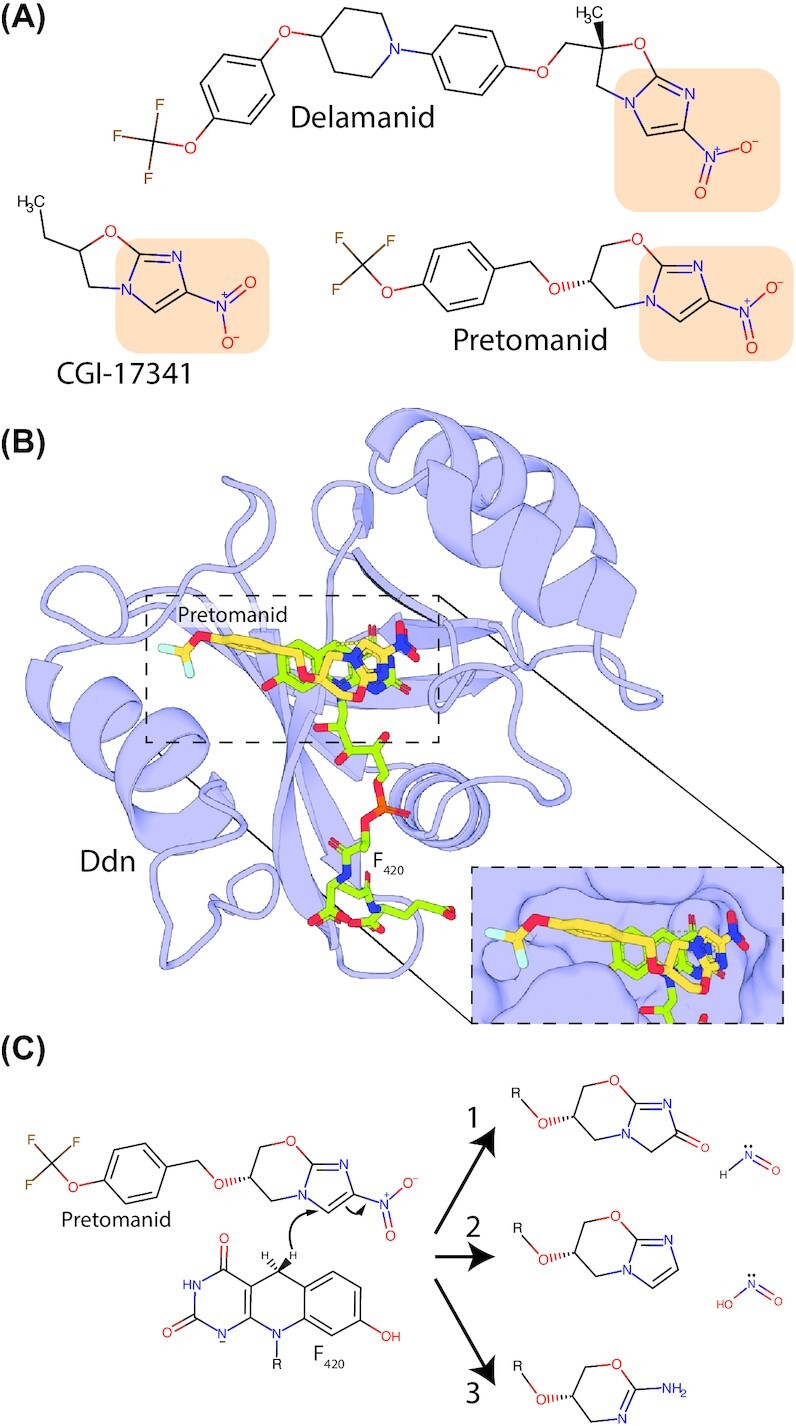
Nitroimidazole prodrugs effective against *M. tuberculosis* and their activation by Ddn. **(A)** Structure of nitroimidazole-containing prodrugs developed for tuberculosis treatment. Delamanid and pretomanid were recently approved for the treatment of *M. tuberculosis* infection, while CGI-17341 was abandoned due to toxicity concerns. The nitroimidazole functional group is highlighted in orange. **(B)** The complex between pretomanid and its activating enzyme Ddn from *M. tuberculosis*was generated by molecular docking using AutoDock Vina (Trott and Olson 2010). The proximity between the nitroimidazole group of pretomanid and the hydride transferring C5 carbon of F_420_ is shown in the inset panel. **(C)** Proposed products for the breakdown of pretomanid following reduction by Ddn, full reaction schemes leading to product generation refer to Singh *et al*. (2008).

It has been recognized since the late 1980s that compounds containing the nitroimidazole functional group can display potent antitubercular activity (Nagarajan *et al*. 1989; Liu *et al*. 2018). Early attempts to develop nitroimidazole-containing molecules for tuberculosis treatment, including the compound CGI-17341 (Ashtekar *et al*. 1993; Fig. 18A), failed due to toxicity concerns. However, concerted drug development efforts have yielded two compounds with potent antitubercular activities and acceptable safety profiles. These compounds, named delamanid and pretomanid (Fig. 18A), were both recently approved for the treatment of multidrug-resistant tuberculosis (MDR) as a combination therapy (Liu *et al*. 2018; Keam 2019). Delamanid was approved in 2014 by the European Medicines Agency for use in an appropriate combination regimen for MDR tuberculosis treatment and is subject to ongoing safety and efficacy studies (Ryan and Lo 2014; von Groote-Bidlingmaier *et al*. 2019). Pretomanid was approved in 2019 by the U.S. Food & Drug Administration for treatment of MDR or extensively drug-resistant tuberculosis (XDR), as part of a three-drug ‘BPaL’ regime, also including bedaquiline and linezolid (Keam 2019). Safety and efficacy trials indicate pretomanid, administered in combination with bedaquiline, moxifloxacin and pyrazinamide, is also effective in the treatment of MDR and XDR tuberculosis, although this regime is awaiting regulatory approval (Tweed *et al*. 2019).

Nitroimidazole-containing compounds act as prodrugs in the treatment of tuberculosis and are reductively activated in the *M. tuberculosis* cell by the aforementioned FDOR-A enzyme Ddn (Fig. 18B; Cellitti *et al*. 2012; Fujiwara *et al*. 2018). The activation of delamanid and pretomanid occurs through the promiscuous activity of Ddn, which appears to play a physiological role in the reduction of menaquinone (Lee *et al*. 2020). The reduction of the nitroimidazole functional group by Ddn is thought to lead to several reaction products, including the release of HNO and HNO_2_ and the formation of des-nitro forms of the drugs (Fig. 18C; Singh *et al*. 2008). While the precise details of the mechanism of action of delamanid and pretomanid towards *M. tuberculosis* remain to be elucidated, evidence supports a role for the release of reactive nitrogen species in their toxicity (Manjunatha *et al*. 2006; Singh *et al*. 2008; Manjunatha, Boshoff and Barry 2009). Pretomanid also selectively inhibits the synthesis of ketomycolic acids in the *M. tuberculosis*, potentially through the direct or indirect inhibition of the F_420_-reducing dehydrogenase fHMAD (Stover *et al*. 2000; Purwantini and Mukhopadhyay 2013). Further, global metabolomic analysis of pretomanid treated *M. smegmatis* identified the accumulation of the toxic metabolite methylglyoxal, indicating that metabolic poisoning may also play a role in the antitubercular activity of nitroimidazole drugs (Baptista *et al*. 2018). As nitroimidazole drugs are effective against *M. tuberculosis* under both aerobic and anaerobic conditions, and their activation by Ddn leads to several reactive intermediates and reaction products, the mechanism of action of these compounds is likely complex and multifaceted (Singh *et al*. 2008; Mukherjee and Boshoff 2011).

Resistance to delamanid and pretomanid in *M. tuberculosis* is imparted by mutations that prevent the production of F_420_ (via loss of functional FbiA, FbiB, FbiC or FbiD) or its reduction to F_420_H_2_ (via loss of functional Fgd; Choi *et al*. 2001; Manjunatha *et al*. 2006; Jing *et al*. 2019; Lee *et al*. 2020). Additionally, mutations that result in either the complete loss of Ddn function or loss of its promiscuous activity towards the prodrugs also result in resistance (Lee *et al*. 2020). The latter finding is of concern for the longevity of delamanid and pretomanid for treatment of tuberculosis. Particularly concerning is that clinical *M. tuberculosis* isolates with mutations in Ddn that abolish its activity towards delamanid and pretomanid, but not its physiological substrate menaquinone, have been identified (Lee *et al*. 2020; Rifat *et al*. 2020). A number of these isolates were from patients that had not been treated with delamanid and pretomanid, suggesting that inherently resistant strains of *M. tuberculosis* exist (Yang *et al*. 2018; Lee *et al*. 2020). Interestingly, some mutations in Ddn result in resistance to pretomanid but not delamanid (Lee *et al*. 2020), likely due to the differences in their interactions with the Ddn substrate-binding pocket. Based on this observation, a robust understanding of Ddn substrate binding may allow for the deployment of patient-tailored prodrug variants less susceptible to polymorphisms in Ddn. As discussed in the next section, the effect of the loss of F_420_ on the virulence and transmissibility of *M. tuberculosis* as a result of nitroimidazole treatment remains uncertain.

#### Targeting F_420_ for the development of antitubercular drugs

The role of F_420_ in activating pretomanid and delamanid has rekindled interest in the role of F_420_ in mycobacterial physiology (Cellitti *et al*. 2012; Haver *et al*. 2015; Fujiwara *et al*. 2018; Lee *et al*. 2020; Rifat *et al*. 2020). However, despite recent progress in this area, we lack a comprehensive understanding of the importance of F_420_ in mycobacterial physiology. This is especially true for mycobacterial pathogens like *M. tuberculosis*, in part due to the difficulty in working with this slow-growing and highly pathogenic bacterium (Cole *et al*. 1998). However, phenotypes associated with the loss of F_420_ production discussed above strongly suggest that the cofactor plays a role in the ability of *M. tuberculosis* to cause disease (Gurumurthy *et al*. 2013; Jirapanjawat *et al*. 2016; Lee *et al*. 2020; Rifat *et al*. 2020). These observations, together with the ubiquity of F_420_ in mycobacteria, the abundance of F_420_-dependent enzymes in *M. tuberculosis* and the absence of the cofactor from human cells, make the processes that produce or use F_420_ potential targets for the development of antimicrobial compounds. One method of targeting F_420_ would be the development of compounds that inhibit the enzymes responsible for its biosynthesis (i.e. FbiA, FbiB, FbiC and FbiD) or reduction (Fgd; Bashiri *et al*. 2008, 2019). Crystal structures are available for the majority of these enzymes, facilitating inhibitor design (Bashiri *et al*. 2008, 2019; Grinter *et al*. 2020). An alternative approach would be the development of F_420_ analogs that inhibit F_420_-dependent enzymes (Eirich, Vogels and Wolfe 1978). However, to make the considerable effort required for the identification and optimization of F_420_ biosynthesis inhibitors attractive, a better understanding of the importance of the cofactor for virulence is required.

The role of F_420_-dependent enzymes in reductively detoxifying antimicrobial compounds is also of interest for *M. tuberculosis* treatment (Jirapanjawat *et al*. 2016; Rifat *et al*. 2020). Profiling the antibiotic sensitivity of F_420_-deficient mutants of mycobacterial pathogens may identify antibiotics that selectively display activity against these strains. There is some evidence that loss of F_420_ leads to a heightened sensitivity to the antibiotics isoniazid, moxifloxacin and clofazimine in *M. tuberculosis* (Gurumurthy *et al*. 2013; Rifat *et al*. 2020). Given that loss of F_420_ production is known to mediate resistance to pretomanid and delamanid (Haver *et al*. 2015; Jing *et al*. 2019; Lee *et al*. 2020), antibiotics that are more effective against F_420_ deficient mutants may be useful in combination with these drugs to reduce or mitigate the development of resistance. Preliminary analysis of F_420-_dependent LLHTs in pathogenic mycobacteria demonstrates they play an important role in outer-envelope lipid biosynthesis. Outer envelope lipids, like PDIMs and ketomycolic acids, are important for mycobacterial virulence (Purwantini and Mukhopadhyay 2013; Purwantini, Daniels and Mukhopadhyay 2016). Growing evidence indicates that these outer envelope lipids constitute a second outer membrane in mycobacteria (Hoffmann *et al*. 2008; Bansal-Mutalik and Nikaido 2014). In *M. tuberculosis* this membrane contains high concentrations of PDIM, which contribute to the impermeability of this barrier and the antibiotic resistance of this species (Wang *et al*. 2020). As such, a systematic understanding of the role of F_420-_dependent enzymes in outer-envelope lipid biosynthesis will inform future efforts to combat pathogenic mycobacteria through inhibition of this process.

## OUTLOOK

When we first reviewed this topic five years ago (Greening *et al*. 2016), we noted multiple knowledge gaps that have since been addressed. These included: resolving the chemical steps and structural basis of F_420_ biosynthesis (Bashiri *et al*. 2019; Braga *et al*. 2019; Grinter *et al*. 2020); surveying the distribution of F_420_ across different taxa and ecosystems (Ney *et al*. 2017a); investigating the chemistry of the F_420_ headgroup and tail to catalysis (Mohamed *et al*. 2016a; Ney *et al*. 2017b); and enabling F_420_-dependent industrial biocatalysis through achieving heterologous cofactor production (Bashiri *et al*. 2019; Braga *et al*. 2019; Ney 2019) and characterizing promising F_420_-dependent oxidoreductases (Greening *et al*. 2017; Mascotti *et al*. 2018; Mathew *et al*. 2018; Drenth, Trajkovic and Fraaije 2019; Martin *et al*. 2020). Some of these lines of investigation resulted in unexpected findings, most notably that F_420_ biosynthesis genes are extremely widely distributed (Ney *et al*. 2017a; Table S2, Supporting Information), the biosynthesis pathway has multiple variants and was misannotated in bacteria (Bashiri *et al*. 2019; Braga *et al*. 2019, 2020; Grinter *et al*. 2020) and some bacteria produce entirely novel variants of this cofactor (Braga *et al*. 2019).

Despite these important insights, we still lack a systematic understanding of the physiological role of the cofactor. Unanswered questions include why mycobacteria encode a multitude of predicted FDORs and LLHTs, and why newly identified F_420_ producers such as Proteobacteria and Chloroflexi synthesize this cofactor. Further research is needed to resolve whether lineages such as the TACK and Asgard archaea, Firmicutes and Tectomicrobia do produce F_420_ as predicted, and if so, which variants do they make and through which pathways? Other knowledge gaps that could be addressed in coming years include a more detailed understanding of the evolution of the F_420_ biosynthetic pathway, structural resolution of F_O_ biosynthesis and newly discovered F_420_-dependent methanogen enzymes, as well as the long-standing question of which enzyme mediates 2-phospholactate production in methanogens. As we detailed in the final section of this review, there is also ample potential to translate this fundamental knowledge to address medical, environmental and industrial challenges to improve human health and sustainability. Exactly 50 years since its discovery by Wolfe and colleagues, it is increasingly clear that F_420_ is a widespread and versatile cofactor, fundamental to the physiology of many bacteria and archaea.

## References

[bib1] Abken H-J , Deppenmeier U. Purification and properties of an F_420_H_2_ dehydrogenase from *Methanosarcina mazei* Gö1. FEMS Microbiol Lett. 1997;154:231–7.

[bib2] Adam PS , Borrel G, Gribaldo S. An archaeal origin of the Wood–Ljungdahl H_4_MPT branch and the emergence of bacterial methylotrophy. Nat Microbiol. 2019;4:2155–63. 31451772 10.1038/s41564-019-0534-2

[bib3] Afting C , Hochheimer A, Thauer R. Function of H_2_-forming methylenetetrahydromethanopterin dehydrogenase from *Methanobacterium thermoautotrophicum* in coenzyme F_420_ reduction with H2. Arch Microbiol. 1998;169:206–10. 9477254 10.1007/s002030050562

[bib4] Ahmed FH , Carr PD, Lee BM et al. Sequence–structure–function classification of a catalytically diverse oxidoreductase superfamily in Mycobacteria. J Mol Biol. 2015;427:3554–71. 26434506 10.1016/j.jmb.2015.09.021

[bib5] Ahmed FH , Mohamed AE, Carr PD et al. Rv2074 is a novel F_420_H_2_-dependent biliverdin reductase in *Mycobacterium tuberculosis*. Protein Sci. 2016;25:1692–709. 27364382 10.1002/pro.2975 PMC5338246

[bib6] Albertsen M , Hugenholtz P, Skarshewski A et al. Genome sequences of rare, uncultured bacteria obtained by differential coverage binning of multiple metagenomes. Nat Biotechnol. 2013;31:533–8. 23707974 10.1038/nbt.2579

[bib7] Allegretti M , Mills DJ, McMullan G et al. Atomic model of the F_420_-reducing [NiFe] hydrogenase by electron cryo-microscopy using a direct electron detector. Elife. 2014;3:e01963. 24569482 10.7554/eLife.01963 PMC3930138

[bib8] Arshad A , Speth DR, de Graaf RM et al. A metagenomics-based metabolic model of nitrate-dependent anaerobic oxidation of methane by methanoperedens-like archaea. Front Microbiol. 2015;6:e1423. 10.3389/fmicb.2015.01423 PMC4683180 26733968

[bib9] Ashtekar DR , Costa-Perira R, Nagrajan K et al. In vitro and in vivo activities of the nitroimidazole CGI 17341 against *Mycobacterium tuberculosis*. Antimicrob Agents Chemother. 1993;37:183–6. 8452346 10.1128/aac.37.2.183 PMC187635

[bib10] Attwood G , Altermann E, Kelly W et al. Exploring rumen methanogen genomes to identify targets for methane mitigation strategies. Anim Feed Sci Technol. 2011;166-167:65–75.

[bib11] Aufhammer SW , Warkentin E, Berk H et al. Coenzyme binding in F_420_-dependent secondary alcohol dehydrogenase, a member of the bacterial luciferase family. Structure. 2004;12:361–70. 15016352 10.1016/j.str.2004.02.010

[bib12] Aufhammer SW , Warkentin E, Ermler U et al. Crystal structure of methylenetetrahydromethanopterin reductase (Mer) in complex with coenzyme F_420_: architecture of the F_420_/FMN binding site of enzymes within the nonprolyl cis-peptide containing bacterial luciferase family. Protein Sci. 2005;14:1840–9. 15937276 10.1110/ps.041289805 PMC2253363

[bib13] Bacher A , Eberhardt S, Fischer M et al. Biosynthesis of vitamin B2 (riboflavin). Annu Rev Nutr. 2000;20:153–67. 10940330 10.1146/annurev.nutr.20.1.153

[bib14] Bair TB , Isabelle DW, Daniels L. Structures of coenzyme F_420_ in *Mycobacterium* species. Arch Microbiol. 2001;176:37–43. 11479701 10.1007/s002030100290

[bib15] Bansal-Mutalik R , Nikaido H. Mycobacterial outer membrane is a lipid bilayer and the inner membrane is unusually rich in diacyl phosphatidylinositol dimannosides. Proc Natl Acad Sci. 2014;111:4958–63. 24639491 10.1073/pnas.1403078111 PMC3977252

[bib16] Bapteste É , Brochier C, Boucher Y. Higher-level classification of the Archaea: evolution of methanogenesis and methanogens. Archaea. 2005;1:353. 15876569 10.1155/2005/859728 PMC2685549

[bib17] Baptista R , Fazakerley DM, Beckmann M et al. Untargeted metabolomics reveals a new mode of action of pretomanid (PA-824). Sci Rep. 2018;8:1–7. 29572459 10.1038/s41598-018-23110-1 PMC5865180

[bib18] Barber RD , Zhang L, Harnack M et al. Complete genome sequence of *Methanosaeta concilii*, a specialist in aceticlastic methanogenesis. J Bacteriol. 2011;193:3668–9. 21571998 10.1128/JB.05031-11 PMC3133334

[bib19] Baresi L , Wolfe RS. Levels of coenzyme F_420_, coenzyme M, hydrogenase, and methylcoenzyme M methylreductase in acetate-grown *Methanosarcina*. Appl Environ Microbiol. 1981;41:388–91. 6786217 10.1128/aem.41.2.388-391.1981 PMC243705

[bib20] Baron SF , Ferry JG. Reconstitution and properties of a coenzyme F_420_-mediated formate hydrogenlyase system in *Methanobacterium formicicum*. J Bacteriol. 1989;171:3854–9. 2661536 10.1128/jb.171.7.3854-3859.1989 PMC210135

[bib21] Bashiri G , Antoney J, Jirgis EN et al. A revised biosynthetic pathway for the cofactor F_420_ in prokaryotes. Nat Commun. 2019;10:1558. 30952857 10.1038/s41467-019-09534-x PMC6450877

[bib22] Bashiri G , Perkowski EF, Turner AP et al. Tat–dependent translocation of an F_420_–binding protein of *Mycobacterium tuberculosis*. PLoS One. 2012;7:e45003. 23110042 10.1371/journal.pone.0045003 PMC3478262

[bib23] Bashiri G , Rehan AM, Greenwood DR et al. Metabolic engineering of cofactor F_420_ production in*Mycobacterium smegmatis*. PLoS One. 2010;5:e15803. 21209917 10.1371/journal.pone.0015803 PMC3012119

[bib24] Bashiri G , Rehan AM, Sreebhavan S et al. Elongation of the poly-γ-glutamate tail of F_420_ requires both domains of the F_420_: γ-glutamyl ligase (FbiB) of *Mycobacterium tuberculosis*. J Biol Chem. 2016;291:6882–94. 26861878 10.1074/jbc.M115.689026 PMC4807274

[bib25] Bashiri G , Squire CJ, Moreland NJ et al. Crystal structures of F_420_-dependent glucose-6-phosphate dehydrogenase FGD1 involved in the activation of the anti-tuberculosis drug candidate PA-824 reveal the basis of coenzyme and substrate binding. J Biol Chem. 2008;283:17531–41. 18434308 10.1074/jbc.M801854200

[bib28] Beal EJ , House CH, Orphan VJ. Manganese-and iron-dependent marine methane oxidation. Science. 2009;325:184–7. 19589998 10.1126/science.1169984

[bib29] Becraft ED , Woyke T, Jarett J et al. Rokubacteria: genomic giants among the uncultured bacterial phyla. Front Microbiol. 2017;8:2264. 29234309 10.3389/fmicb.2017.02264 PMC5712423

[bib30] Begley TP . 7.01 - Overview and introduction. In: Liu H-W, Mander L (eds.) Comprehensive Natural Products II. Oxford: Elsevier, 2010, 1–2.

[bib31] Berghuis BA , Yu FB, Schulz F et al. Hydrogenotrophic methanogenesis in archaeal phylum Verstraetearchaeota reveals the shared ancestry of all methanogens. Proc Natl Acad Sci. 2019;116:5037–44. 30814220 10.1073/pnas.1815631116 PMC6421429

[bib32] Berk H , Thauer RK. Function of coenzyme F_420_-dependent NADP reductase in methanogenic archaea containing an NADP-dependent alcohol dehydrogenase. Arch Microbiol. 1997;168:396–402. 9325428 10.1007/s002030050514

[bib33] Bertrand JA , Auger G, Fanchon E et al. Crystal structure of UDP-N-acetylmuramoyl-L-alanine: d-glutamate ligase from *Escherichia coli*. EMBO J. 1997;16:3416–25. 9218784 10.1093/emboj/16.12.3416 PMC1169967

[bib34] Bhattarai S , Cassarini C, Lens PNL. Physiology and distribution of archaeal methanotrophs that couple anaerobic oxidation of methane with sulfate reduction. Microbiol Mol Biol Rev. 2019;83:e00074–18. 31366606 10.1128/MMBR.00074-18 PMC6710461

[bib35] Biswal BK , Au K, Cherney MM et al. The molecular structure of Rv2074, a probable pyridoxine 5'-phosphate oxidase from *Mycobacterium tuberculosis*, at 1.6 A resolution. Acta Crystallogr Sect F. 2006;62:735–42. 10.1107/S1744309106025012 PMC2242915 16880544

[bib36] Björnsson L , Hugenholtz P, Tyson GW et al. Filamentous Chloroflexi (green non-sulfur bacteria) are abundant in wastewater treatment processes with biological nutrient removal. Microbiology. 2002;148:2309–18. 12177325 10.1099/00221287-148-8-2309

[bib37] Bleicher K , Winter J. Purification and properties of F_420_- and NADP^+^-dependent alcohol dehydrogenases of *Methanogenium liminatans* and *Methanobacterium palustre*, specific for secondary alcohols. Eur J Biochem. 1991;200:43–51. 1879431 10.1111/j.1432-1033.1991.tb21046.x

[bib38] Boetius A , Ravenschlag K, Schubert CJ et al. A marine microbial consortium apparently mediating anaerobic oxidation of methane. Nature. 2000;407:623–6. 11034209 10.1038/35036572

[bib1_371_1618922901933] Bogan K , Brenner C. Biochemistry: Niacin/NAD (P). 2013.

[bib39] Borrel G , O'Toole PW, Harris HMB et al. Phylogenomic data support a seventh order of methylotrophic methanogens and provide insights into the evolution of methanogenesis. Genome Biol Evol. 2013;5:1769–80. 23985970 10.1093/gbe/evt128 PMC3814188

[bib40] Bown L , Altowairish MS, Fyans JK et al. Production of the *Streptomyces scabies* coronafacoyl phytotoxins involves a novel biosynthetic pathway with an F_420_-dependent oxidoreductase and a short-chain dehydrogenase/reductase. Mol Microbiol. 2016;101:122–35. 26991928 10.1111/mmi.13378

[bib41] Braga D , Hasan M, Kröber T et al. Redox coenzyme F_420_ biosynthesis in thermomicrobia involves reduction by stand-alone nitroreductase superfamily enzymes. Appl Environ Microbiol. 2020;86:e00457–20. 32276981 10.1128/AEM.00457-20 PMC7267189

[bib42] Braga D , Last D, Hasan M et al. Metabolic pathway rerouting in *Paraburkholderia rhizoxinica* evolved long-overlooked derivatives of coenzyme F_420_. ACS Chem Biol. 2019;14:2088–94. 31469543 10.1021/acschembio.9b00605

[bib43] Brochier-Armanet C , Forterre P, Gribaldo S. Phylogeny and evolution of the Archaea: one hundred genomes later. Curr Opin Microbiol. 2011;14:274–81. 21632276 10.1016/j.mib.2011.04.015

[bib44] Brochier C , Forterre P, Gribaldo S. Archaeal phylogeny based on proteins of the transcription and translation machineries: tackling the *Methanopyrus kandleri* paradox. Genome Biol. 2004;5:R17. 15003120 10.1186/gb-2004-5-3-r17 PMC395767

[bib45] Brüggemann H , Falinski F, Deppenmeier U. Structure of the F_420_H_2_:quinone oxidoreductase of *Archaeoglobus fulgidus*. Eur J Biochem. 2000;267:5810–4. 10971593 10.1046/j.1432-1327.2000.01657.x

[bib46] Buckel W , Thauer RK. Energy conservation via electron bifurcating ferredoxin reduction and proton/Na^+^ translocating ferredoxin oxidation. Biochimica et Biophysica Acta (BBA) Bioenergetics. 2013;1827:94–113. 22800682 10.1016/j.bbabio.2012.07.002

[bib47] Bulzu P-A , Andrei A-Ş, Salcher MM et al. Casting light on Asgardarchaeota metabolism in a sunlit microoxic niche. Nat Microbiol. 2019;4:1129–37. 30936485 10.1038/s41564-019-0404-y

[bib26] Bäumer S , Ide T, Jacobi C et al. The F_420_H_2_ dehydrogenase from *Methanosarcina mazei* Is a redox-driven proton pump closely related to NADH dehydrogenases. J Biol Chem. 2000;275:17968–73. 10751389 10.1074/jbc.M000650200

[bib27] Bäumer S , Murakami E, Brodersen J et al. The F_420_H_2_: heterodisulfide oxidoreductase system from *Methanosarcina* species: 2-hydroxyphenazine mediates electron transfer from F_420_H_2_ dehydrogenase to heterodisulfide reductase. FEBS Lett. 1998;428:295–8. 9654152 10.1016/s0014-5793(98)00555-9

[bib48] Cai C , Leu AO, Xie G-J et al. A methanotrophic archaeon couples anaerobic oxidation of methane to Fe(III) reduction. ISME J. 2018;12:1929–39. 29662147 10.1038/s41396-018-0109-x PMC6052012

[bib49] Canaan S , Sulzenbacher G, Roig-Zamboni V et al. Crystal structure of the conserved hypothetical protein Rv1155 from *Mycobacterium tuberculosis*. FEBS Lett. 2005;579:215–21. 15620716 10.1016/j.febslet.2004.11.069

[bib3_409_1618931950607] Castelle CJ , Banfield JF. Major new microbial groups expand diversity and alter our understanding of the tree of life. Cell. 2018;172:1181–97. 29522741 10.1016/j.cell.2018.02.016

[bib50] Ceh K , Demmer U, Warkentin E et al. Structural basis of the hydride transfer mechanism in F_420_-dependent methylenetetrahydromethanopterin dehydrogenase. Biochemistry. 2009;48:10098–105. 19761261 10.1021/bi901104d

[bib51] Cellitti SE , Shaffer J, Jones DH et al. Structure of Ddn, the deazaflavin-dependent nitroreductase from *Mycobacterium tuberculosis* involved in bioreductive activation of PA-824. Structure. 2012;20:101–12. 22244759 10.1016/j.str.2011.11.001 PMC3267046

[bib52] Cheeseman P , Toms-Wood A, Wolfe R. Isolation and properties of a fluorescent compound, Factor 420, from *Methanobacterium* strain MoH. J Bacteriol. 1972;112:527–31. 5079072 10.1128/jb.112.1.527-531.1972 PMC251440

[bib53] Chen S-C , Musat N, Lechtenfeld OJ et al. Anaerobic oxidation of ethane by archaea from a marine hydrocarbon seep. Nature. 2019;568:108–11. 30918404 10.1038/s41586-019-1063-0

[bib54] Choi K-P , Bair TB, Bae Y-M et al. Use of transposon Tn5367 mutagenesis and a nitroimidazopyran-based selection system to demonstrate a requirement for *fbiA* and *fbiB* in coenzyme F_420_ biosynthesis by *Mycobacterium bovis* BCG. J Bacteriol. 2001;183:7058–66. 11717263 10.1128/JB.183.24.7058-7066.2001 PMC95553

[bib55] Choi K-P , Kendrick N, Daniels L. Demonstration that *fbiC* is required by *Mycobacterium bovis* BCG for coenzyme F_420_ and F_O_ biosynthesis. J Bacteriol. 2002;184:2420–8. 11948155 10.1128/JB.184.9.2420-2428.2002 PMC134996

[bib56] Cole S , Brosch R, Parkhill J et al. Deciphering the biology of *Mycobacterium tuberculosis* from the complete genome sequence. Nature. 1998;393:537–44. 9634230 10.1038/31159

[bib57] Conrad R . The global methane cycle: recent advances in understanding the microbial processes involved. Environ Microbiol Rep. 2009;1:285–92. 23765881 10.1111/j.1758-2229.2009.00038.x

[bib58] Costa KC , Wong PM, Wang T et al. Protein complexing in a methanogen suggests electron bifurcation and electron delivery from formate to heterodisulfide reductase. Proc Natl Acad Sci. 2010;107:11050–5. 20534465 10.1073/pnas.1003653107 PMC2890747

[bib59] Cox JS , Chen B, McNeil M et al. Complex lipid determines tissue-specific replication of *Mycobacterium tuberculosis* in mice. Nature. 1999;402:79–83. 10573420 10.1038/47042

[bib60] Cuccioloni M , Bonfili L, Cecarini V et al. Structure/activity virtual screening and in vitro testing of small molecule inhibitors of 8-hydroxy-5-deazaflavin: NADPH oxidoreductase from gut methanogenic bacteria. Sci Rep. 2020;10:e13150. 10.1038/s41598-020-70042-w PMC7588429 32753591

[bib61] Daniels L , Bakhiet N, Harmon K. Widespread distribution of a 5-deazaflavin cofactor in Actinomyces and related bacteria. Syst Appl Microbiol. 1985;6:12–7.

[bib62] Darwin KH , Ehrt S, Gutierrez-Ramos J-C et al. The proteasome of *Mycobacterium tuberculosis* is required for resistance to nitric oxide. Science. 2003;302:1963–6. 14671303 10.1126/science.1091176

[bib65] Decamps L , Philmus B, Benjdia A et al. Biosynthesis of F_0_, precursor of the F_420_ cofactor, requires a unique two radical-SAM domain enzyme and tyrosine as substrate. J Am Chem Soc. 2012;134:18173–6. 23072415 10.1021/ja307762b

[bib63] de Poorter LMI , Geerts WJ, Keltjens JT. Hydrogen concentrations in methane-forming cells probed by the ratios of reduced and oxidized coenzyme F_420_. Microbiology. 2005;151:1697–705. 15870477 10.1099/mic.0.27679-0

[bib67] Deppenmeier U , Blaut M, Mahlmann A et al. Reduced coenzyme F_420_: heterodisulfide oxidoreductase, a proton-translocating redox system in methanogenic bacteria. Proc Natl Acad Sci. 1990;87:9449–53. 11607121 10.1073/pnas.87.23.9449 PMC55183

[bib68] Deppenmeier U , Lienard T, Gottschalk G. Novel reactions involved in energy conservation by methanogenic archaea. FEBS Lett. 1999;457:291–7. 10471795 10.1016/s0014-5793(99)01026-1

[bib66] Deppenmeier U . The unique biochemistry of methanogenesis. Progress in Nucleic Acid Research and Molecular Biology. Vol. 71, Academic Press, 2002, 223–83. 10.1016/s0079-6603(02)71045-3 12102556

[bib64] De Wit L , Eker A. 8-Hydroxy-5-deazaflavin-dependent electron transfer in the extreme halophile *Halobacterium cutirubrum*. FEMS Microbiol Lett. 1987;48:121–5.

[bib69] Doddema HJ , Vogels GD. Improved identification of methanogenic bacteria by fluorescence microscopy. Appl Environ Microbiol. 1978;36:752–4. 103504 10.1128/aem.36.5.752-754.1978 PMC243133

[bib70] Dolfing J , Mulder J-W. Comparison of methane production rate and coenzyme F_420_ content of methanogenic consortia in anaerobic granular sludge. Appl Environ Microbiol. 1985;49:1142–5. 16346788 10.1128/aem.49.5.1142-1145.1985 PMC238520

[bib71] Doukyu N , Ogino H. Organic solvent-tolerant enzymes. Biochem Eng J. 2010;48:270–82.

[bib72] Drenth J , Trajkovic M, Fraaije MW. Chemoenzymatic synthesis of an unnatural deazaflavin cofactor that can fuel F_420_-dependent enzymes. ACS Cat. 2019;9:6435–43.

[bib73] Dubnau E , Chan J, Raynaud C et al. Oxygenated mycolic acids are necessary for virulence of *Mycobacterium tuberculosis* in mice. Mol Microbiol. 2000;36:630–7. 10844652 10.1046/j.1365-2958.2000.01882.x

[bib74] Duin EC , Wagner T, Shima S et al. Mode of action uncovered for the specific reduction of methane emissions from ruminants by the small molecule 3-nitrooxypropanol. Proc Natl Acad Sci. 2016;113:6172–7. 27140643 10.1073/pnas.1600298113 PMC4896709

[bib75] Ebert S , Fischer P, Knackmuss H-J. Converging catabolism of 2,4,6-trinitrophenol (picric acid) and 2,4-dinitrophenol by *Nocardioides simplex* FJ2-1A. Biodegradation. 2001;12:367–76. 11995829 10.1023/a:1014447700775

[bib76] Ebert S , Rieger P-G, Knackmuss H-J. Function of coenzyme F_420_ in aerobic catabolism of 2,4,6-trinitrophenol and 2, 4-dinitrophenol by *Nocardioides simplex* FJ2-1A. J Bacteriol. 1999;181:2669–74. 10217752 10.1128/jb.181.9.2669-2674.1999 PMC93703

[bib77] Edmondson DE , Barman B, Tollin G. Importance of the N-5 position in flavine coenzymes. Properties of free and protein-bound 5-deaza analogs. Biochemistry. 1972;11:1133–8. 4622351 10.1021/bi00757a003

[bib78] Edwards T , McBride BC. New method for the isolation and identification of methanogenic bacteria. Appl Microbiol. 1975;29:540–5. 804855 10.1128/am.29.4.540-545.1975 PMC187022

[bib79] Eicher T , Hauptmann S, Speicher A. The Chemistry of Heterocycles: Structures, Reactions, Synthesis, and Applications: John Wiley & Sons, 2013.

[bib80] Eirich LD , Dugger RS. Purification and properties of an F_420_-dependent NADP reductase from *Methanobacterium thermoautotrophicum*. Biochimica et Biophysica Acta (BBA) Gen Sub. 1984;802:454–8.

[bib81] Eirich LD , Vogels G, Wolfe R. Distribution of coenzyme F_420_ and properties of its hydrolytic fragments. J Bacteriol. 1979;140:20–7. 40952 10.1128/jb.140.1.20-27.1979 PMC216774

[bib82] Eirich LD , Vogels GD, Wolfe RS. Proposed structure for coenzyme F_420_ from Methanobacterium. Biochemistry. 1978;17:4583–93. 728375 10.1021/bi00615a002

[bib83] Eker A , Hessels J, Meerwaldt R. Characterization of an 8-hydroxy-5-deazaflavin: NADPH oxidoreductase from *Streptomyces griseus*. Biochimica et Biophysica Acta (BBA) Gen Sub. 1989;990:80–6. 10.1016/s0304-4165(89)80015-7 2492438

[bib84] Evans PN , Boyd JA, Leu AO et al. An evolving view of methane metabolism in the Archaea. Nat Rev Microbiol. 2019;17:219–32. 30664670 10.1038/s41579-018-0136-7

[bib85] Evans PN , Parks DH, Chadwick GL et al. Methane metabolism in the archaeal phylum Bathyarchaeota revealed by genome-centric metagenomics. Science. 2015;350:434–8. 26494757 10.1126/science.aac7745

[bib86] Ferry JG , Kastead KA. Methanogenesis. Archaea: Mol Cell Biol. 2007:288–314.. DOI: 10.1128/9781555815516.ch13.

[bib87] Fida TT , Palamuru S, Pandey G et al. Aerobic biodegradation of 2, 4-dinitroanisole by *Nocardioides* sp. strain JS1661. Appl Environ Microbiol. 2014;80:7725–31. 25281383 10.1128/AEM.02752-14 PMC4249229

[bib88] Fiebig K , Friedrich B. Purification of the F_420_-reducing hydrogenase from *Methanosarcina barkeri* (strain Fusaro). Eur J Biochem. 1989;184:79–88. 2550229 10.1111/j.1432-1033.1989.tb14992.x

[bib89] Fisher J , Spencer R, Walsh C. Enzyme-catalyzed redox reactions with the flavin analogues 5-deazariboflavin, 5-deazariboflavin 5'-phosphate, and 5-deazariboflavin 5'-diphosphate, 5'→ 5'-adenosine ester. Biochemistry. 1976;15:1054–64. 3207 10.1021/bi00650a016

[bib90] Forouhar F , Abashidze M, Xu H et al. Molecular insights into the biosynthesis of the F_420_ coenzyme. J Biol Chem. 2008;283:11832–40. 18252724 10.1074/jbc.M710352200 PMC2431047

[bib91] Fujiwara M , Kawasaki M, Hariguchi N et al. Mechanisms of resistance to delamanid, a drug for *Mycobacterium tuberculosis*. Tuberculosis. 2018;108:186–94. 29523322 10.1016/j.tube.2017.12.006

[bib92] Glas AF , Maul MJ, Cryle M et al. The archaeal cofactor F_0_ is a light-harvesting antenna chromophore in eukaryotes. Proc Natl Acad Sci. 2009;106:11540–5. 19570997 10.1073/pnas.0812665106 PMC2704855

[bib94] Gorris L , Voet A. Structural characteristics of methanogenic cofactors in the non-methanogenic archaebacterium Archaeoglobus fulgidus. Biofactors. 1991;3:29–35. 1905547

[bib93] Gorris L . Cofactor contents of methanogenic bacteria reviewed. Biofactors. 1994;4:139–45. 7916957

[bib95] Graupner M , White RH. Biosynthesis of the phosphodiester bond in coenzyme F_420_ in the methanoarchaea. Biochemistry. 2001;40:10859–72. 11535063 10.1021/bi0107703

[bib96] Graupner M , Xu H, White RH. Characterization of the 2-phospho-L-lactate transferase enzyme involved in coenzyme F_420_ biosynthesis in *Methanococcus jannaschii*. Biochemistry. 2002;41:3754–61. 11888293 10.1021/bi011937v

[bib97] Greening C , Ahmed FH, Mohamed AE et al. Physiology, biochemistry, and applications of F_420_-and F_O_-dependent redox reactions. Microbiol Mol Biol Rev. 2016;80:451–93. 27122598 10.1128/MMBR.00070-15 PMC4867364

[bib98] Greening C , Geier R, Wang C et al. Diverse hydrogen production and consumption pathways influence methane production in ruminants. ISME J. 2019;13:2617–32. 31243332 10.1038/s41396-019-0464-2 PMC6776011

[bib99] Greening C , Jirapanjawat T, Afroze S et al. Mycobacterial F_420_H_2_-dependent reductases promiscuously reduce diverse compounds through a common mechanism. Front Microbiol. 2017;8:1000. 28620367 10.3389/fmicb.2017.01000 PMC5449967

[bib100] Grinter R , Ney B, Brammananth R et al. Cellular and structural basis of synthesis of the unique intermediate dehydro-F_420_-0 in mycobacteria. Msystems. 2020;5:e00389–20. 32430409 10.1128/mSystems.00389-20 PMC7253369

[bib102] Grochowski LL , Xu H, White RH. Identification and characterization of the 2-phospho-L-lactate guanylyltransferase involved in coenzyme F_420_ biosynthesis. Biochemistry. 2008;47:3033–7. 18260642 10.1021/bi702475t

[bib101] Grochowski LL , Xu H, White RH. Identification of lactaldehyde dehydrogenase in *Methanocaldococcus jannaschii* and its involvement in production of lactate for F_420_ biosynthesis. J Bacteriol. 2006;188:2836–44. 16585745 10.1128/JB.188.8.2836-2844.2006 PMC1447007

[bib103] Guerra-Lopez D , Daniels L, Rawat M. *Mycobacterium smegmatis* mc^2^155 *fbiC* and MSMEG_2392 are involved in triphenylmethane dye decolorization and coenzyme F_420_ biosynthesis. Microbiology. 2007;153:2724–32. 17660436 10.1099/mic.0.2006/009241-0

[bib104] Gurumurthy M , Rao M, Mukherjee T et al. A novel F_420_-dependent anti-oxidant mechanism protects *Mycobacterium tuberculosis* against oxidative stress and bactericidal agents. Mol Microbiol. 2013;87:744–55. 23240649 10.1111/mmi.12127 PMC3567243

[bib105] Guy L , Ettema TJ. The archaeal ‘TACK'superphylum and the origin of eukaryotes. Trends Microbiol. 2011;19:580–7. 22018741 10.1016/j.tim.2011.09.002

[bib106] Hagemeier CH , Shima S, Thauer RK et al. Coenzyme F420-dependent methylenetetrahydromethanopterin dehydrogenase (Mtd) from *Methanopyrus kandleri*: a methanogenic enzyme with an unusual quarternary structure. J Mol Biol. 2003;332:1047–57. 14499608 10.1016/s0022-2836(03)00949-5

[bib107] Hagemeier CH , Shima S, Warkentin E et al. Coenzyme F_420_-dependent methylenetetrahydromethanopterin dehydrogenase from *Methanopyrus kandleri*: the selenomethionine-labelled and non-labelled enzyme crystallized in two different forms. Acta Crystallogr Sect D Biol Crystallogr. 2003;59:1653–5. 12925803 10.1107/s0907444903014896

[bib108] Hallam SJ , Putnam N, Preston CM et al. Reverse methanogenesis: testing the hypothesis with environmental genomics. Science. 2004;305:1457–62. 15353801 10.1126/science.1100025

[bib109] Haroon MF , Hu S, Shi Y et al. Anaerobic oxidation of methane coupled to nitrate reduction in a novel archaeal lineage. Nature. 2013;500:567–70. 23892779 10.1038/nature12375

[bib110] Hartzell PL , Zvilius G, Escalante-Semerena JC et al. Coenzyme F_420_ dependence of the methylenetetrahydromethanopterin dehydrogenase of *Methanobacterium thermoautotrophicum*. Biochem Biophys Res Commun. 1985;133:884–90. 4084309 10.1016/0006-291x(85)91218-5

[bib111] Hasan MR , Rahman M, Jaques S et al. Glucose 6-phosphate accumulation in mycobacteria implications for a novel F_420_-dependent anti-oxidant defense system. J Biol Chem. 2010;285:19135–44. 20075070 10.1074/jbc.M109.074310 PMC2885192

[bib112] Haver HL , Chua A, Ghode P et al. Mutations in genes for the F_420_ biosynthetic pathway and a nitroreductase enzyme are the primary resistance determinants in spontaneous in vitro-selected PA-824-resistant mutants of *Mycobacterium tuberculosis*. Antimicrob Agents Chemother. 2015;59:5316–23. 26100695 10.1128/AAC.00308-15 PMC4538556

[bib113] Heiss G , Hofmann KW, Trachtmann N et al. npd gene functions of *Rhodococcus* (opacus) *erythropolis* HL PM-1 in the initial steps of 2, 4, 6-trinitrophenol degradation. Microbiology. 2002;148:799–806. 11882715 10.1099/00221287-148-3-799

[bib114] Henderson G , Cox F, Ganesh S et al. Rumen microbial community composition varies with diet and host, but a core microbiome is found across a wide geographical range. Sci Rep. 2015;5:14567. 26449758 10.1038/srep14567 PMC4598811

[bib115] Hendrickson EL , Leigh JA. Roles of coenzyme F_420_-reducing hydrogenases and hydrogen-and F_420_-dependent methylenetetrahydromethanopterin dehydrogenases in reduction of F_420_ and production of hydrogen during methanogenesis. J Bacteriol. 2008;190:4818–21. 18487331 10.1128/JB.00255-08 PMC2447022

[bib116] Hocking WP , Stokke R, Roalkvam I et al. Identification of key components in the energy metabolism of the hyperthermophilic sulfate-reducing archaeon *Archaeoglobus fulgidus* by transcriptome analyses. Front Microbiol. 2014;5:e95. 10.3389/fmicb.2014.00095 PMC3949148 24672515

[bib118] Hoffmann C , Leis A, Niederweis M et al. Disclosure of the mycobacterial outer membrane: cryo-electron tomography and vitreous sections reveal the lipid bilayer structure. Proc Natl Acad Sci. 2008;105:3963–7. 18316738 10.1073/pnas.0709530105 PMC2268800

[bib119] Hollmann F , Opperman DJ, Paul CE. Enzymatic reductions-A chemist's perspective. Angew Chem Int Ed. 2020;60:5644–65. 10.1002/anie.202001876 PMC7983917 32330347

[bib120] Hossain MS , Le CQ, Joseph E et al. Convenient synthesis of deazaflavin cofactor F_O_ and its activity in F_420_-dependent NADP reductase. Org Biomol Chem. 2015;13:5082–5. 25827330 10.1039/c5ob00365b

[bib121] Hristov AN , Oh J, Giallongo F et al. An inhibitor persistently decreased enteric methane emission from dairy cows with no negative effect on milk production. Proc Natl Acad Sci. 2015;112:10663–8. 26229078 10.1073/pnas.1504124112 PMC4553761

[bib122] Huang H , Wang S, Moll J et al. Electron bifurcation involved in the energy metabolism of the acetogenic bacterium *Moorella thermoacetica* growing on glucose or H_2_ plus CO_2_. J Bacteriol. 2012;194:3689–99. 22582275 10.1128/JB.00385-12 PMC3393501

[bib123] Hug LA , Thomas BC, Sharon I et al. Critical biogeochemical functions in the subsurface are associated with bacteria from new phyla and little studied lineages. Environ Microbiol. 2016;18:159–73. 26033198 10.1111/1462-2920.12930

[bib117] Höfer I , Crüsemann M, Radzom M et al. Insights into the biosynthesis of hormaomycin, an exceptionally complex bacterial signaling metabolite. Chem Biol. 2011;18:381–91. 21439483 10.1016/j.chembiol.2010.12.018

[bib124] Ichikawa H , Bashiri G, Kelly WL. Biosynthesis of the thiopeptins and identification of an F_420_H_2_-dependent dehydropiperidine reductase. J Am Chem Soc. 2018;140:10749–56. 30118217 10.1021/jacs.8b04238 PMC6193465

[bib125] Ide T , Bäumer S, Deppenmeier U. Energy conservation by the H_2_: heterodisulfide oxidoreductase from *Methanosarcina mazei* Gö1: identification of two proton-translocating segments. J Bacteriol. 1999;181:4076–80. 10383977 10.1128/jb.181.13.4076-4080.1999 PMC93899

[bib126] Ikeno S , Aoki D, Hamada M et al. DNA sequencing and transcriptional analysis of the kasugamycin biosynthetic gene cluster from *Streptomyces kasugaensis* M338-M1. J Antibiot (Tokyo). 2006;59:18–28. 16568715 10.1038/ja.2006.4

[bib127] Ilina Y , Lorent C, Katz S et al. X-ray crystallography and vibrational spectroscopy reveal the key determinants of biocatalytic dihydrogen cycling by [NiFe] hydrogenases. Angew Chem Int Ed. 2019;58:18710–4. 10.1002/anie.201908258 PMC6916344 31591784

[bib128] Imachi H , Nobu MK, Nakahara N et al. Isolation of an archaeon at the prokaryote–eukaryote interface. Nature. 2020;577:519–25. 31942073 10.1038/s41586-019-1916-6 PMC7015854

[bib129] Imlay JA . Iron-sulphur clusters and the problem with oxygen. Mol Microbiol. 2006;59:1073–82. 16430685 10.1111/j.1365-2958.2006.05028.x

[bib130] Isabelle D , Simpson DR, Daniels L. Large-scale production of coenzyme F_420_-5, 6 by using *Mycobacterium smegmatis*. Appl Environ Microbiol. 2002;68:5750–5. 12406775 10.1128/AEM.68.11.5750-5755.2002 PMC129890

[bib131] Jacobson F , Daniels L, Fox J et al. Purification and properties of an 8-hydroxy-5-deazaflavin-reducing hydrogenase from *Methanobacterium thermoautotrophicum*. J Biol Chem. 1982;257:3385–8. 7061485

[bib132] Jacobson F , Walsh C. Properties of 7, 8-didemethyl-8-hydroxy-5-deazaflavins relevant to redox coenzyme function in methanogen metabolism. Biochemistry. 1984;23:979–88.

[bib133] Jain M , Petzold CJ, Schelle MW et al. Lipidomics reveals control of *Mycobacterium tuberculosis* virulence lipids via metabolic coupling. Proc Natl Acad Sci. 2007;104:5133–8. 17360366 10.1073/pnas.0610634104 PMC1829275

[bib134] Jay ZJ , Beam JP, Dlakić M et al. Marsarchaeota are an aerobic archaeal lineage abundant in geothermal iron oxide microbial mats. Nat Microbiol. 2018;3:732–40. 29760463 10.1038/s41564-018-0163-1

[bib135] Jing W , Zhang T, Zong Z et al. Comparison of in vitro activity of the nitroimidazoles delamanid and pretomanid against multidrug-resistant and extensively drug-resistant tuberculosis. Eur J Clin Microbiol Infect Dis. 2019;38:1293–6. 30953211 10.1007/s10096-019-03551-w

[bib136] Jirapanjawat T , Ney B, Taylor MC et al. The redox cofactor F_420_ protects mycobacteria from diverse antimicrobial compounds and mediates a reductive detoxification system. Appl Environ Microbiol. 2016;82:6810–8. 27637879 10.1128/AEM.02500-16 PMC5103081

[bib137] Johnson EF , Mukhopadhyay B. A new type of sulfite reductase, a novel coenzyme F_420_-dependent enzyme, from the methanarchaeon *Methanocaldococcus jannaschii*. J Biol Chem. 2005;280:38776–86. 16048999 10.1074/jbc.M503492200

[bib139] Johnson EF , Mukhopadhyay B. A novel coenzyme F_420_ dependent sulfite reductase and a small sulfite reductase in methanogenic archaea. In Microbial Sulfur Metabolism: Springer, 2008b, 202–16.

[bib138] Johnson EF , Mukhopadhyay B. Coenzyme F_420_-dependent sulfite reductase-enabled sulfite detoxification and use of sulfite as a sole sulfur source by *Methanococcus maripaludis*. Appl Environ Microbiol. 2008;74:3591–5. 18378657 10.1128/AEM.00098-08 PMC2423035

[bib140] Jones J , Stadtman T. Selenium-dependent and selenium-independent formate dehydrogenases of *Methanococcus vannielii*. Separation of the two forms and characterization of the purified selenium-independent form. J Biol Chem. 1981;256:656–63. 7451465

[bib141] Joosten V , van Berkel WJH. Flavoenzymes. Curr Opin Chem Biol. 2007;11:195–202. 17275397 10.1016/j.cbpa.2007.01.010

[bib142] Jordan PA , Moore BS. Biosynthetic pathway connects cryptic ribosomally synthesized posttranslationally modified peptide genes with pyrroloquinoline alkaloids. Cell Chem Biol. 2016;23:1504–14. 27866908 10.1016/j.chembiol.2016.10.009 PMC5182094

[bib143] Joseph E , Le CQ, Nguyen T et al. Evidence of negative cooperativity and half-site reactivity within an F_420_-dependent enzyme: kinetic analysis of F_420_H_2_: NADP^+^ oxidoreductase. Biochemistry. 2016;55:1082–90. 26811861 10.1021/acs.biochem.5b00762

[bib144] Kaster A-K , Moll J, Parey K et al. Coupling of ferredoxin and heterodisulfide reduction via electron bifurcation in hydrogenotrophic methanogenic archaea. Proc Natl Acad Sci. 2011;108:2981–6. 21262829 10.1073/pnas.1016761108 PMC3041090

[bib145] Keam SJ . Pretomanid: first approval. Drugs. 2019;79:1797–803. 31583606 10.1007/s40265-019-01207-9

[bib2_563_1618931058939] Kelley LA , Mezulis S, Yates CM et al. The Phyre2 web portal for protein modeling, prediction and analysis. Nat Protoc. 2015;10:845–58. 25950237 10.1038/nprot.2015.053 PMC5298202

[bib146] Kerou M , Offre P, Valledor L et al. Proteomics and comparative genomics of *Nitrososphaera viennensis* reveal the core genome and adaptations of archaeal ammonia oxidizers. Proc Natl Acad Sci. 2016;113:E7937–46. 27864514 10.1073/pnas.1601212113 PMC5150414

[bib147] Kim ST , Sancar A. Photochemistry, photophysics, and mechanism of pyrimidine dimer repair by DNA photolyase. Photochem Photobiol. 1993;57:895–904. 8337263 10.1111/j.1751-1097.1993.tb09232.x

[bib148] Kiontke S , Gnau P, Haselsberger R et al. Structural and evolutionary aspects of antenna chromophore usage by class II photolyases. J Biol Chem. 2014;289:19659–69. 24849603 10.1074/jbc.M113.542431 PMC4094076

[bib149] Kirschke S , Bousquet P, Ciais P et al. Three decades of global methane sources and sinks. Nat Geosci. 2013;6:813–23.

[bib150] Klenk H-P , Clayton RA, Tomb J-F et al. The complete genome sequence of the hyperthermophilic, sulphate-reducing archaeon *Archaeoglobus fulgidus*. Nature. 1997;390:364–70. 9389475 10.1038/37052

[bib151] Knittel K , Boetius A. Anaerobic oxidation of methane: progress with an unknown process. Annu Rev Microbiol. 2009;63:311–34. 19575572 10.1146/annurev.micro.61.080706.093130

[bib152] Kolattukudy P , Fernandes ND, Azad A et al. Biochemistry and molecular genetics of cell-wall lipid biosynthesis in mycobacteria. Mol Microbiol. 1997;24:263–70. 9159514 10.1046/j.1365-2958.1997.3361705.x

[bib153] Kozlowski JA , Stieglmeier M, Schleper C et al. Pathways and key intermediates required for obligate aerobic ammonia-dependent chemolithotrophy in bacteria and Thaumarchaeota. ISME J. 2016;10:1836–45. 26882267 10.1038/ismej.2016.2 PMC5029154

[bib154] Kozubal MA , Romine M, deM Jennings R et al. Geoarchaeota: a new candidate phylum in the Archaea from high-temperature acidic iron mats in Yellowstone National Park. ISME J. 2013;7:622–34. 23151644 10.1038/ismej.2012.132 PMC3578567

[bib155] Krzycki JA , Kenealy WR, DeNiro MJ et al. Stable carbon iotope fractionation by *Methanosarcina barkeri* during methanogenesis from acetate, methanol, or carbon dioxide-hydrogen. Appl Environ Microbiol. 1987;53:2597–9. 16347476 10.1128/aem.53.10.2597-2599.1987 PMC204153

[bib156] Kulkarni G , Kridelbaugh DM, Guss AM et al. Hydrogen is a preferred intermediate in the energy-conserving electron transport chain of *Methanosarcina barkeri*. Proc Natl Acad Sci. 2009;106:15915–20. 19805232 10.1073/pnas.0905914106 PMC2747218

[bib158] Kumar H , Nguyen Q-T, Binda C et al. Isolation and characterization of a thermostable F_420_: NADPH oxidoreductase from *Thermobifida fusca*. J Biol Chem. 2017;292:10123–30. 28411200 10.1074/jbc.M117.787754 PMC5473218

[bib157] Kumar H . Exploring Deazaflavoenzymes as Biocatalysts: University of Groningen, 2018.

[bib159] Kunow J , Linder D, Stetter KO et al. F_420_H_2_: quinone oxidoreductase from *Archaeoglobus fulgidus*. Eur J Biochem. 1994;223:503–11. 8055920 10.1111/j.1432-1033.1994.tb19019.x

[bib160] Kunow J , Schwörer B, Stetter KO et al. A F_420_-dependent NADP reductase in the extremely thermophilic sulfate-reducing *Archaeoglobus fulgidus*. Arch Microbiol. 1993;160:199–205.

[bib161] Kuypers MM , Marchant HK, Kartal B. The microbial nitrogen-cycling network. Nat Rev Microbiol. 2018;16:263–76. 29398704 10.1038/nrmicro.2018.9

[bib162] Lackner G , Peters EE, Helfrich EJ et al. Insights into the lifestyle of uncultured bacterial natural product factories associated with marine sponges. Proc Natl Acad Sci. 2017;114:E347–56. 28049838 10.1073/pnas.1616234114 PMC5255618

[bib163] Lambrecht J , Cichocki N, Hübschmann T et al. Flow cytometric quantification, sorting and sequencing of methanogenic archaea based on F_420_ autofluorescence. Microb Cell Fact. 2017;16:180. 29084543 10.1186/s12934-017-0793-7 PMC5663091

[bib164] Lapalikar GV , Taylor MC, Warden AC et al. F_420_H_2_-dependent degradation of aflatoxin and other furanocoumarins is widespread throughout the actinomycetales. PLoS One. 2012;7:e30114. 22383957 10.1371/journal.pone.0030114 PMC3288000

[bib165] Laso-Pérez R , Wegener G, Knittel K et al. Thermophilic archaea activate butane via alkyl-coenzyme M formation. Nature. 2016;539:396–401. 27749816 10.1038/nature20152

[bib167] Leahy SC , Kelly WJ, Altermann E et al. The genome sequence of the rumen methanogen *Methanobrevibacter ruminantium* reveals new possibilities for controlling ruminant methane emissions. PLoS One. 2010;5:e8926. 20126622 10.1371/journal.pone.0008926 PMC2812497

[bib166] Le CQ , Joseph E, Nguyen T et al. Optimization of expression and purification of recombinant *Archeoglobus fulgidus* F_420_H_2_: NADP^+^ Oxidoreductase, an F_420_ cofactor dependent enzyme. Protein J. 2015;34:391–7. 26493287 10.1007/s10930-015-9633-y

[bib168] Lee BM , Harold LK, Almeida DV et al. Predicting nitroimidazole antibiotic resistance mutations in *Mycobacterium tuberculosis* with protein engineering. PLoS Pathog. 2020;16:e1008287. 32032366 10.1371/journal.ppat.1008287 PMC7032734

[bib169] Lenke H , Pieper D, Bruhn C et al. Degradation of 2,4-dinitrophenol by two *Rhodococcus erythropolis* strains, HL 24-1 and HL 24-2. Appl Environ Microbiol. 1992;58:2928–32. 1444407 10.1128/aem.58.9.2928-2932.1992 PMC183028

[bib170] Li H , Graupner M, Xu H et al. CofE catalyzes the addition of two glutamates to F_420_-0 in F_420_ coenzyme biosynthesis in *Methanococcus jannaschii*. Biochemistry. 2003;42:9771–8. 12911320 10.1021/bi034779b

[bib171] Li H , Xu H, Graham DE et al. Glutathione synthetase homologs encode α-L-glutamate ligases for methanogenic coenzyme F_420_ and tetrahydrosarcinapterin biosyntheses. Proc Natl Acad Sci. 2003;100:9785–90. 12909715 10.1073/pnas.1733391100 PMC187843

[bib174] Lin X , White R. Occurrence of coenzyme F_420_ and its gamma-monoglutamyl derivative in nonmethanogenic archaebacteria. J Bacteriol. 1986;168:444–8. 3093465 10.1128/jb.168.1.444-448.1986 PMC213475

[bib175] Liu Y , Matsumoto M, Ishida H et al. Delamanid: from discovery to its use for pulmonary multidrug-resistant tuberculosis (MDR-TB). Tuberculosis. 2018;111:20–30. 30029909 10.1016/j.tube.2018.04.008

[bib176] Liu Y , Whitman WB. Metabolic, phylogenetic, and ecological diversity of the methanogenic archaea. Ann N Y Acad Sci. 2008;1125:171–89. 18378594 10.1196/annals.1419.019

[bib172] Li W , Chou S, Khullar A et al. Cloning and characterization of the biosynthetic gene cluster for tomaymycin, an SJG-136 monomeric analog. Appl Environ Microbiol. 2009;75:2958–63. 19270147 10.1128/AEM.02325-08 PMC2681672

[bib173] Li W , Khullar A, Chou S et al. Biosynthesis of sibiromycin, a potent antitumor antibiotic. Appl Environ Microbiol. 2009;75:2869–78. 19270142 10.1128/AEM.02326-08 PMC2681668

[bib178] Lukat P , Katsuyama Y, Wenzel S et al. Biosynthesis of methyl-proline containing griselimycins, natural products with anti-tuberculosis activity. Chem Sci. 2017;8:7521–7. 29163906 10.1039/c7sc02622f PMC5676206

[bib177] López-García P , Moreira D. Eukaryogenesis, a syntrophy affair. Nat Microbiol. 2019;4:1068–70. 31222170 10.1038/s41564-019-0495-5 PMC6684364

[bib179] MacLeod F , Kindler GS, Wong HL et al. Asgard archaea: diversity, function, and evolutionary implications in a range of microbiomes. AIMS Microbiol. 2019;5:48. 31384702 10.3934/microbiol.2019.1.48 PMC6646929

[bib180] Maglica Ž , Özdemir E, McKinney JD. Single-cell tracking reveals antibiotic-induced changes in mycobacterial energy metabolism. MBio. 2015;6: e02236–14. 25691591 10.1128/mBio.02236-14 PMC4338811

[bib181] Malhotra K , Kim S-T, Walsh C et al. Roles of FAD and 8-hydroxy-5-deazaflavin chromophores in photoreactivation by *Anacystis nidulans* DNA photolyase. J Biol Chem. 1992;267:15406–11. 1639785

[bib182] Manjunatha U , Boshoff HI, Barry CE. The mechanism of action of PA-824: novel insights from transcriptional profiling. Commun Integr Biol. 2009;2:215–8. 19641733 10.4161/cib.2.3.7926 PMC2717523

[bib183] Manjunatha UH , Boshoff H, Dowd CS et al. Identification of a nitroimidazo-oxazine-specific protein involved in PA-824 resistance in *Mycobacterium tuberculosis*. Proc Natl Acad Sci. 2006;103:431–6. 16387854 10.1073/pnas.0508392103 PMC1326169

[bib184] Mao Y , Varoglu M, Sherman DH. Molecular characterization and analysis of the biosynthetic gene cluster for the antitumor antibiotic mitomycin C from *Streptomyces lavendulae* NRRL 2564. Chem Biol. 1999;6:251–63. 10099135 10.1016/S1074-5521(99)80040-4

[bib185] Marrakchi H , Lanéelle M-A, Daffé M. Mycolic acids: structures, biosynthesis, and beyond. Chem Biol. 2014;21:67–85. 24374164 10.1016/j.chembiol.2013.11.011

[bib186] Martin C , Tjallinks G, Trajkovic M et al. Facile stereoselective reduction of prochiral ketones by using an F_420_-dependent alcohol dehydrogenase. ChemBioChem. 2020;22:156–9. 32935896 10.1002/cbic.202000651 PMC7820951

[bib187] Mascotti ML , Ayub MJ, Fraaije M. On the diversity of F_420_-dependent oxidoreductases: a sequence-and structure-based classification. bioRxiv. 2020. 10.1002/prot.26170 PMC8518648 34216160

[bib188] Mascotti ML , Kumar H, Nguyen Q-T et al. Reconstructing the evolutionary history of F_420_-dependent dehydrogenases. Sci Rep. 2018;8:1–10. 30514849 10.1038/s41598-018-35590-2 PMC6279831

[bib189] Mashalidis EH , Gittis AG, Tomczak A et al. Molecular insights into the binding of coenzyme F_420_ to the conserved protein Rv1155 from *Mycobacterium tuberculosis*. Protein Sci. 2015;24:729–40. 25644473 10.1002/pro.2645 PMC4420522

[bib190] Mathew S , Trajkovic M, Kumar H et al. Enantio-and regioselective ene-reductions using F_420_H_2_-dependent enzymes. Chem Commun. 2018;54:11208–11. 10.1039/c8cc04449j 30230493

[bib191] Mayerl F , Piret J, Kiener A et al. Functional expression of 8-hydroxy-5-deazaflavin-dependent DNA photolyase from *Anacystis nidulans* in *Streptomyces coelicolor*. J Bacteriol. 1990;172:6061–5. 2120199 10.1128/jb.172.10.6061-6065.1990 PMC526930

[bib192] McCarthy AJ , Williams ST. Actinomycetes as agents of biodegradation in the environment—a review. Gene. 1992;115:189–92. 1612435 10.1016/0378-1119(92)90558-7

[bib193] McCormick J , Morton GO. Identity of cosynthetic factor I of *Streptomyces aureofaciens* and fragment F_O_ from coenzyme F_420_ of *Methanobacterium* species. J Am Chem Soc. 1982;104:4014–5.

[bib194] McCormick J , Sjolander NO, Miller PA et al. The biological reduction of 7-chloro-5a (11a)-dehydrotetracycline to 7-chloro-tetracycline by *Streptomyces aureofaciens*. J Am Chem Soc. 1958;80:6460–1.

[bib195] Miller PA , Sjolander NO, Nalesnyk S et al. Cosynthetic factor I, a factor involved in hydrogen-transfer in *Streptomyces aureofaciens*. J Am Chem Soc. 1960;82:5002–3.

[bib196] Mills DJ , Vitt S, Strauss M et al. De novo modeling of the F_420_-reducing [NiFe]-hydrogenase from a methanogenic archaeon by cryo-electron microscopy. eLife. 2013;2:e00218. 23483797 10.7554/eLife.00218 PMC3591093

[bib197] Mohamed A , Ahmed F, Arulmozhiraja S et al. Protonation state of F_420_H_2_ in the prodrug-activating deazaflavin dependent nitroreductase (Ddn) from *Mycobacterium tuberculosis*. Mol Biosyst. 2016a;12:1110. 26876228 10.1039/c6mb00033a

[bib198] Mohamed AE , Condic-Jurkic K, Ahmed FH et al. Hydrophobic shielding drives catalysis of hydride transfer in a family of F_420_H_2_-dependent enzymes. Biochemistry. 2016b;55:6908–18. 27951661 10.1021/acs.biochem.6b00683

[bib5_237_1618932662097] Momper L , Aronson HS, Amend JP. Genomic description of ‘Candidatus Abyssubacteria,’a novel subsurface lineage within the candidate phylum Hydrogenedentes. Front microbiol. 2018;9:1993. 30210471 10.3389/fmicb.2018.01993 PMC6121073

[bib201] Morgavi D , Forano E, Martin C et al. Microbial ecosystem and methanogenesis in ruminants. Animal. 2010;4:1024. 22444607 10.1017/S1751731110000546

[bib202] Mori T , Cahn JK, Wilson MC et al. Single-bacterial genomics validates rich and varied specialized metabolism of uncultivated Entotheonella sponge symbionts. Proc Natl Acad Sci. 2018;115:1718–23. 29439203 10.1073/pnas.1715496115 PMC5828601

[bib203] Mukherjee T , Boshoff H. Nitroimidazoles for the treatment of TB: past, present and future. Fut Med Chem. 2011;3:1427–54. 10.4155/fmc.11.90 PMC3225966 21879846

[bib204] Munro AW , McLean KJ. Electron transfer cofactors. In: Roberts GCK (ed.) Encyclopedia of Biophysics, Berlin, Heidelberg: Springer Berlin Heidelberg, 2013,601–6.

[bib205] Muth E , Morschel E, Klein A. Purification and characterization of an 8-hydroxy-5-deazaflavin-reducing hydrogenase from the archaebacterium *Methanococcus voltae*. Eur J Biochem. 1987;169:571–7. 3121317 10.1111/j.1432-1033.1987.tb13647.x

[bib199] Möller-Zinkhan D , Börner G, Thauer RK. Function of methanofuran, tetrahydromethanopterin, and coenzyme F_420_ in *Archaeoglobus fulgidus*. Arch Microbiol. 1989;152:362–8.

[bib200] Möller-Zinkhan D , Thauer RK. Anaerobic lactate oxidation to 3CO_2_ by *Archaeoglobus fulgidus* via the carbon monoxide dehydrogenase pathway: demonstration of the acetyl-CoA carbon-carbon cleavage reaction in cell extracts. Arch Microbiol. 1990;153:215–8.

[bib206] Nagar-Anthal KR , Worrell VE, Teal R et al. The pterin lumazine inhibits growth of methanogens and methane formation. Arch Microbiol. 1996;166:136–40.

[bib207] Nagarajan K , Shankar RG, Rajappa S et al. Nitroimidazoles XXI 2, 3-dihydro-6-nitroimidazo [2, 1-b] oxazoles with antitubercular activity. Eur J Med Chem. 1989;24:631–3.

[bib208] Naraoka T , Momoi K, Fukasawa K et al. Isolation and identification of a naturally occurring 7, 8-didemethyl-8-hydroxy-5-deazariboflavin derivative from *Mycobacterium avium*. Biochimica et Biophysica Acta (BBA) Gen Sub. 1984;797:377–80.

[bib209] Nelson-Sathi S , Sousa FL, Roettger M et al. Origins of major archaeal clades correspond to gene acquisitions from bacteria. Nature. 2015;517:77–80. 25317564 10.1038/nature13805 PMC4285555

[bib210] Nercessian O , Bienvenu N, Moreira D et al. Diversity of functional genes of methanogens, methanotrophs and sulfate reducers in deep-sea hydrothermal environments. Environ Microbiol. 2005;7:118–32. 15643942 10.1111/j.1462-2920.2004.00672.x

[bib212] Ney B , Ahmed FH, Carere CR et al. The methanogenic redox cofactor F_420_ is widely synthesized by aerobic soil bacteria. ISME J. 2017a;11:125. 27505347 10.1038/ismej.2016.100 PMC5315465

[bib213] Ney B , Carere CR, Sparling R et al. Cofactor tail length modulates catalysis of bacterial F_420_-dependent oxidoreductases. Front Microbiol. 2017b;8:1902. 29021791 10.3389/fmicb.2017.01902 PMC5623714

[bib211] Ney B . Characterisation and Industrial Application of Mycobacterial F_420_ Biosynthesis Volume Bachelor of Science (Honours. *)*: Australian National University, 2019.

[bib214] Nguyen LA , He H, Pham-Huy C. Chiral drugs: an overview. Int J Biomed Sci IJBS. 2006;2:85. 23674971 PMC3614593

[bib215] Nguyen Q-T , Trinco G, Binda C et al. Discovery and characterization of an F_420_-dependent glucose-6-phosphate dehydrogenase (Rh-FGD1) from *Rhodococcus jostii* RHA1. Appl Microbiol Biotechnol. 2017;101:2831–42. 27966048 10.1007/s00253-016-8038-y PMC5352752

[bib216] Nocek B , Evdokimova E, Proudfoot M et al. Structure of an amide bond forming F_420_: γγ-glutamyl ligase from *Archaeoglobus fulgidus*-a member of a new family of non-ribosomal peptide synthases. J Mol Biol. 2007;372:456–69. 17669425 10.1016/j.jmb.2007.06.063 PMC2678844

[bib217] O'Brien D , Weinstock L, Cheng C. 10-deazariboflavin. Chem Ind. 1967;48:2044. 6064685

[bib218] O'Brien DE , Weinslock LT, Cheng C. Synthesis of 10-deazariboflavin and related 2, 4-Dioxopyrimido [4, 5-b] quinolines. J Heterocycl Chem. 1970;7:99–105.

[bib219] Orsi WD , Vuillemin A, Rodriguez P et al. Metabolic activity analyses demonstrate that *Lokiarchaeon* exhibits homoacetogenesis in sulfidic marine sediments. Nat Microbiol. 2020;5:248–55. 31873205 10.1038/s41564-019-0630-3

[bib220] Oyugi MA , Bashiri G, Baker EN et al. Mechanistic insights into F_420_-dependent glucose-6-phosphate dehydrogenase using isotope effects and substrate inhibition studies. Biochimica et Biophysica Acta (BBA) Proteins Proteomics. 2018;1866:387–95. 28807886 10.1016/j.bbapap.2017.08.001 PMC5985966

[bib221] Patel RN . Biocatalytic synthesis of intermediates for the synthesis of chiral drug substances. Curr Opin Biotechnol. 2001;12:587–604. 11849941 10.1016/s0958-1669(01)00266-x

[bib222] Patra A , Park T, Kim M et al. Rumen methanogens and mitigation of methane emission by anti-methanogenic compounds and substances. J Anim Sci Biotechnol. 2017;8:1–18. 28149512 10.1186/s40104-017-0145-9 PMC5270371

[bib223] Peck MW . Changes in concentrations of coenzyme F_420_ analogs during batch growth of *Methanosarcina barkeri* and *Methanosarcina mazei*. Appl Environ Microbiol. 1989;55:940–5. 2729992 10.1128/aem.55.4.940-945.1989 PMC184228

[bib224] Peschke U , Schmidt H, Zhang HZ et al. Molecular characterization of the lincomycin-production gene cluster of *Streptomyces lincolnensis* 78-11. Mol Microbiol. 1995;16:1137–56. 8577249 10.1111/j.1365-2958.1995.tb02338.x

[bib225] Philmus B , Decamps L, Berteau O et al. Biosynthetic versatility and coordinated action of 5′-deoxyadenosyl radicals in deazaflavin biosynthesis. J Am Chem Soc. 2015;137:5406–13. 25781338 10.1021/ja513287k PMC4416281

[bib228] Purwantini E , Daniels L, Mukhopadhyay B. F_420_H_2_ is required for phthiocerol dimycocerosate synthesis in mycobacteria. J Bacteriol. 2016;198:2020–8. 27185825 10.1128/JB.01035-15 PMC4944228

[bib227] Purwantini E , Daniels L. Molecular analysis of the gene encoding F_420_-dependent glucose-6-phosphate dehydrogenase from*Mycobacterium smegmatis*. J Bacteriol. 1998;180:2212–9. 9555906 10.1128/jb.180.8.2212-2219.1998 PMC107150

[bib226] Purwantini E , Daniels L. Purification of a novel coenzyme F_420_-dependent glucose-6-phosphate dehydrogenase from *Mycobacterium smegmatis*. J Bacteriol. 1996;178:2861–6. 8631674 10.1128/jb.178.10.2861-2866.1996 PMC178021

[bib229] Purwantini E , Gillis TP, Daniels L. Presence of F_420_-dependent glucose-6-phosphate dehydrogenase in *Mycobacterium* and *Nocardia* species, but absence from *Streptomyces* and *Corynebacterium* species and methanogenic Archaea. FEMS Microbiol Lett. 1997;146:129–34. 8997717 10.1111/j.1574-6968.1997.tb10182.x

[bib230] Purwantini E , Mukhopadhyay B. Conversion of NO_2_ to NO by reduced coenzyme F_420_ protects mycobacteria from nitrosative damage. Proc Natl Acad Sci. 2009;106:6333–8. 19325122 10.1073/pnas.0812883106 PMC2669391

[bib231] Purwantini E , Mukhopadhyay B. Rv0132c of *Mycobacterium tuberculosis* encodes a coenzyme F_420_-dependent hydroxymycolic acid dehydrogenase. PLoS One. 2013;8:e81985. 24349169 10.1371/journal.pone.0081985 PMC3859598

[bib232] Quigley J , Hughitt VK, Velikovsky CA et al. The cell wall lipid PDIM contributes to phagosomal escape and host cell exit of *Mycobacterium tuberculosis*. MBio. 2017;8. 10.1128/mBio.00148-17 PMC5340868 28270579

[bib233] Reeburgh WS . Oceanic methane biogeochemistry. Chem Rev. 2007;107:486–513. 17261072 10.1021/cr050362v

[bib234] Reji L , Francis CA. Metagenome-assembled genomes reveal unique metabolic adaptations of a basal marine Thaumarchaeota lineage. ISME J. 2020:1–11. 10.1038/s41396-020-0675-6 PMC7368007 32405026

[bib235] Ren M , Feng X, Huang Y et al. Phylogenomics suggests oxygen availability as a driving force in Thaumarchaeota evolution. ISME J. 2019;13:2150–61. 31024152 10.1038/s41396-019-0418-8 PMC6776046

[bib236] Rifat D , Li S-Y, Ioerger TR et al. Mutations in *fbiD* (Rv2983) as a novel determinant of resistance to pretomanid and delamanid in *Mycobacterium tuberculosis*. Antimicrob Agents Chemother. 2020;65:e01948–20. 33077652 10.1128/AAC.01948-20 PMC7927868

[bib237] Ryan NJ , Lo JH. Delamanid: first global approval. Drugs. 2014;74:1041–5. 24923253 10.1007/s40265-014-0241-5

[bib238] Sambandan D , Dao DN, Weinrick BC et al. Keto-mycolic acid-dependent pellicle formation confers tolerance to drug-sensitive *Mycobacterium tuberculosis*. mBio. 2013;4:e00222–13. 23653446 10.1128/mBio.00222-13 PMC3663190

[bib239] Sancar A . Structure and function of DNA photolyase. Biochemistry. 1994;33:2–9. 8286340 10.1021/bi00167a001

[bib240] Sancar GB . DNA photolyases: physical properties, action mechanism, and roles in dark repair. Mut Res DNA Repair. 1990;236:147–60. 2204823 10.1016/0921-8777(90)90002-m

[bib241] Schauer NL , Ferry JG. Composition of the coenzyme F_420_-dependent formate dehydrogenase from *Methanobacterium formicicum*. J Bacteriol. 1986;165:405–11. 3944055 10.1128/jb.165.2.405-411.1986 PMC214432

[bib242] Schmitz RA , Linder D, Stetter KO et al. N5,N10-Methylenetetrahydromethanopterin reductase (coenzyme F_420_-dependent) and formylmethanofuran dehydrogenase from the hyperthermophile*Archaeoglobus fulgidus*. Arch Microbiol. 1991;156:427–34.

[bib243] Schrijver AD , Mot RD. Degradation of pesticides by actinomycetes. Crit Rev Microbiol. 1999;25:85–119. 10405795 10.1080/10408419991299194

[bib244] Schwörer B , Breitung J, Klein AR et al. Formylmethanofuran: tetrahydromethanopterin formyltransferase and N5,N10-methylenetetrahydromethanopterin dehydrogenase from the sulfate-reducing *Archaeoglobus fulgidus*: similarities with the enzymes from methanogenic Archaea. Arch Microbiol. 1993;159:225–32. 8481089 10.1007/BF00248476

[bib245] Seedorf H , Dreisbach A, Hedderich R et al. F_420_H_2_ oxidase (FprA) from *Methanobrevibacter arboriphilus*, a coenzyme F_420_-dependent enzyme involved in O_2_ detoxification. Arch Microbiol. 2004;182:126–37. 15340796 10.1007/s00203-004-0675-3

[bib246] Seedorf H , Hagemeier CH, Shima S et al. Structure of coenzyme F_420_H_2_ oxidase (FprA), a di-iron flavoprotein from methanogenic Archaea catalyzing the reduction of O_2_ to H_2_O. FEBS J. 2007;274:1588–99. 17480207 10.1111/j.1742-4658.2007.05706.x

[bib247] Seitz KW , Dombrowski N, Eme L et al. Asgard archaea capable of anaerobic hydrocarbon cycling. Nat Commun. 2019;10:1–11. 31015394 10.1038/s41467-019-09364-x PMC6478937

[bib248] Sekhon BS . Chiral pesticides. J Pest Sci. 2009;34:1–12.

[bib249] Selengut JD , Haft DH. Unexpected abundance of coenzyme F_420_-dependent enzymes in *Mycobacterium tuberculosis* and other actinobacteria. J Bacteriol. 2010;192:5788–98. 20675471 10.1128/JB.00425-10 PMC2953692

[bib250] Shah MV , Antoney J, Kang SW et al. Cofactor F_420_-dependent enzymes: an under-explored resource for asymmetric redox biocatalysis. Catalysts. 2019;9:868.

[bib251] Sheng Y , Sun X, Shen Y et al. Structural and functional similarities in the ADP-forming amide bond ligase superfamily: implications for a substrate-induced conformational change in folylpolyglutamate synthetase. J Mol Biol. 2000;302:425–38. 10.1006/jmbi.2000.3987 10970743

[bib252] Shi J , Xu X, Liu PY et al. Discovery and biosynthesis of guanipiperazine from a NRPS-like pathway. Chem Sci. 2021;12:2925–30. 34164059 10.1039/d0sc06135b PMC8179380

[bib253] Shima S , Warkentin E, Grabarse W et al. Structure of coenzyme F_420_ dependent methylenetetrahydromethanopterin reductase from two methanogenic archaea. J Mol Biol. 2000;300:935–50. 10891279 10.1006/jmbi.2000.3909

[bib254] Shuber AP , Orr EC, Recny MA et al. Cloning, expression, and nucleotide sequence of the formate dehydrogenase genes from *Methanobacterium formicicum*. J Biol Chem. 1986;261:12942–7. 3531194

[bib255] Siméone R , Constant P, Malaga W et al. Molecular dissection of the biosynthetic relationship between phthiocerol and phthiodiolone dimycocerosates and their critical role in the virulence and permeability of *Mycobacterium tuberculosis*. FEBS J. 2007;274:1957–69. 17371506 10.1111/j.1742-4658.2007.05740.x

[bib256] Singh R , Manjunatha U, Boshoff HI et al. PA-824 kills nonreplicating *Mycobacterium tuberculosis* by intracellular NO release. Science. 2008;322:1392–5. 19039139 10.1126/science.1164571 PMC2723733

[bib257] Smith MR , Mah RA. Growth and methanogenesis by *Methanosarcina* strain 227 on acetate and methanol. Appl Environ Microbiol. 1978;36:870–9. 216307 10.1128/aem.36.6.870-879.1978 PMC243160

[bib258] Sorokin DY , Makarova KS, Abbas B et al. Discovery of extremely halophilic, methyl-reducing euryarchaea provides insights into the evolutionary origin of methanogenesis. Nat Microbiol. 2017;2:17081. 28555626 10.1038/nmicrobiol.2017.81 PMC5494993

[bib259] Sousa FL , Neukirchen S, Allen JF et al. Lokiarchaeon is hydrogen dependent. Nat Microbiol. 2016;1:1–3. 10.1038/nmicrobiol.2016.34 27572645

[bib260] Spaans SK , Weusthuis RA, Van Der Oost J et al. NADPH-generating systems in bacteria and archaea. Front Microbiol. 2015;6:742. 26284036 10.3389/fmicb.2015.00742 PMC4518329

[bib261] Spang A , Caceres EF, Ettema TJ. Genomic exploration of the diversity, ecology, and evolution of the archaeal domain of life. Science. 2017;357:eaaf3883. 28798101 10.1126/science.aaf3883

[bib262] Spang A , Ettema TJ. Archaeal evolution: the methanogenic roots of Archaea. Nat Microbiol. 2017;2:1–2. 10.1038/nmicrobiol.2017.109 28741608

[bib263] Spang A , Poehlein A, Offre P et al. The genome of the ammonia-oxidizing *Candidatus* Nitrososphaera gargensis: insights into metabolic versatility and environmental adaptations. Environ Microbiol. 2012;14:3122–45. 23057602 10.1111/j.1462-2920.2012.02893.x

[bib264] Spang A , Stairs CW, Dombrowski N et al. Proposal of the reverse flow model for the origin of the eukaryotic cell based on comparative analyses of Asgard archaeal metabolism. Nat Microbiol. 2019;4:1138–48. 30936488 10.1038/s41564-019-0406-9

[bib265] Speirs L , Rice DT, Petrovski S et al. The phylogeny, biodiversity, and ecology of the Chloroflexi in activated sludge. Front Microbiol. 2019;10:2015. 31572309 10.3389/fmicb.2019.02015 PMC6753630

[bib266] Spencer R , Fisher J, Walsh C. Preparation, characterization, and chemical properties of the flavin coenzyme analogues 5-deazariboflavin, 5-deazariboflavin 5'-phosphate, and 5-deazariboflavin 5'-diphosphate, 5'→ 5'-adenosine ester. Biochemistry. 1976;15:1043–53. 3206 10.1021/bi00650a015

[bib267] Steiningerova L , Kamenik Z, Gazak R et al. Different reaction specificities of F_420_H_2_-dependent reductases facilitate pyrrolobenzodiazepines and lincomycin to fit their biological targets. J Am Chem Soc. 2020;142:3440–8. 31944685 10.1021/jacs.9b11234

[bib268] Stetter KO , Lauerer G, Thomm M et al. Isolation of extremely thermophilic sulfate reducers: evidence for a novel branch of archaebacteria. Science. 1987;236:822–4. 17777850 10.1126/science.236.4803.822

[bib269] Stover CK , Warrener P, VanDevanter DR et al. A small-molecule nitroimidazopyran drug candidate for the treatment of tuberculosis. Nature. 2000;405:962–6. 10879539 10.1038/35016103

[bib270] Stuermer R , Hauer B, Hall M et al. Asymmetric bioreduction of activated C=C bonds using enoate reductases from the old yellow enzyme family. Curr Opin Chem Biol. 2007;11:203–13. 17353140 10.1016/j.cbpa.2007.02.025

[bib271] Susanti D , Loganathan U, Mukhopadhyay B. A Novel F_420_-dependent thioredoxin reductase gated by low potential FAD a tool for redox regulation in an anaerobe. J Biol Chem. 2016;291:23084–100. 27590343 10.1074/jbc.M116.750208 PMC5087728

[bib272] Tamada T , Kitadokoro K, Higuchi Y et al. Crystal structure of DNA photolyase from *Anacystis nidulans*. Nat Struct Mol Biol. 1997;4:887–91. 10.1038/nsb1197-887 9360600

[bib273] Tao M , Xu M, Zhang F et al. Functional genome mining reveals a novel class V lanthipeptide containing a D-amino acid introduced by an F_420_H_2_-dependent reductase. Angew Chem Int Ed. 2020. 10.1002/anie.202008035 32648341

[bib274] Taylor M , Scott C, Grogan G. F_420_-dependent enzymes-potential for applications in biotechnology. Trends Biotechnol. 2013;31:63–4. 23098999 10.1016/j.tibtech.2012.09.003

[bib275] Taylor MC , Jackson CJ, Tattersall DB et al. Identification and characterization of two families of F_420_H_2_-dependent reductases from Mycobacteria that catalyse aflatoxin degradation. Mol Microbiol. 2010;78:561–75. 20807200 10.1111/j.1365-2958.2010.07356.x PMC3034190

[bib276] Te Brömmelstroet B , Hensgens CM, Keltjens JT et al. Purification and characterization of coenzyme F_420_-dependent 5, 10-methylenetetrahydromethanopterin dehydrogenase from *Methanobacterium thermoautotrophicum* strain ΔH. Biochimica et Biophysica Acta (BBA) Gen Sub. 1991;1073:77–84. 10.1016/0304-4165(91)90185-j 1991149

[bib277] Te Brömmelstroet BW , Geerts WJ, Keltjens JT et al. Purification and properties of 5, 10-methylenetetrahydromethanopterin dehydrogenase and 5, 10-methylenetetrahydromethanopterin reductase, two coenzyme F_420_-dependent enzymes, from *Methanosarcina barkeri*. Biochimica et Biophysica Acta (BBA) Protein Struct Mol Enzymol. 1991;1079:293–302. 10.1016/0167-4838(91)90072-8 1911853

[bib280] Thauer RK , Jungermann K, Decker K. Energy conservation in chemotrophic anaerobic bacteria. Bacteriol Rev. 1977;41:100. 860983 10.1128/br.41.1.100-180.1977 PMC413997

[bib281] Thauer RK , Kaster A-K, Seedorf H et al. Methanogenic archaea: ecologically relevant differences in energy conservation. Nat Rev Microbiol. 2008;6:579–91. 18587410 10.1038/nrmicro1931

[bib278] Thauer RK . Biochemistry of methanogenesis: a tribute to Marjory Stephenson: 1998 Marjory Stephenson prize lecture. Microbiology. 1998;144:2377–406. 9782487 10.1099/00221287-144-9-2377

[bib279] Thauer RK . The Wolfe cycle comes full circle. Proc Natl Acad Sci. 2012;109:15084–5. 22955879 10.1073/pnas.1213193109 PMC3458314

[bib282] Timmers PH , Welte CU, Koehorst JJ et al. Reverse methanogenesis and respiration in methanotrophic archaea. Archaea, 2017;2017:1–22. 10.1155/2017/1654237 PMC5244752 28154498

[bib283] Toogood HS , Gardiner JM, Scrutton NS. Biocatalytic reductions and chemical versatility of the old yellow enzyme family of flavoprotein oxidoreductases. ChemCatChem. 2010;2:892–914.

[bib284] Tourna M , Stieglmeier M, Spang A et al. *Nitrososphaera viennensis*, an ammonia oxidizing archaeon from soil. Proc Natl Acad Sci. 2011;108:8420–5. 21525411 10.1073/pnas.1013488108 PMC3100973

[bib6_744_1618933015693] Trott O , Olson AJ. AutoDock Vina: improving the speed and accuracy of docking with a new scoring function, efficient optimization, and multithreading. J Comput Chem. 2010;31:455–61. 19499576 10.1002/jcc.21334 PMC3041641

[bib285] Tweed CD , Dawson R, Burger DA et al. Bedaquiline, moxifloxacin, pretomanid, and pyrazinamide during the first 8 weeks of treatment of patients with drug-susceptible or drug-resistant pulmonary tuberculosis: a multicentre, open-label, partially randomised, phase 2b trial. Lancet Respir Med. 2019;7:1048–58. 31732485 10.1016/S2213-2600(19)30366-2 PMC7641992

[bib286] Tzeng S , Wolfe R, Bryant M. Factor 420-dependent pyridine nucleotide-linked hydrogenase system of Methanobacterium ruminantium. J Bacteriol. 1975;121:184–91. 234934 10.1128/jb.121.1.184-191.1975 PMC285629

[bib287] Tzeng SF , Bryant MP, Wolfe RS. Factor 420-dependent pyridine nucleotide-linked formate metabolism of Methanobacterium ruminantium. J Bacteriol. 1975;121:192–6. 234935 10.1128/jb.121.1.192-196.1975 PMC285630

[bib288] Ungerfeld E , Rust S, Boone D et al. Effects of several inhibitors on pure cultures of ruminal methanogens. J Appl Microbiol. 2004;97:520–6. 15281932 10.1111/j.1365-2672.2004.02330.x

[bib289] van Beelen P , Dijkstra AC, Vogels GD. Quantitation of coenzyme F_420_ in methanogenic sludge by the use of reversed-phase high-performance liquid chromatography and a fluorescence detector. Eur J Appl Microbiol Biotechnol. 1983;18:67–9.

[bib290] Vanwonterghem I , Evans PN, Parks DH et al. Methylotrophic methanogenesis discovered in the archaeal phylum Verstraetearchaeota. Nat Microbiol. 2016;1:1–9. 10.1038/nmicrobiol.2016.170 27694807

[bib291] Vaupel M , Thauer RK. Coenzyme F_420_-dependent N5, N10-methylenetetrahydromethanopterin reductase (Mer) from *Methanobacterium thermoautotrophicum* strain Marburg: cloning, sequencing, transcriptional analysis, and functional expression in *Escherichia coli* of the mer gene. Eur J Biochem. 1995;231:773–8. 7649177 10.1111/j.1432-1033.1995.0773d.x

[bib292] Vitt S , Ma K, Warkentin E et al. The F_420_-reducing [NiFe]-hydrogenase complex from *Methanothermobacter marburgensis*, the first X-ray structure of a group 3 family member. J Mol Biol. 2014;426:2813–26. 24887099 10.1016/j.jmb.2014.05.024

[bib293] von Groote-Bidlingmaier F , Patientia R, Sanchez E et al. Efficacy and safety of delamanid in combination with an optimised background regimen for treatment of multidrug-resistant tuberculosis: a multicentre, randomised, double-blind, placebo-controlled, parallel group phase 3 trial. Lancet Respir Med. 2019;7:249–59. 30630778 10.1016/S2213-2600(18)30426-0

[bib294] Walsh C . Flavin coenzymes: at the crossroads of biological redox chemistry. Acc Chem Res. 1980;13:148–55.

[bib295] Walsh C . Naturally occurring 5-deazaflavin coenzymes: biological redox roles. Acc Chem Res. 1986;19:216–21.

[bib296] Wang F-P , Zhang Y, Chen Y et al. Methanotrophic archaea possessing diverging methane-oxidizing and electron-transporting pathways. ISME J. 2014;8:1069–78. 24335827 10.1038/ismej.2013.212 PMC3996691

[bib297] Wang P , Bashiri G, Gao X et al. Uncovering the enzymes that catalyze the final steps in oxytetracycline biosynthesis. J Am Chem Soc. 2013;135:7138–41. 23621493 10.1021/ja403516u

[bib298] Wang P , Kim W, Pickens LB et al. Heterologous expression and manipulation of three tetracycline biosynthetic pathways. Angew Chem. 2012;124:11298–302. 10.1002/anie.201205426 PMC4070387 23024027

[bib299] Wang Q , Boshoff HI, Harrison JR et al. PE/PPE proteins mediate nutrient transport across the outer membrane of Mycobacterium tuberculosis. Science. 2020;367:1147–51. 32139546 10.1126/science.aav5912 PMC11036889

[bib300] Wang Y , Wegener G, Hou J et al. Expanding anaerobic alkane metabolism in the domain of Archaea. Nat Microbiol. 2019;4:595–602. 30833728 10.1038/s41564-019-0364-2

[bib301] Warkentin E , Hagemeier CH, Shima S et al. The structure of F_420_-dependent methylenetetrahydromethanopterin dehydrogenase: a crystallographic ‘superstructure' of the selenomethionine-labelled protein crystal structure. Acta Crystallogr Sect D Biol Crystallogr. 2005;61:198–202. 15681872 10.1107/S0907444904030732

[bib302] Warkentin E , Mamat B, Sordel-Klippert M et al. Structures of F_420_H_2_:NADP^+^ oxidoreductase with and without its substrates bound. EMBO J. 2001;20:6561–9. 11726492 10.1093/emboj/20.23.6561 PMC125772

[bib303] Weiss MC , Sousa FL, Mrnjavac N et al. The physiology and habitat of the last universal common ancestor. Nat Microbiol. 2016;1:1–8. 10.1038/nmicrobiol.2016.116 27562259

[bib304] Welander PV , Metcalf WW. Loss of the mtr operon in Methanosarcina blocks growth on methanol, but not methanogenesis, and reveals an unknown methanogenic pathway. Proc Natl Acad Sci. 2005;102:10664–9. 16024727 10.1073/pnas.0502623102 PMC1180775

[bib307] Welte C , Deppenmeier U. Bioenergetics and anaerobic respiratory chains of aceticlastic methanogens. Biochimica et Biophysica Acta (BBA) Bioenerg. 2014;1837:1130–47. 10.1016/j.bbabio.2013.12.002 24333786

[bib305] Welte C , Deppenmeier U. Membrane-bound electron transport in *Methanosaeta thermophila*. J Bacteriol. 2011;193:2868–70. 21478356 10.1128/JB.00162-11 PMC3133127

[bib306] Welte C , Deppenmeier U. Re-evaluation of the function of the F_420_ dehydrogenase in electron transport of *Methanosarcina mazei*. FEBS J. 2011;278:1277–87. 21306561 10.1111/j.1742-4658.2011.08048.x

[bib308] Wichmann R , Vasic-Racki D. Cofactor regeneration at the lab scale. Technol Transf Biotechnol: Springer, 2005, 225–60. 10.1007/b98911 15791939

[bib309] Widdel F , Wolfe R. Expression of secondary alcohol dehydrogenase in methanogenic bacteria and purification of the F_420_-specific enzyme from *Methanogenium thermophilum* strain TCI. Arch Microbiol. 1989;152:322–8.

[bib310] Williams TA , Szöllősi GJ, Spang A et al. Integrative modeling of gene and genome evolution roots the archaeal tree of life. Proc Natl Acad Sci. 2017;114:E4602–11. 28533395 10.1073/pnas.1618463114 PMC5468678

[bib311] Wilson MC , Mori T, Rückert C et al. An environmental bacterial taxon with a large and distinct metabolic repertoire. Nature. 2014;506:58–62. 24476823 10.1038/nature12959

[bib312] Winkler CK , Faber K, Hall M. Biocatalytic reduction of activated CC-bonds and beyond: emerging trends. Curr Opin Chem Biol. 2018;43:97–105. 29275291 10.1016/j.cbpa.2017.12.003

[bib313] Wood GE , Haydock AK, Leigh JA. Function and regulation of the formate dehydrogenase genes of the methanogenic archaeon *Methanococcus maripaludis*. J Bacteriol. 2003;185:2548–54. 12670979 10.1128/JB.185.8.2548-2554.2003 PMC152622

[bib314] Xia K , Shen G-B, Zhu X-Q. Thermodynamics of various F_420_ coenzyme models as sources of electrons, hydride ions, hydrogen atoms and protons in acetonitrile. Org Biomol Chem. 2015;13:6255–68. 25962496 10.1039/c5ob00538h

[bib316] Yang JS , Kim KJ, Choi H et al. Delamanid, bedaquiline, and linezolid minimum inhibitory concentration distributions and resistance-related gene mutations in multidrug-resistant and extensively drug-resistant tuberculosis in Korea. Ann Lab Med. 2018;38:563–8. 30027700 10.3343/alm.2018.38.6.563 PMC6056398

[bib315] Yan Z , Wang M, Ferry JG. A ferredoxin-and F_420_H_2_-dependent, electron-bifurcating, heterodisulfide reductase with homologs in the domains bacteria and archaea. MBio. 2017;8:e02285–16.:28174314 10.1128/mBio.02285-16 PMC5296606

[bib318] Yuan Y , Zhu Y, Crane DD et al. The effect of oxygenated mycolic acid composition on cell wall function and macrophage growth in *Mycobacterium tuberculosis*. Mol Microbiol. 1998;29:1449–58. 9781881 10.1046/j.1365-2958.1998.01026.x

[bib317] Yu T , Wu W, Liang W et al. Growth of sedimentary Bathyarchaeota on lignin as an energy source. Proc Natl Acad Sci. 2018;115:6022–7. 29773709 10.1073/pnas.1718854115 PMC6003339

[bib319] Zhalnina KV , Dias R, Leonard MT et al. Genome sequence of *Candidatus* Nitrososphaera evergladensis from group I. 1b enriched from Everglades soil reveals novel genomic features of the ammonia-oxidizing archaea. PLoS One. 2014;9:e101648. 24999826 10.1371/journal.pone.0101648 PMC4084955

[bib4_821_1618932132971] Zhou Z , Liu Y, Xu W et al. Genome-and community-level interaction insights into carbon utilization and element cycling functions of Hydrothermarchaeota in hydrothermal sediment. Msystems. 2020;5. 10.1128/mSystems.00795-19 PMC6946796 31911466

[bib320] Zhou Z , Pan J, Wang F et al. Bathyarchaeota: globally distributed metabolic generalists in anoxic environments. FEMS Microbiol Rev. 2018;42:639–55. 29790926 10.1093/femsre/fuy023

[bib321] Zhu J , Zheng H, Ai G et al. The genome characteristics and predicted function of methyl-group oxidation pathway in the obligate aceticlastic methanogens, Methanosaeta spp. PLoS One. 2012;7:e36756. 22590603 10.1371/journal.pone.0036756 PMC3349665

